# Polymyxin B1 in the *Escherichia coli* inner membrane: A complex story of protein and lipopolysaccharide-mediated insertion

**DOI:** 10.1016/j.jbc.2024.107754

**Published:** 2024-09-10

**Authors:** Dhanushka Weerakoon, Jan K. Marzinek, Conrado Pedebos, Peter J. Bond, Syma Khalid

**Affiliations:** 1 School of Chemistry, University of Southampton, Southampton, UK; 2 Bioinformatics Institute (BII), Agency for Science, Technology and Research (A∗STAR), Singapore, Republic of Singapore; 3 Department of Biochemistry, University of Oxford, Porto Alegre, UK; 4 Programa de Pós-Graduação em Biociências (PPGBio), Universidade Federal de Ciências da Saudé de Porto Alegre - UFCSPA, Brazil; 5 Department of Biological Sciences, National University of Singapore, Singapore, Republic of Singapore

**Keywords:** antimicrobial peptides, Gram-negative bacteria, molecular simulation, lipopolysaccharide, multiscale modeling

## Abstract

The rise in multi-drug resistant Gram-negative bacterial infections has led to an increased need for “last-resort” antibiotics such as polymyxins. However, the emergence of polymyxin-resistant strains threatens to bring about a post-antibiotic era. Thus, there is a need to develop new polymyxin-based antibiotics, but a lack of knowledge of the mechanism of action of polymyxins hinders such efforts. It has recently been suggested that polymyxins induce cell lysis of the Gram-negative bacterial inner membrane (IM) by targeting trace amounts of lipopolysaccharide (LPS) localized there. We use multiscale molecular dynamics (MD), including long-timescale coarse-grained (CG) and all-atom (AA) simulations, to investigate the interactions of polymyxin B1 (PMB1) with bacterial IM models containing phospholipids (PLs), small quantities of LPS, and IM proteins. LPS was observed to (transiently) phase separate from PLs at multiple LPS concentrations, and associate with proteins in the IM. PMB1 spontaneously inserted into the IM and localized at the LPS-PL interface, where it cross-linked lipid headgroups *via* hydrogen bonds, sampling a wide range of interfacial environments. In the presence of membrane proteins, a small number of PMB1 molecules formed interactions with them, in a manner that was modulated by local LPS molecules. Electroporation-driven translocation of PMB1 *via* water-filled pores was favored at the protein-PL interface, supporting the 'destabilizing' role proteins may have within the IM. Overall, this in-depth characterization of PMB1 modes of interaction reveals how small amounts of mislocalized LPS may play a role in pre-lytic targeting and provides insights that may facilitate rational improvement of polymyxin-based antibiotics.

Polymyxins are a class of peptides produced by the Gram-positive bacterium *Bacillus polymyxa* (1). They are antibacterial agents often used as the “last resort” option against infections caused by carbapenem-resistant Gram-negative bacteria such as *Escherichia coli*, *Acinetobacter baumannii* and *Pseudomonas aeruginosa* (2, 3, 4). However, problems related to emerging resistance to polymyxins, as well as ongoing issues of polymyxin toxicity, necessitate the development of novel, more potent, yet less toxic alternatives (5, 6). To do so in a rational manner, it is crucial to first develop a molecular-level characterization of their mechanism of action.

Polymyxins are lipopeptides composed of 10 amino acids, arranged as a cyclic heptapeptide linked to a linear tripeptide, which is attached to a fatty acid tail (Fig. 1). Polymyxin B1 (PMB1) and E (colistin) differ by a single amino acid residue (D-Phe/D-Leu respectively) and are thought to act *via* similar mechanisms (7, 8, 9). Polymyxins are membrane-targeting lipopeptides thought to employ a self-uptake mechanism to move across the outer membrane (OM) of Gram-negative species into the periplasm (10, 11, 12, 13). The general consensus is that polymyxins cross the OM, likely causing non-fatal damage, and that bactericidal activity is then achieved by their action on the inner membrane (IM) (14, 15, 16). It has been hypothesized that polymyxins insert into the IM (a PL bilayer), forming pores or defects that eventually lead to leakage of the cellular contents into the extracellular medium and thus cell lysis.

**Figure 1:**
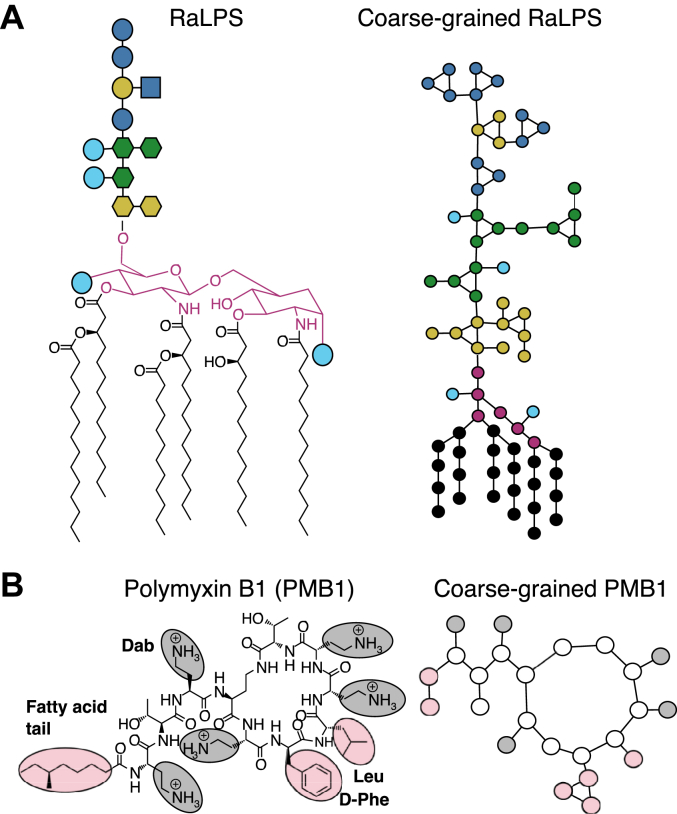
**AA and CG structures of RaLPSand PMB1.***A*, for atomistic structure, Type 1 lipid A structure chemical is shown, core oligosaccharide is represented by Symbol Nomenclature for Glycans (SNFG), phosphate groups are represented by *lightblue* circles, lipid A headgroup is highlighted in *darkpink*. The CG structure shows various parts of RaLPS corresponding to beads. *B*, AA and CG structures of PMB1. 2,4-diaminobutyric acid (Dab) residues are highlighted in *gray* and hydrophobic residues are highlighted in *pink*.

Recent lipidomics experiments indicate that lipopolysaccharide (LPS) is present in the IM in small quantities (17), due to LPS transport from the cytoplasm to the IM (flipping from the cytoplasmic to the periplasmic leaflet) (18, 19, 20) and then finally to the OM (21). Additionally, colistin-resistant *E. coli* IMs show resistance to colistin but not to putative PL-targeting antibiotics (daptomycin, nisin) (17, 22, 23), while the IM of colistin-susceptible *E. coli* is susceptible to all three antibiotics. Strikingly, accumulation of LPS in the IM, induced by murepavadin (an LptD inhibitor), correlates with higher IM colistin permeation in a *P. aeruginosa* strain (17) whereas the OMs of both colistin-susceptible and colistin-resistant strains are permeabilized by colistin (17, 18). Collectively, these findings indicate that cationic colistin preferentially binds to trace quantities of anionic LPS molecules present in the IM, relying on this interaction to bypass the IM and eventually kill the cell (17, 22). Given insertion of polymyxins into PL bilayers has been shown to occur *via* both experimental (11, 24, 25, 26) and simulation studies (27, 28, 29, 30), this presents a new potential cell lytic mode of action.

An experimental study by Khadka *et al.* investigated the impact of colistin on mixed LPS:phosphocholine (PC) (weight ratios 1:100–1:30) bilayers (31). Vesicle leakage assays and liquid atomic force microscopy (AFM) measurements indicate that in the absence of divalent cations, colistin (5–10 μM) induces vesicle leakage and formation of protruding membrane clusters (200–1000 Å) in an LPS-dependent manner. The authors have suggested that LPS clustering is induced by colistin and that solutes may permeate at the cluster edges (31). Furthermore, a recent study showed that PMB1 (0.5–10 μM) induces a larger transmembrane (TM) current in a mixed PC:phospho-(1′-rac-glycerol) (PG): deep rough lipopolysaccharide (ReLPS, lipid A + two Kdo (3-deoxy-d-manno-oct-2-ulosonic acid) residues) (49.5:49.5:1%) bilayer than in a PC:PG bilayer (albeit in the presence of a TM voltage (50 mV)), with the TM current correlating positively with PMB1 concentration (32). This suggests that the PMB1-ReLPS interaction is key for pore formation (in the presence of an electric field), although this behavior is not replicated upon the replacement of ReLPS with lipid A. Additionally, surface plasmon resonance (SPR) data shows that PMB1 can sequester lipid A in flow cytometry experiments, removing it from lipid A:PC:phosphoethanolamine (PE) (10:88:2) vesicles and lipid A(2–25%):PC monolayers (33). However, all the results discussed were obtained in the absence of explicitly added divalent cations; in fact, Khadka *et al.* found that the addition of divalent cations abolished colistin-induced membrane disruption, and since divalent ions are key in cross-linking LPS in the native OM (10), this may limit the applicability of the findings.

Due to experimental difficulties in resolving the action of polymyxins on membranes at high resolution, MD simulations have been used to investigate polymyxin-membrane interactions in both atomistic and CG levels of detail. Most MD studies to date have focused on the interaction of PMB1 and colistin with Gram-negative OM models (29, 30, 34, 35, 36, 37, 38), with a few considering interactions with the IM (27, 28, 29, 30, 39, 40, 41). In general, a range of simulations (both atomistic and CG) indicate that polymyxins first insert their hydrophobic moieties into the IM. However, what happens following this remains unclear. For example, in one atomistic simulation study, partial polymyxin translocation towards the hydrophobic membrane core was observed (similar to the ‘barrel-stave’ mode of antimicrobial peptide insertion), accompanied by extensive membrane disruption (29). In some CG MD simulations, polymyxins tend to remain inserted and aggregated on the IM, perturbing membrane properties (similar to the ‘carpet insertion’ mode of action) (28, 41). An AA simulation study of the interaction of PMB1 with highly simplified IM models presented evidence for both mechanisms, depending on lipid tail packing and membrane surface charge (27). In contrast, a biased simulation study employing umbrella sampling of colistin translocation through an *A. baumannii* LPS-deficient membrane (essentially a PL bilayer) suggests that the initial binding of polymyxins to the membrane is an unfavorable process (30). This, coupled with the lack of polymyxin translocation seen during some long-timescale simulations, suggests that polymyxins may enter the cell *via* interaction with a component not previously included in existing IM models, such as membrane proteins or small quantities of LPS, as suggested by Edwards *et al.* (17, 22).

Recent CG MD studies have investigated the interaction of polymyxins with mixed membrane models composed of PLs and ReLPS (40, 41). This is of interest as PLs can be (mis)-localized to the outer leaflet of the OM, for example in response to external stresses (40, 41). LPS and PLs tend to phase separately within the membrane and PMB1 molecules insert into membranes with <20% LPS *via* their hydrophobic residues and acyl tail. PMB1-LPS interactions are also favored over PMB1-PL interactions, particularly at lower LPS concentrations. Umbrella sampling calculations indicate that the free energy required to transfer PMB1 through the interface between phase-separated LPS and PLs is consistently lower than that through comparable OM or IM models (40). However, this behavior has not yet been observed in unbiased or AA simulations, and no comparable studies have so far incorporated membrane proteins into the membrane models.

Here, we report multi-microsecond CG and AA simulations of PMB1 molecules interacting with *E. coli* IM models incorporating small amounts (≤10%) of rough lipopolysaccharide (RaLPS) and native abundant membrane proteins, namely lactose permease (LacY) (42) or ammonia transporter B (AmtB) (43). In most systems studied, RaLPS present in the IM was observed to cluster into domains that become attached to membrane proteins when present. PMB1 molecules primarily insert into these membranes, localizing at the RaLPS-PL interface, where they take part in the formation of extensive hydrogen bonding networks, with individual PMB1 molecules interacting with RaLPS to varying extents. We find that membrane proteins have a minor effect on averaged measurements of PMB1-RaLPS interactions, but at an individual level, the presence of membrane proteins appears to have a small modulatory effect on PMB1-LPS interactions, which is dependent on LPS content. Finally, simulations of electroporation induced *via* a TM charge imbalance showed that pores were formed primarily at the protein-PL interfaces, through which PMB1 molecules could translocate.

## Results and discussion

### LPS phase separation in Gram-negative inner membrane

**Figure 2:**
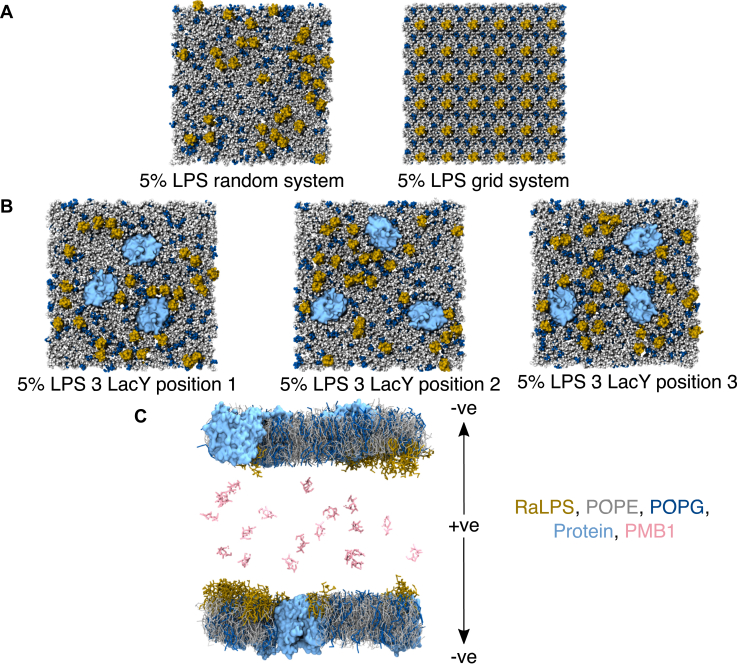
**Starting configurations of CG systems.***A*, representative starting configuration (top view) of a random 5% LPS system (*left*) and a grid 5% LPS system (*right*). *B*, starting configuration for each independent repeat of 5% LPS + LacY systems. *C*, a representative snapshot (side view) of a double bilayer system with charge gradient labelled. Molecules are represented as: RaLPS = *yellow* vdW (A, B)/licorice (C); POPE = *gray* vdW (A, B)/licorice (C); POPG = *darkblue* vdW (A, B)/licorice (C); proteins = *lightblue* surface; PMB1 = *palepink* licorice, solvent + ions hidden for clarity.

IM models containing 1-hexadecanoyl-2-(9Z-octadecenoyl)-sn-glycero-3-phosphoethanolamine (POPE), 1-hexadecanoyl-2-(9Z-octadecenoyl)-sn-glycero-3-phospho-(1′-rac-glycerol) (POPG) and small amounts of RaLPS molecules (Table 1) were first equilibrated *via* CG simulations for 5 μs (in the absence of PMB1 molecules). Systems were built either without membrane proteins (with RaLPS molecules in a grid-like or pseudo-random placement, Fig. 2*A*), with three copies of LacY (Fig. 2*B*) or with one copy of trimeric AmtB (5% LPS systems only).

**Table 1:** Summary of simulation systems

LPS content	Setup method	Proteins present	Repeats	No. of PMB1 molecules	Resolution
10%	CHARMM-GUI	None	3	0, 9–10	CG
		LacY	3	0, 9–10	
	Grid	None	3	0	
	Backmapped	None	2	10	AA
		LacY	2	10	
5%	CHARMM-GUI	None	3	0, 9–10, 20	CG
		LacY	3	0, 9–10, 20	
		AmtB	3	0, 10	
	Grid	None	3	0	
	Backmapped	None	2	20	AA
		LacY	2	20	
	Double bilayer	LacY	3 (×3)	20	CG
2%	CHARMM- GUI	None	3	0, 10	CG
		LacY	3	0, 10	
	Grid	None	3	0	
1%	CHARMM-GUI	None	3	0, 10	CG
		LacY	3	0, 10	
	Grid	None	3	0

**Figure 3:**
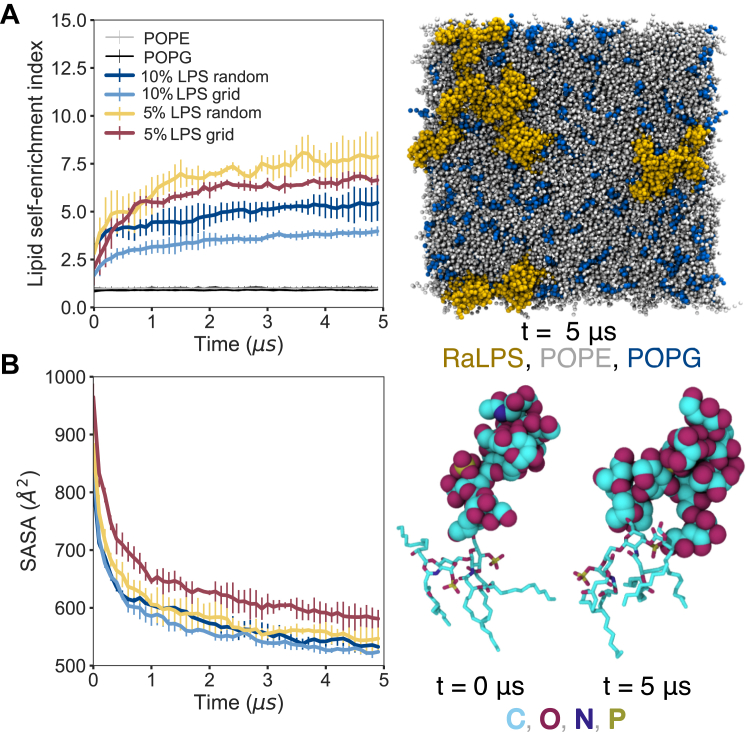
**LPS dynamics in CG simulations, in the absence of PMB1 molecules and membrane proteins.***A*, *Left*: Lipid self-enrichment index over time; *right*: snapshot of a 5% LPS grid system at 5 μs; moieties are represented as: RaLPS = *yellow* vdW, POPE = *gray* vdW, POPG = *darkblue* vdW. *B*, *Left*: RaLPS saccharide SASA per RaLPS molecule over time. SASA was calculated relative to surface area of all non-solvent beads, probe radius = 2.6 Å. *Right:* Snapshots of an LPS molecule backmapped to AA resolution for a representative system at t = 0 and t = 5 μs; moieties are represented as: RaLPS saccharide = vdW, *CPK color scheme + teal* (carbons); RaLPS tails + headgroup = licorice, *CPK color scheme + teal* (carbons). In (A) and (B), systems studied included: RaLPS in 10% random LPS systems (n = 3, *darkblue*), RaLPS in 10% LPS grid systems (n = 3, *lightblue*), RaLPS in 5% random LPS systems (n = 3, *yellow*), RaLPS in 5% grid LPS systems (n = 3, *maroon*), POPE (all systems, n = 3, *gray*), POPG (all systems, n = 3, *black*). Individual trajectories were block-averaged in 0.1 μs blocks and subsequently averaged over n repeats, and error bars show 95% confidence intervals.

Lipid self-enrichment indices (Equation 1) are a measure of how likely a lipid is to aggregate with a lipid of the same type. Self-enrichment indices were calculated for the upper, LPS-containing leaflet over the simulation time (Fig. 3*A*, Figs. S1*A*, S2). The self-enrichment indices of POPE and POPG remained ∼1 throughout, indicating that PLs in the upper leaflet were not, on average, aggregating over time. Conversely, the self-enrichment indices of RaLPS increased over time in protein-free ‘random LPS’ and ‘grid LPS’ systems containing 1 to 10% RaLPS (Figs. 3*A*, S2), indicating that RaLPS molecules were aggregating in the IM. In the absence of membrane proteins, the lipid self-enrichment index at t = 5 μs showed an increasing trend with decreasing LPS content (Figs. 3*A*, S2). In the presence of membrane proteins, similar aggregation behavior was seen in all cases, except in the case where only 1% RaLPS was present with LacY proteins, in which case RaLPS aggregation, on average, fluctuated over the course of the simulations, being in a disaggregated state at t = 5 μs in some repeat simulations (Fig. S2*B*). This may indicate that the membrane proteins have a disruptive effect on LPS aggregation at this low concentration; however, this observation may be statistically insignificant given the small number of RaLPS molecules present in these simulations (6–9 molecules) and the inherent timescale limitations.

Calculation of 2D molecular densities in the plane of the membrane for RaLPS and calcium ions, measured over the last 0.5 μs of PMB1-free simulations (Fig. S3), revealed that the two were co-localized, reflective of strong RaLPS-calcium binding and headgroup-calcium cross-linking. Furthermore, measurement of the solvent accessible surface area (SASA) per RaLPS molecule for the core oligosaccharide moieties over the simulation time (Figs. 3*B*, S1*B*) showed that RaLPS became less solvent exposed. The retention of some residual solvent exposure is expected due to the polar nature of the oligosaccharide. This behavior was seen in both 'random' and 'grid' LPS systems (Fig. 3*B*), regardless of LPS %, including in the presence of membrane proteins (Fig. S1*B*). The conformations sampled by RaLPS oligosaccharides in our simulations were distinct from conformations predicted by CHARMM-GUI and sampled in OM simulations (Figs. 3*C*, S4).

Other studies have focused on protein-free CG models comparable to those described here (40, 41); upon inclusion of 20 to 25% ReLPS in the OM leaflet, LPS aggregation occurs readily, while with 10% ReLPS, it is observed to a lesser extent than in our simulations. Notably, all such simulation studies have incorporated calcium ions, which suggests that they may not have been the primary driving force for LPS aggregation, despite their strong co-localization with RaLPS in our simulations. The extensive RaLPS aggregation observed here at 1 to 10% LPS content in the absence of PMB1 molecules may be attributed in part to the additional inclusion of the full RaLPS core oligosaccharide and thus greater potential for LPS-LPS interactions *via* the additional sugars. In a study of OM models containing mixtures of smooth LPS, RaLPS, and PLs in the outer leaflet, the presence of RaLPS and/or PLs leads to tilting and disorganization of the smooth LPS O-antigen moiety relative to simulations containing 100% smooth LPS in the outer leaflet, which is comparable to our observations with RaLPS/PL mixtures (44). Furthermore, in systems in which PL molecules are present, LPS is found to phase separately from PL molecules, with LPS saccharide-saccharide interactions playing an important role (44). It should be noted that the concentrations of LPS used in these simulations were higher than in our IM model, given the authors were simulating OM models (44). We note that another indication of the role of the LPS saccharides in aggregation comes from our recent study, in which we developed a model of ReLPS with enhanced kinetics – scaling back the saccharide interactions of LPS resulted in greater lateral mobility compared to scaling back the lipid A tail interactions (45). The physiological LPS content in the IM is thought to be ∼1% (46), and during LPS biosynthesis, RaLPS is flipped to the outer leaflet (47); therefore, it is possible that RaLPS *in vivo* may exist in a similar state to that observed in our simulations. While most of the simulations here indicate that RaLPS forms stable aggregates, the simulations with 1% LPS + LacY suggest that LPS aggregate formation may have been disrupted by the presence of membrane proteins, although it is difficult to be conclusive due to the small number of LPS molecules present in these simulations.

### Effect of membrane proteins on inner membrane dynamics

We next performed three simulation replicas of 5 μs each of IM models in the presence of one of two canonical IM proteins, either LacY (3 copies) or AmtB (1 copy). LacY uses the proton gradient to transport beta-galactoside sugars (*e.g.* lactose) from the periplasm to the cytoplasm and consists of a TM domain of 12 helices that exhibits internal two-fold symmetry along with two cytoplasmic domains. It can adopt two distinct conformations: outward-facing (pre-transport) and inward-facing (post-transport). We used the inward-facing conformation, as this conformation provides adequate space in the upper leaflet to include multiple copies of the protein and sufficient RaLPS molecules. AmtB is an ammonium/ammonia transporter that exists as a trimer, with each monomer comprising 11 TM helices with pseudo-two-fold symmetry that collectively form a channel. We selected these membrane proteins as they are abundant (LacY = 6.49–239 ppm, AmtB = 0.17–1705 ppm) (48) and have been studied extensively using MD simulations (49, 50, 51, 52, 53), allowing us to consider the effect of both monomeric and multimeric proteins.

**Figure 4:**
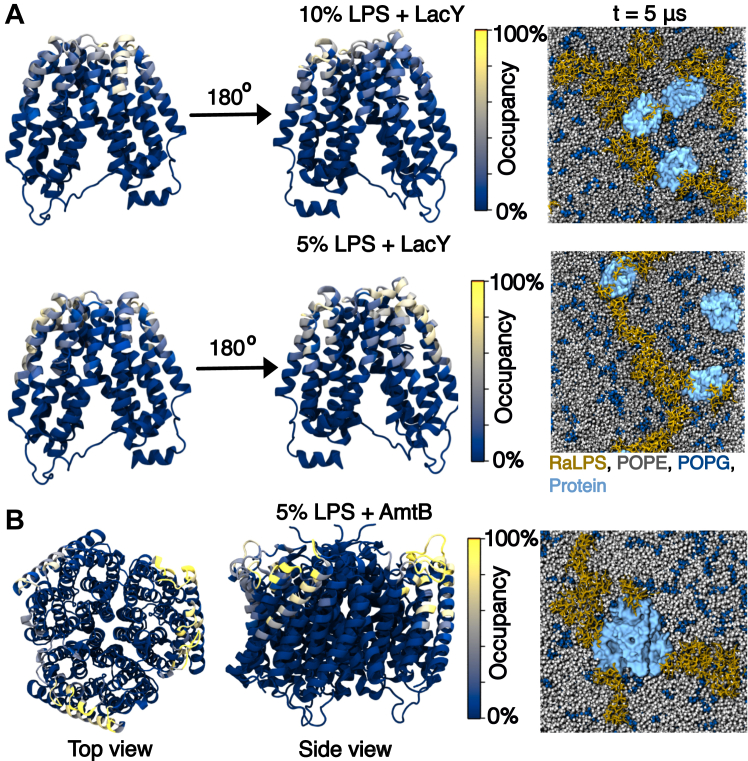
**RaLPS-protein contacts in CG simulations (in the absence of PMB1, with membrane proteins).***A* and *B*, *Leftand middle* Occupancy (% over the last microsecond of simulation) during which LPS-protein contacts (6 Å cutoff) were formed, calculated on a per-residue basis, mapped onto atomistic protein structures. Proteins are shown in cartoon format. *Right*, Representative snapshots at the end of PMB1-free simulations. Molecules are represented as RaLPS = *yellow* licorice; POPE = *gray* vdW; POPG = *darkblue* vdW; protein = *lightblue* surface. *A*, 10% LPS (top, three protein copies, n = 3), 5% LPS (bottom, three protein copies, n = 3) + LacY. Front and back views of protein are shown on the left and *middle* respectively. *B*, 5% LPS + AmtB (n = 3). Top-down and side-on views of protein are shown on the left and *middle* respectively.

To assess lipid-protein association, we first measured per-residue protein-RaLPS occupancies (% of simulation time over which a contact is formed) over the last microsecond of simulations (Fig. 4). Longer-lived (occupancy >40%) interactions were observed for both types of membrane protein. While AmtB exhibited highly specific, well-defined LPS binding sites (Fig. 4*B*), LacY had regions with occupancies at ∼65 to 70% in systems with 2 to 10% LPS. The location of these regions varied with LPS content, indicating that the protein-LPS interactions lack specificity, as demonstrated by the contact maps (Figs. 4*A*, S5*A*). Occupancies were in general lower for 1% LPS, compared to systems with higher LPS content (Fig. S5*B*). AmtB has well-defined POPG binding sites (54, 55) (at the inter-monomer interfaces in both leaflets) and is thought to have distinct cardiolipin binding sites (49, 56). Lipid binding at these sites is believed to allosterically modulate AmtB function, with POPG binding being necessary for AmtB function (54, 55, 56). LacY lipid binding sites are less well-studied: different lipids affect LacY spatial distribution and function, but this could not be linked to direct lipid-LacY binding (57). Further to this, MD simulations of LacY with Gram-negative IM models showed that upper leaflet cardiolipin can bind to LacY; however, no interactions showed occupancies >40% (49). Our findings for LPS therefore broadly agree with existing research regarding anionic lipid binding. It is worth noting that RaLPS interacted with the solvent-exposed central parts of LacY to a greater extent than those of AmtB, indicating that RaLPS adapted to the irregularly shaped periplasmic end of LacY (Figs. 4*A*, S5). Representative snapshots from the end of the simulations with 2 to 10% LPS (Figs. 4*A*, S5*A*) demonstrated the attachment of RaLPS aggregates to membrane proteins, with the aggregates extending into the bulk membrane environment. For 1% LPS, RaLPS appears to bind to membrane proteins as single molecules, unsurprisingly, given the small number of LPS molecules present (Figs. S2*B*, S5*B*). Previous research suggests that LPS exhibits distinct interaction patterns with OM proteins (58); our data indicates that this can also occur with IM proteins. In the OM, interactions between proteins and LPS lead to the formation of large supramolecular complexes (59, 60). While this is unlikely to occur in the IM given the much lower LPS content, IM LPS-protein interactions may nevertheless be important, with proteins potentially acting as LPS binding and/or aggregation sites. It should be noted that *in vivo* fluorescence measurements show that the IM may consist of both lipid-rich and protein-lipid domains (61, 62).

**Figure 5:**
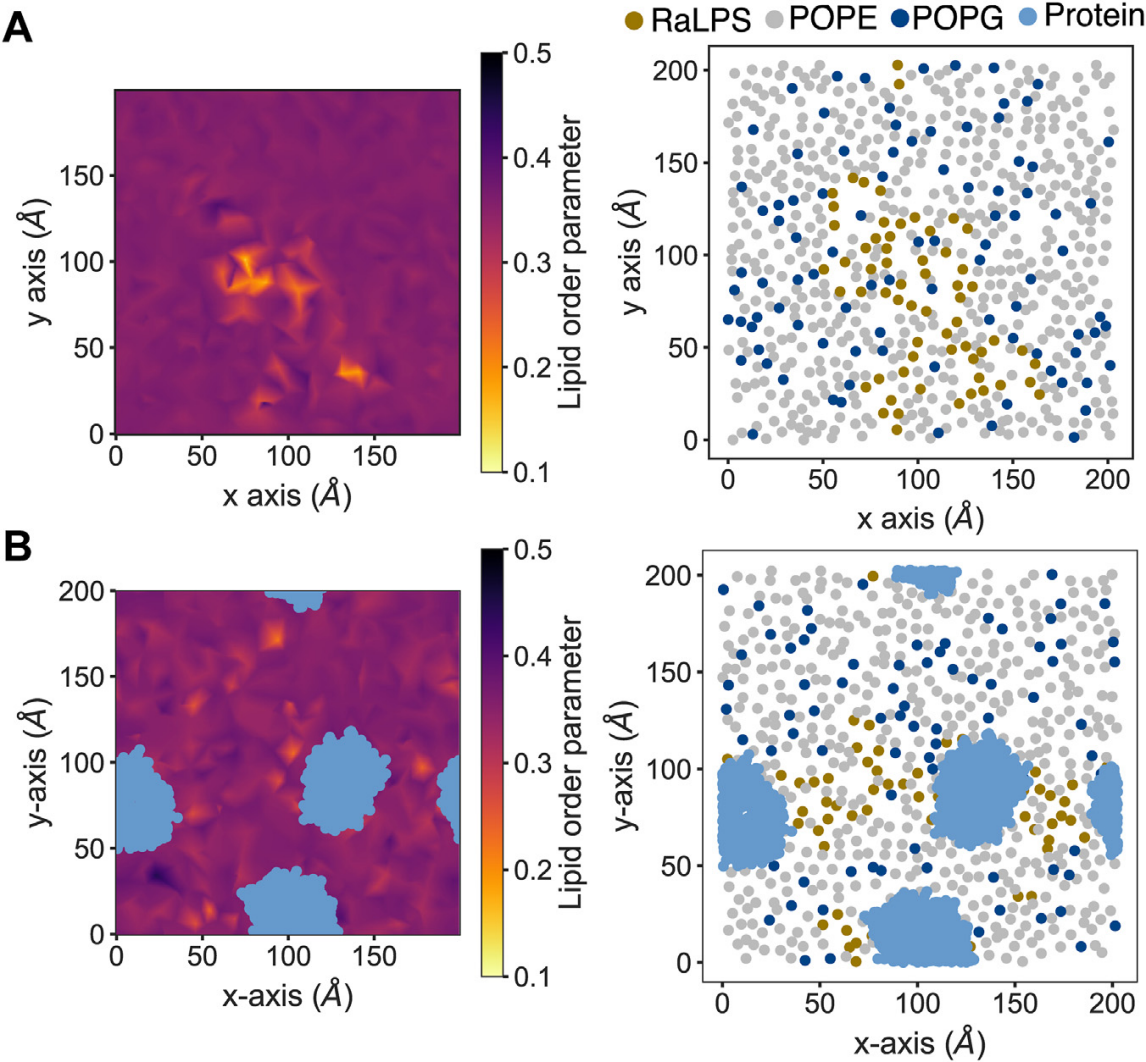
**Dynamics of lipids shown in 2D in plane of membrane (in absence of PMB1).***A*, Data for a representative 5% LPS system, without membrane proteins. *B*, Data for representative 5% LPS system + LacY. *Left*, Lipid order parameter averaged over last 0.5 μs of simulation - order parameters can vary from −0.5–1.0, with values closer to zero indicating that tails are more disordered. *Right*, Coordinates of lipid headgroup phosphates (POPE = *gray*, POPG = *darkblue*, LPS = *yellow*) and protein (where present, *lightblue*) at t = 4.75 μs.

We measured the lipid order parameters ($\left\langle S_{\text{total}}\right\rangle$), Equations 2, 3, Figure S6*A*) for lipids in the LPS-containing leaflet of representative 5 to 10% LPS systems without proteins (Figure 5*A*, Fig. S7*A*) and with LacY proteins (Figs. 5*B*, S7*B*) over the last 0.5 μs of simulations. In the absence of membrane proteins, the lipid order parameters were perturbed at locations in which LPS was clustered, displaying both more disordered ($\left\langle S_{\text{total}}\right\rangle$ < 0.30) and more ordered ($\left\langle S_{\text{total}}\right\rangle$ > 0.40) patches. This effect was in contrast to the relatively uniform values (0.35 < $\left\langle S_{\text{total}}\right\rangle$ < 0.40) observed further away from RaLPS patches and was more prominent in 10% LPS systems (Fig. S7*A*). Upon the addition of membrane proteins, it was more difficult to correlate areas of lipid disorder specifically with RaLPS locations, with disordered patches being found more frequently in the PL phase. In 5% LPS, with either LacY or AmtB (LacY results were shown here as an example), we found that more disordered regions ($S_{\text{total}}$ < 0.30) tended to be smaller in area compared to those found in 10% LPS. Overall the proteins appear to have a homogenizing effect on the membrane order parameters. We measured the area per lipid headgroup phosphate (Fig. S8), which was primarily < 75 Å^2^, with small numbers of lipids reaching > 125 Å^2^. The IM appears to be a dynamic environment, as supported by substantial variation in individual area per lipid phosphate values, and by the observation that lipid order parameters were no higher than 0.5 across all simulations, indicating that lipid tails are relatively fluid (63). In earlier work, the average CG order parameters of a mixed RaLPS/POPE (54:46) symmetric OM model were calculated, yielding values of ∼0.4 (RaLPS) and ∼0.35 (POPE) (64), broadly in agreement with the values we report here. Interestingly, previous MD studies show that IM proteins (including LacY and aquaporin Z) may disorder the membrane by induction of membrane thinning (near the membrane protein) (65) and reduction of membrane bending rigidity (66).

### LPS-mediated inner membrane insertion of PMB1

9 to 10 × PMB1 molecules were next added to each of the pre-equilibrated random LPS and protein-containing systems, and simulation replicas were performed for 3 × 20 μs. For the 5% LPS (+LacY) systems, this process was repeated following 20 μs simulation to yield extended simulations with 20 × PMB1 molecules. Unless otherwise stated, subsequent analysis was performed exclusively for the LPS-containing upper leaflet-bound PMB1 molecules, since some PMB1 molecules became bound to the inner leaflet *via* diffusion across the periodic boundary (Table S1).

**Figure 6:**
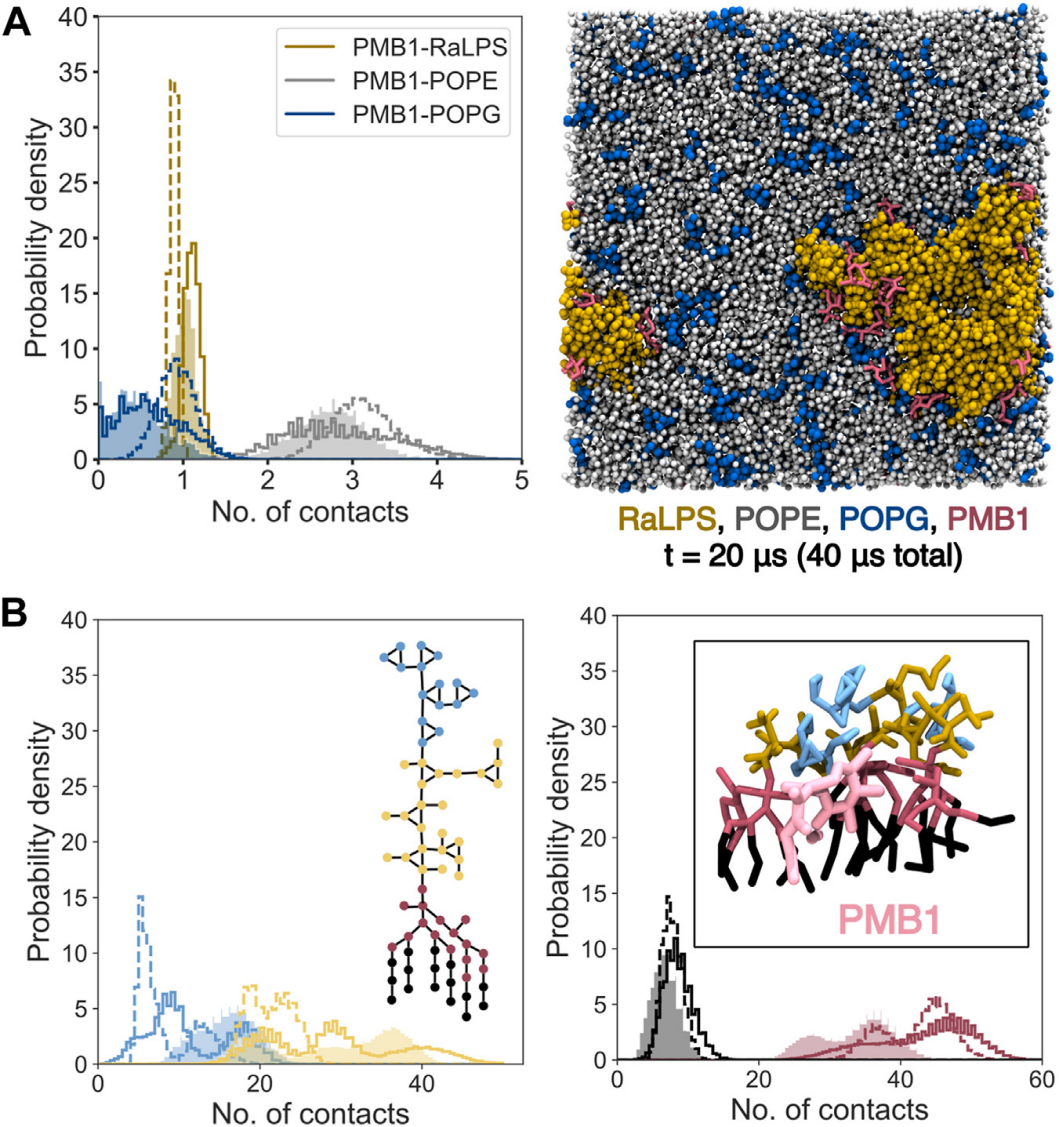
**PMB1-lipid contact distributions in CG simulations (in the absence of membrane proteins).** Distributions are shown for three systems, namely: 10% LPS (n = 3, shaded area); 5% LPS + 10 PMB1 (n = 3, solid line); and 5% LPS + 20 PMB1 (n = 3, dashed line). *A*, *Left* Distributions of PMB1-upper leaflet lipid contacts were scaled by number of upper leaflet-bound PMB1 molecules and number of beads per lipid. Contacts were distributed into 100 bins in the range of 0 to 5. Data are shown for: RaLPS (*darkyellow*, 71 beads), POPE (*gray*, 12 beads), and POPG (*darkblue*, 12 beads). *Right*, Representative snapshot of a 5% LPS + 20 PMB1 system after 20 μs simulation. Molecules are represented as RaLPS = *yellow* vdW; POPE = *gray* vdW; POPG = *darkblue* vdW; PMB1 = *maroon* licorice. *B*, Distributions of (*left*) PMB1-LPS core oligosaccharide contacts and (*right*) PMB1-LPS lipid A contacts, scaled by a number of upper leaflet-bound PMB1 molecules. Contacts were measured over the last 5 μs of simulations and distributed into 100 bins in the range 0 to 50, with distributions normalized to 100. Data are shown for RaLPS outer core oligosaccharide (*lightblue*), RaLPS inner core oligosaccharide (*lightyellow*), RaLPS lipid A headgroup + head-tail interface (*maroon*), and RaLPS lipid A tails (*black*). *Right*, inset) Representative CG snapshot at 20 μs showing the interaction of PMB1 (*lightpink* licorice) with different parts of RaLPS (licorice, same color scheme as for contact distributions).

To assess the interactions of PMB1 with the membrane, we first calculated the contacts formed with the upper leaflet over the last 5 μs for each system with LPS content of 5 to 10%, normalizing the contacts by the number of PMB1 molecules and number of lipid beads (Fig. 6, Fig. S9). PMB1 on average formed more contacts with POPE than with RaLPS (∼3:1); nonetheless, the number of PMB1-RaLPS interactions was larger than expected given the ratio of POPE:RaLPS was ≥80:10 (Figs. 6, S9). Calculation of similar distributions for different PMB1-RaLPS moieties (Figs. 6, S9) revealed that PMB1 formed most contacts with the lipid A headgroup + head/tail interface beads and with the inner core oligosaccharide. PMB1 molecules also formed contacts with the lipid A tails, albeit to a lesser extent, reflecting their capacity to insert into the bilayer. Membrane insertion behavior was assessed by measuring the center of mass (COM) of PMB1 hydrophobic moieties (D-Phe, L-Leu, fatty acyl tail) relative to the COM of upper leaflet phosphates (Fig. S10), confirming that the majority of upper leaflet-bound PMB1 molecules fully inserted into the membrane (Table S1). This was in stark contrast to comparable simulations in the presence of a Gram-negative OM model (Fig. S4), in which PMB1 predominantly interacted with the RaLPS outer core oligosaccharide and was unable to reach the lipid A layer. PMB1-LPS moiety interaction distributions were often multimodal, due to variations across replicate simulations. We did not observe a large difference in average PMB1-RaLPS contacts upon adding membrane proteins (Figs. S9, S11). However, when decreasing LPS content from 5 to 10% to 1 to 2% ranges, we saw an overall decrease in PMB1-RaLPS contacts and an increase in PMB1-PL contacts, as would perhaps be expected given the smaller quantities of RaLPS. Interestingly, in the lower LPS content systems, PMB1 molecules formed fewer contacts with the RaLPS core oligosaccharide, while contacts with the lipid A moieties of RaLPS remained relatively unchanged, indicating that PMB1 molecules interact with LPS in an inserted configuration even in the presence of only 1 to 2% LPS (Fig. S11). Finally, to confirm the accuracy of the interactions observed in the CG systems, we backmapped 10% and 5% LPS (+LacY) systems to AA resolution. PMB1-lipid (Fig. S12*A*) and PMB1-RaLPS moiety (Fig. S12*B*) contacts were well-maintained over the course of subsequent 0.5 μs AA simulations.

Overall, PMB1-RaLPS contacts were enriched in LPS-containing IM models, even at the lowest concentrations of LPS. This reflects the well-known binding affinity of PMB1 for LPS (67), and agrees with a recent simulation study of a mixed ReLPS-PL bilayer (20% ReLPS), in which PMB1-ReLPS contacts are enriched relative to PMB1-PL contacts (41). It is notable that many PMB1 molecules were able to access the lipid A headgroup and tails in our simulations, and in other simulations with mixed ReLPS-PL bilayers (40, 41), while in simulations of PMB1 with OM models (100% LPS), this occurs rarely, even when ReLPS is used (28, 29, 34, 38, 40, 41). This indicates that the presence of PLs increases the accessibility of the lipid A portion of LPS to PMB1 molecules. Interestingly, lipidomics data has revealed that the OMs of bacteria such as *A. baumannii* contain PLs in the LPS-containing outer leaflet (39), and furthermore, recent simulations indicate that PMB1 molecules may be able to promote PL movement from the lower to the upper leaflet (40). Even in the OM, small quantities of PL may enable PMB1 to bypass the LPS oligosaccharide moieties. During our simulations, we did not observe the translocation of PMB1 from the upper to the lower leaflet. This is likely to be due to the unfavorable free energy of translocation of individual PMB1 molecules through the hydrophobic region of membranes (30, 34, 40), making it a challenging event to sample over typical simulation timescales.

### Embedded PMB1 localizes at the LPS–phospholipid interface

**Figure 7:**
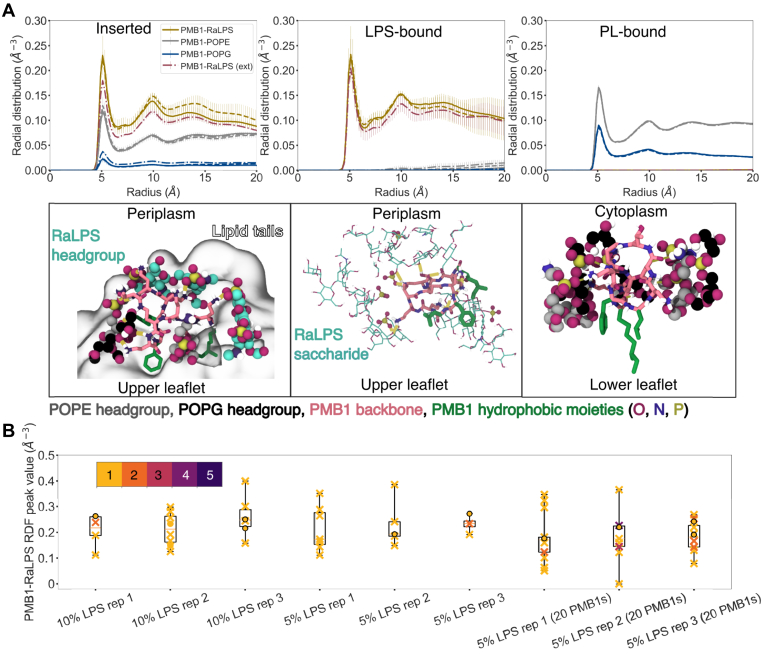
**Environment of PMB1 molecules in CG simulations (in the absence of membrane proteins).***A*, Environments of PMB1 molecules within the membrane: upper leaflet inserted (*left*), LPS-bound (*middle*), and lower-leaflet inserted (*right*). *Top*, RDFs of PMB1 molecules in each environment relative to different lipids measured over the last microsecond of simulations. RDFs were calculated from 0 to 20 Å (bin width = 0.2 Å) and were normalized by the relevant number of PMB1 molecules only. Data is shown for the following lipids: RaLPS (maroon for 5% LPS + 20 PMB1, *yellow* otherwise); POPE (*gray*); and POPG (*darkblue*). Distributions are shown for: 10% LPS + 10 PMB1 (n = 3, solid), 5% LPS + 10 PMB1 (n = 3, dashed), 5% LPS + 20 PMB1 (n = 3, dot-dash). Data was averaged over n repeats, error bars show 95% confidence intervals. *Bottom*, Snapshots of PMB1 molecules in different environments from (backmapped) AA simulations. Moieties are represented as: LPS oligosaccharide/headgroup = vdW (*left*, headgroup)/licorice (*middle*, oligosaccharide), *CPK coloring + teal* (carbons); POPE headgroup (*right*, *left*) = vdW, *CPK coloring* + *gray* (carbons); POPG headgroup (*right*, *left*) = vdW, *CPK coloring* + *black* (carbons); lipid tails = glass, transparent; PMB1 = licorice, *CPK coloring* + *pink* (carbons); PMB1 hydrophobic moieties = *green* CPK. *B*, Box and scatter plots of individual upper leaflet-bound PMB1-RaLPS RDF peak values (∼6 Å) for individual simulations, calculation details were similar to collective RDFs. Boxes span the interquartile range (IQR) of data, whiskers span minimum and maximum values. Data point multiplicity is indicated by the color bar. Inserted PMB1 molecules are depicted as crosses and surface-bound PMB1 molecules as circles.

We further probed PMB1-lipid interactions in protein-free systems with 5 to 10% LPS by measuring the collective radial distribution function (RDF) over the final microsecond of simulations for different classes of membrane-interacting PMB1 molecules (inserted, surface-bound, and lower-leaflet bound) (Fig. 7*A*). For two particles A and B, the RDF, g(r), is a measure of the probability of finding a particle of type A at radius r, from a particle of type B (Fig. S6*B*). In MD simulations, this probability corresponds to a particle density. Here the RDF was calculated for lipid particles at distances r = 0 to 20 Å from all relevant PMB1 beads (collective RDF), or at distances r = 0 to 20 Å from individual PMB1 molecule beads (individual RDFs). Surface-bound PMB1 molecules only interacted with RaLPS, while lower leaflet-bound PMB1 molecules formed interactions exclusively with POPE and POPG. By contrast, inserted PMB1 molecules exhibited PL peaks slightly lower than those of lower leaflet-bound PMB1 and a RaLPS peak comparable in size to that of surface-bound PMB1. Thus, PMB1 molecules on average either formed contacts with RaLPS and PL molecules simultaneously, or segregated deep into the LPS or PL phases. PMB1-RaLPS peak values (at radius ∼6 Å) for individual PMB1 molecules (Fig. 7*B*) across protein-free simulations were almost all non-zero, indicating that PMB1 molecules favored an interfacial environment. An example of this is depicted in Figure 7*A*, in which PMB1 is surrounded by two RaLPS lipids, two POPE lipids, and one POPG lipid, with its hydrophobic moieties inserted into the membrane tails region. In systems with only 1 to 2% LPS content and no membrane proteins, the PMB1-lipid RDF peak values were equivalent for RaLPS and POPE; individual RDF peak values indicated that this was due to a combination of PMB1 molecules in an LPS-free environment (RaLPS RDF peak value ∼ 0), and PMB1 molecules binding to LPS in a variety of interfacial environments (RaLPS RDF peak value ∼ 0.1–0.4) (Figs. S13*A*, S11*C*).

Upon backmapping to AA resolution, we observed that inserted PMB1 molecules readily formed hydrogen bonds with POPE, RaLPS, and to a lesser extent, POPG at the interface, and to RaLPS when bound to the LPS surface (Figs. S14, 7*A*), primarily acting as hydrogen bond donors on account of their 2,4-diaminobutyric acid (Dab) residues (Fig. S15*A*). At the CG level, the range of RaLPS peak values indicated that PMB1 sampled a wide range of interfacial environments, including when deeply inserted into the LPS phase (Fig. S16), whilst also interacting with PLs. It should be noted that initial PMB1 insertion did not exclusively occur at the RaLPS-PL interface, with PMB1 molecules also inserting directly into the PL phase (Fig. S17*A*) or surface-binding to LPS initially (Fig. S17*B*) and subsequently inserting into the outer monolayer on longer timescales.

Overall, these findings agree with, and expand upon previous computational and experimental work which suggests that PMB1 tends to bind to the LPS-PL interface, with a preference for interaction with LPS (31, 40, 41). However, the earlier studies (31, 40, 41) do not consider the variation in individual PMB1 behavior, nor the presence of an extensive network of hydrogen bonds at the LPS-PL interface. PMB1 insertion at this interface has previously been shown to be deeper compared to within a pure PL bilayer (40). It is noteworthy that PMB1 favored residence at the LPS-PL interface despite being able to form multiple hydrogen bonds with LPS when surface-bound (Fig. 7*B*), implying that hydrophobic interactions may also play an important role. Once localized at the interface, in the absence of proteins, PMB1 molecules remained bound (Figs. 7, S18*B*), likely due to the long-lived interactions with RaLPS, as well as restricted PMB1 mobility within the RaLPS phase, due in part to calcium cross-linking of RaLPS molecules (10). The ability of PMB1 to cross-link PL and LPS molecules in the IM is comparable to its ability to cross-link LPS molecules in simulations with Gram-negative OM models (29, 38). It is unclear whether this ability could induce self-promoted uptake of PMB1 *in vivo*. Notably, PMB1 translocation was less apparent in our AA simulations than that observed from previously reported simulations in which the *E. coli* IM model was free of trace LPS molecules (29). In our simulations, the extensive network of hydrogen bonds and salt bridges formed by PMB1 at the LPS-PL interface (and corresponding polar and electrostatic interactions present in the CG systems) likely contributed to the strong co-localization of PMB1 molecules at the interface and presented a significant barrier to sampling even partial PMB1 translocation over currently accessible timescales.

### PMB1 interaction with membrane proteins and RaLPS

**Figure 8:**
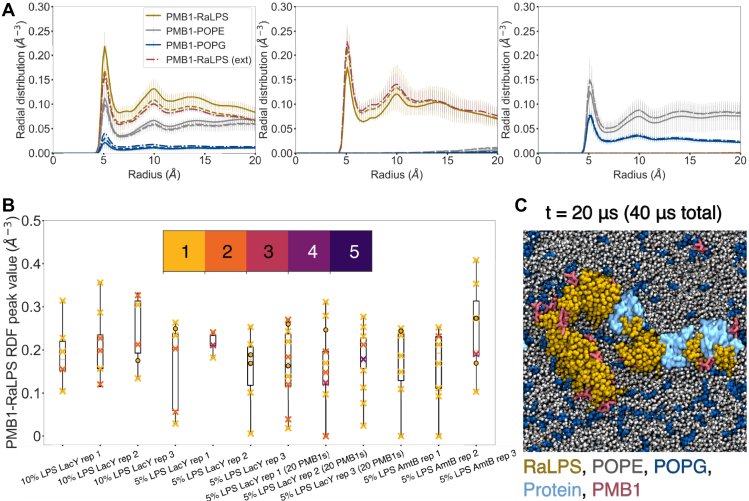
**Environment of PMB1 molecules in CG simulations (in the presence of LacY).***A*, environments of PMB1 molecules within the membrane: upper membrane inserted (*left*), LPS-bound (*middle*), and lower-leaflet inserted (*right*). RDFs of PMB1 molecules in each environment relative to different lipids were measured over the last microsecond of simulations. RDFs were calculated for radii from 0 to 20 Å (bin width = 0.2 Å) and were normalized by a relevant number of PMB1 molecules only. Systems shown include: 10% LPS + LacY + 10 PMB1 (n = 3, solid); 5% LPS + LacY + 10 PMB1 (n = 3, dashed); and 5% LPS + LacY + 20 PMB1 (n = 3, dot-dash). Data are shown for the following lipids: RaLPS (*maroon* for 5% LPS + 20 PMB1, *yellow* otherwise), POPE (*gray*), and POPG (*darkblue*). Data was averaged over n repeats, error bars = 95% confidence intervals. *B*, box and scatter plots of individual upper leaflet bound PMB1-RaLPS RDF peak values (∼6 Å) for individual simulations, calculation details similar to collective RDFs. The boxes span the interquartile range (IQR) of the data, while the whiskers extend to the minimum and maximum values. Data point multiplicity is indicated by the color bar. Inserted PMB1 molecules are depicted as crosses and surface-bound PMB1 molecules as circles. *C*, PMB1 molecules at the end of 20 μs CG simulation (5% LPS + LacY + 20 PMB1). Molecules are represented as RaLPS = *yellow* licorice; POPE = *gray* vdW; POPG = *darkblue* vdW; PMB1 = *pink* licorice; proteins = *lightblue* surface.

**Figure 9:**
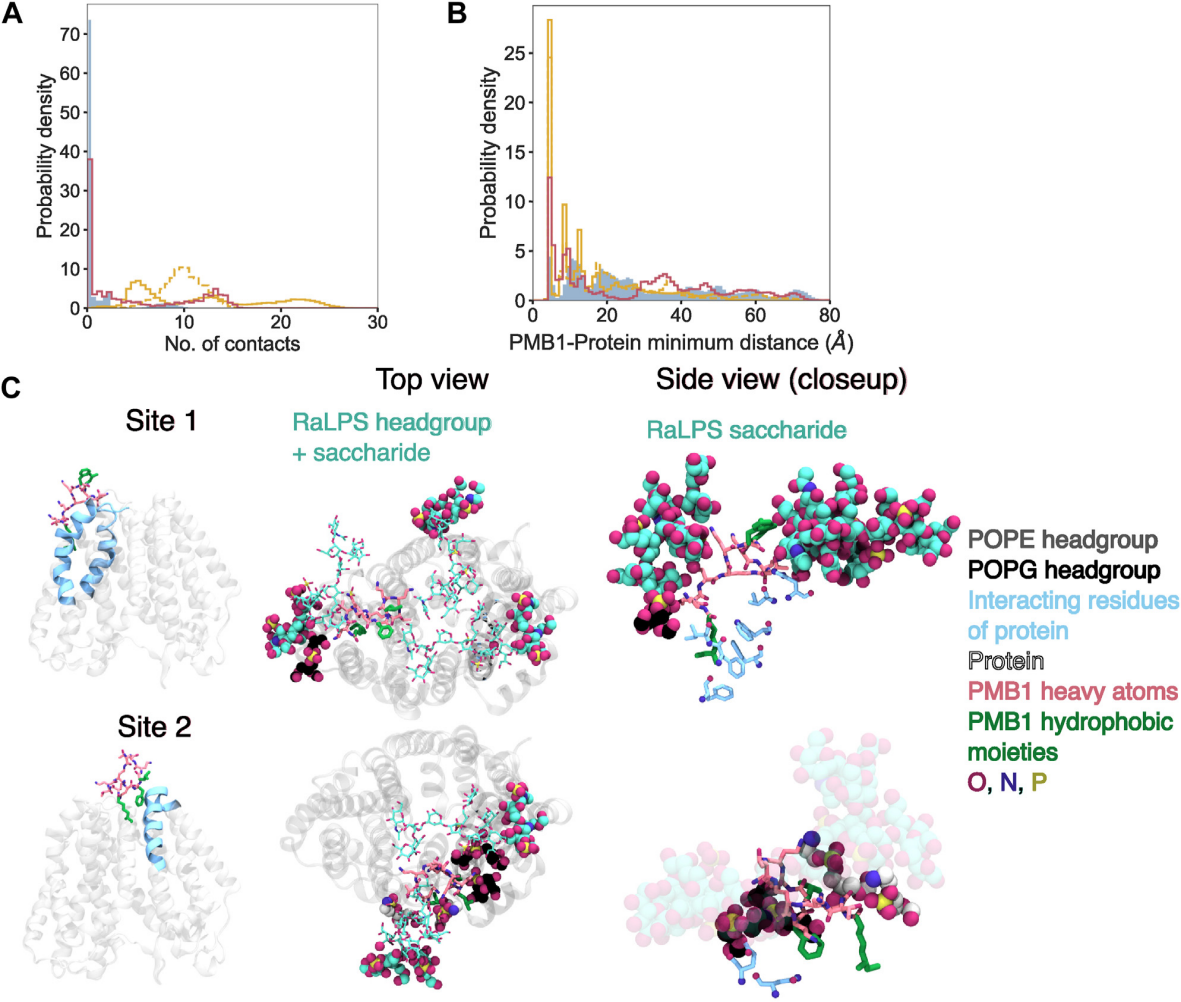
**Nature of PMB1-protein contacts in CG simulations.***A*, Distribution of PMB1-protein contacts calculated over last 5 μs of simulation scaled by number of upper leaflet-bound PMB1 molecules. Contacts were distributed into 100 bins in the range 0–50. *B*, Distribution of (individual) upper leaflet-bound PMB1-protein distances calculated over last 5 μs of simulations. Distances were distributed into 100 bins in the range 0 to 100 Å. Systems shown include: 10% LPS + LacY + 10 PMB1 (n = 3, shaded area, *blue*), 5% LPS + LacY + 10 PMB1 (n = 3, solid line, *yellow*), 5% LPS + LacY + 20 PMB1 (n = 3, dashed line, *yellow*), 5% LPS + AmtB + 10 PMB1 (n = 3, solid line, *maroon*). Distributions in (*A* and *B*) are normalized to 100. *C*, Snapshots from end of backmapped 0.5 μs AA simulation (5% LPS + LacY + 20 PMB1) showing interaction of PMB1 molecules at two interaction sites of the same LacY protein. Location of PMB1 interaction site (*left*), top view (*middle*), side view (*right*). Molecules are represented as: POPE headgroup = vdW, *CPK color scheme* + *gray* (carbons); POPG headgroup = vdW, *CPK color scheme* + *black* (carbons); RaLPS headgroup (*middle*) = vdW, *CPK color scheme* + *cyan* (carbons); RaLPS saccharide = licorice (*middle*), vdW (*right*), *CPK color scheme* + *cyan* (carbons); PMB1 = *CPK color scheme* + *pink* (carbons); PMB1 hydrophobic moieties = *green*; protein = transparent cartoon (*left*, *middle*); PMB1-interacting protein residues = *lightblue* cartoon (*left*); licorice (*right*), *CPK color scheme* + *lightblue* (carbons).

The membrane protein-containing systems were analyzed using similar approaches to the aforementioned protein-free systems (Fig. 8, Figs. S13, S19). The collective RDFs with 5 to 10% LPS + LacY/AmtB were broadly similar to those in the absence of membrane proteins, while with 1 to 2% LPS systems, RaLPS and POPE collective RDF peaks were slightly reduced in the presence of membrane proteins (Figure 8, Figs. S13*B*, S19). Inspection of individual PMB1-RaLPS peaks (Fig. 8*B*) revealed that across all 5% LPS + LacY/AmtB systems, several PMB1 molecules did not interact with RaLPS over the last microsecond of the simulations. This was in contrast with the protein-free systems, where just one PMB1 molecule across all simulations existed in an LPS-free environment (Fig. 7*B*). Interestingly, for systems with 10% LPS, PMB1 molecules were generally not found in a RaLPS-free environment in the presence or absence of membrane proteins (Figs. 7*B*, 8*B*), whereas for systems with 1 to 2% LPS, PMB1 molecules were more often found in a RaLPS-free environment regardless of the presence of membrane proteins (Fig. S13). In a low LPS environment, individual RaLPS peak values were consistently lower (≤ 0.3) in the presence of membrane proteins (Fig. S13*B*). Furthermore, for 5% LPS membrane protein-containing systems, calculation of PMB1-protein collective and individual RDFs revealed that ∼1 to 6 PMB1 molecules interacted with proteins in most simulations (Fig. S20*A*); this behavior was not seen at 10% LPS. Snapshots for 5% LPS-containing systems indicated that some PMB1 molecules were able to interact with proteins, PLs, and RaLPS (saccharide moieties) concurrently (Figs. 8*C*, 9*C*), while other PMB1 molecules interacted with membrane proteins in the absence of LPS (Fig. 8*C*). Collective RDFs and snapshots for 1 to 2% LPS membrane protein-containing systems also showed the presence of PMB1-protein colocalization (Figs. S20*A*, S11*C*).

We calculated the distribution of upper leaflet PMB1-protein contacts in systems with 5 to 10% LPS over the last 5 μs of simulations, scaling the contacts by the number of upper leaflet-bound PMB1 molecules (Figs. 9*A*, S21*A*). For systems containing 10% LPS + LacY and 5% LPS + AmtB, the first histogram bin (corresponding to 0–0.5 contacts per PMB1) had the highest probability density (ranging from 30-80%), indicating that on average, PMB1 molecules frequently did not contact membrane proteins in these systems. For 5% LPS + LacY systems this did not hold true; instead, PMB1-protein contacts were on average ubiquitous throughout the simulations, although low in number (<30 per PMB1). In accordance with this, the probability of PMB1 molecules being found in the vicinity of membrane proteins (∼6 Å) was lower in 10% LPS systems than in 5% LPS systems (Fig. 9*B*). At lower LPS content, the number of PMB1-protein contacts was increased, along with PMB1-protein peak values, to a lesser extent (Figs. S20, S21). PMB1-protein survival times encompassed a wide range, with many PMB1 molecules adopting both short (≤ 5 μs) and long (≥ 15 μs) times (Fig. S21).

Based on the calculation of PMB1-protein per-residue occupancies (Figs. S20*B*, S21*C*), LacY proteins had specific patches that showed frequent (∼40% occupancy) interactions with PMB1 molecules for the 1% and 5% LPS systems, while other residues had lower occupancies, indicating that they formed less specific interactions with PMB1 (Fig. S20*B*). Interestingly, in the 10% and 2% LPS systems, interactions were not formed with this patch, which instead appeared to form interactions with RaLPS (Figure S20*B*, Figure S21*B*, Figs. S5, 4). Snapshots indicated that PMB1 interactions with the LacY alpha-helices were hydrophobic in nature, while binding to the LacY loop regions may have involved electrostatic interactions (Fig. 9*C*). In comparison to PMB1-RaLPS interactions, PMB1-protein interactions (in 5–10% LPS systems) were lower in occupancy (Figs. 4, S20); for individual PMB1 molecules in 5 to 10% LPS systems, maximum protein residue interaction survival times tended to be low (<1 μs), but some were comparable to PMB1-RaLPS survival times (>5 μs) (Fig. S18*A*). Meanwhile, RaLPS-bound survival times for PMB1 molecules were frequently longer-lived (≥15 μs), often lasting throughout the simulation (Fig. S18*B*), but in the presence of membrane proteins, the number of PMB1 molecules with RaLPS interaction times ≤ 5 μs was slightly increased in systems with 5% LPS (Fig. S18*B*). We note that LacY proteins remained relatively stable in terms of RMSD over the course of backmapped AA simulations (Fig. S22*B*), with the largest RMSF values mostly occurring within sections of the protein located at the inner leaflet (Fig. S22*C*), giving some validation of the CG protein model. At the AA level, PMB1-protein interactions were stable (Fig. S12*C*) and were comprised of hydrophobic interactions rather than hydrogen bonds (Figs. S12*C*, S14).

Collectively, our results showed that a small number of PMB1 molecules tended to form interactions with the two IM proteins studied here, with PMB1-protein contacts being enhanced at lower LPS concentrations (Figs. 9*A*, S21*A*). For 1 to 5% LPS + LacY systems, long-lived PMB1-protein interactions were seen alongside short-lived interactions (Figure 9, Figure S18*A*, Figs. S20, S21). In all cases, the fact that PMB1-protein occupancies never exceeded 50% indicates that PMB1 interactions with LacY and AmtB lack high specificity, while the occupancy patterns showed that protein-LPS interactions could modulate protein-PMB1 interactions (Figs. S20*B*, S21*C*). The presence of the IM proteins was shown to have a modulatory effect on PMB1-LPS interactions, which depended upon LPS content (Figure 7*B*, Figure 8*B*, Figs. S18*B*, S13); the statistical significance of these changes is difficult to assess given that they occurred rarely, but it is worth noting the indirect influence arising from the presence of IM proteins, possibly due to their perturbing effect on the membrane dynamics (Figure 5, Figs. S7, S8). The physiological impact of membrane proteins on PMB1-LPS interactions is currently unclear but may be important *in vivo* since the Gram-negative IM contains many membrane proteins that could affect how PMB1 bypasses the membrane (61, 62, 65, 66, 68). Indeed, the strong impact of crowding on PMB1 interactions in the periplasm has already been reported in a previous study (69).

### Equilibrium conformations of upper leaflet-bound PMB1 molecules

**Figure 10:**
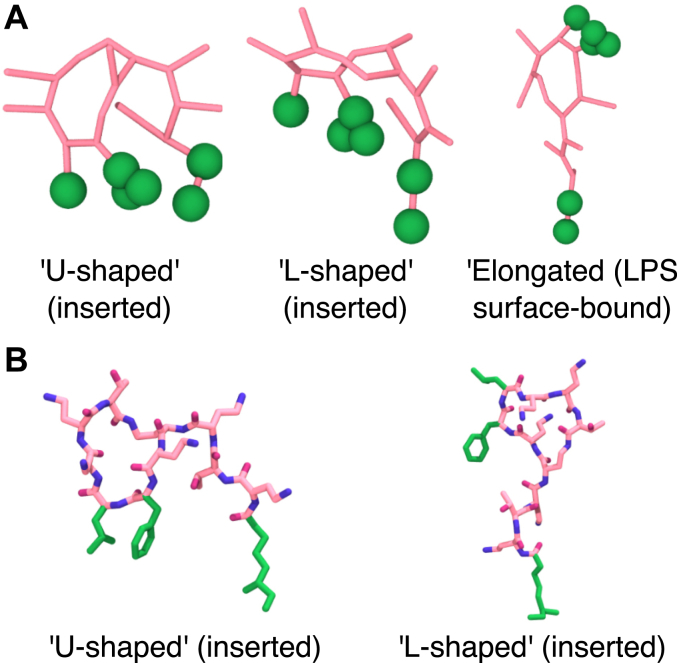
**Representative PMB1 conformations sampled across CG and AA simulations.***A*, Representative inserted and LPS surface-bound PMB1 conformations from CG simulations. *B*, Representative inserted PMB1 conformations from AA simulations. PMB1 = licorice, *pink* (A), *CPK colouring* + *pink* (carbons) (B); PMB1 hydrophobic moieties = vdW (A), licorice (B), *green*. In both panels PMB1 molecules are oriented with the hydrophobic tail parallel to the vertical axis.

To investigate PMB1 conformational changes at equilibrium, we concatenated the final microsecond of trajectories for individual inserted PMB1 molecules across multiple repeat simulations of a given CG system, structurally aligned these “pseudo-trajectories,” and performed clustering (cutoffs in Table S2). Regardless of the presence of membrane proteins, inserted PMB1 conformations favored ‘folded over’ conformations, whereby their hydrophobic residues and tail were on the same side of the molecule (Figure 10, Fig. S23). Within the inserted cluster centers, two extremes in the degree of PMB1 folding could be identified, between which most relevant inserted cluster centers existed: (1) a ‘U-shaped’ conformation: the PMB1 ring and PMB1 tail were parallel, with hydrophobic moieties inserted to a similar degree; 2) an 'L-shaped conformation': the PMB1 ring and PMB1 tail were perpendicular, with the tail inserted deeper than other hydrophobic moieties (Figs. 10*A*, S23). Most inserted cluster centers were closer to the ‘U-shaped’ than the “L-shaped” conformation (Fig. S23). It should be noted however that the conformational shape did not correspond exactly to the degree of insertion of hydrophobic moieties, and that in Figure S23 and Figure S24 we only displayed the orientation of cluster centers with respect to the membrane normal: other cluster members may have adopted a similar conformation but inserted into the membrane in a different orientation. Surface-bound PMB1 molecules also showed ‘folded over’ conformations, alongside elongated conformations, with the hydrophobic moieties facing away from the membrane center in most cases (Figs. 10*A*, S24).

To validate our CG results, we ran a similar conformational analysis on inserted PMB1 molecules in AA simulations (0.1–0.5 μs, Table S3, Fig. S25). In agreement with our CG simulations, we could not correlate changes in the PMB1 central cluster structure with the presence of membrane proteins (Fig. S25). The highest occupancy clusters in most AA simulations appeared to be closer to the “U-shaped” conformation than the ‘L-shaped’ conformation(Figure 10, Figure S25). Cluster centers resembling the “L-shaped” conformation were also observed; however, the orientation of insertion of these conformations varied greatly, with both shallow (Figs. S25, 5% LPS ext, cluster 1) and deep (Figs. S25, 5% LPS LacY ext, cluster 2) insertion modes observed (Figs. 10, S25). The inclusion of atomistic detail revealed subtle differences in the local conformation of the PMB1 heptapeptide ring, linear portion, and their positioning relative to one another within the clusters. Upon backmapping from CG to AA resolution, the PMB1 molecules may have undergone some conformational readjustment, as highlighted by the fact that in some cluster centers, the hydrophobic moieties were not on the same side (Fig. S25).

The 'U-shaped' inserted PMB1 conformations identified in CG and AA simulations were similar to conformations characterized in previous experimental (70) and simulation (29, 30) studies. It is notable that across systems, we did not see a large range of conformations, despite the wide variation in interfacial environments sampled by inserted PMB1 molecules; even LPS surface-bound PMB1 molecules exhibited folded conformations similar to those observed for inserted PMB1 molecules. This may have been due in part to the presence of intramolecular hydrogen bond(s) in PMB1 molecules (Fig. S15*B*), which may have inhibited rapid conformational transitions. Additionally, PMB1 molecules could have had similar conformations but different orientations within the membrane. Comparison between conformations sampled in LPS surface-bound and inserted PMB1 states indicates that for PMB1 to transition between these two states, it may have either 'rolled' and changed orientation, while remaining folded, or switched from being elongated to folded.

### PMB1 translocation *via* electroporation at protein–phospholipid interface

To accelerate the translocation of PMB1 molecules across the entire lipid bilayer, simulations of electroporation induced *via* a TM charge imbalance in a double-bilayer system were next performed for 5% LPS + LacY systems. This may also be of physiological relevance, given the TM potential that exists across the Gram-negative IM (71). Thus, a double-bilayer 5% LPS + LacY system was constructed, to which 20 PMB1 molecules were added within the inner compartment (Fig. 2*C*). We then introduced a charge imbalance between the two isolated compartments, to induce electroporation, with TM charge imbalances ranging from +138e to +142e (Table 2).

**Table 2:** Movement of PMB1 molecules and PLs across the electroporated bilayer

Transmembrane charge/e	Repeat	No. of PMB1 permeations	No. of POPE flip-flops	No. of POPG flip- flops	Open pore time window/μs
+138	1	1	40	38	0.133
	2	1	40	33	0.136
	3	1	45	29	0.112
+140	1	0	1	0	0
	2	6	5	17	0.022
	3	1	40	33	0.208
+142	1	0	45	41	0.186
	2	3	23	24	0.091
	3	3	37	26	0.100

Number of PMB1 molecules passing from inner to outer compartment of double bilayer, number of completed POPE and POPG flip-flops by end of simulation across the electroporated bilayer, time period the pore was open for.

**Figure 11:**
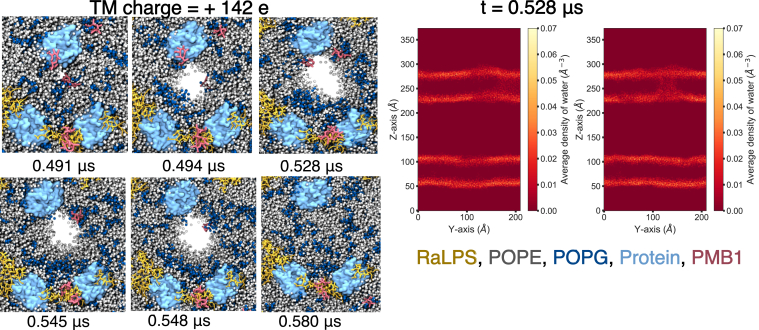
**CG electroporation of a double bilayer (TM charge difference = +142 e (inner compartment vs outer compartment).***Left*, Snapshots showing formation and closure of a pore during simulation, shown in top-down view. Molecules are represented as: RaLPS = licorice, *yellow*; POPE = vdW, *silver*; POPG = vdW, *dark blue*; PMB1 = licorice, *pink*; protein = surface, *light blue*. *Right*, Cross-section of water density within 10 Å of lipid phosphates in xz (*left*) and yz (*right*) planes.

**Figure 12:**
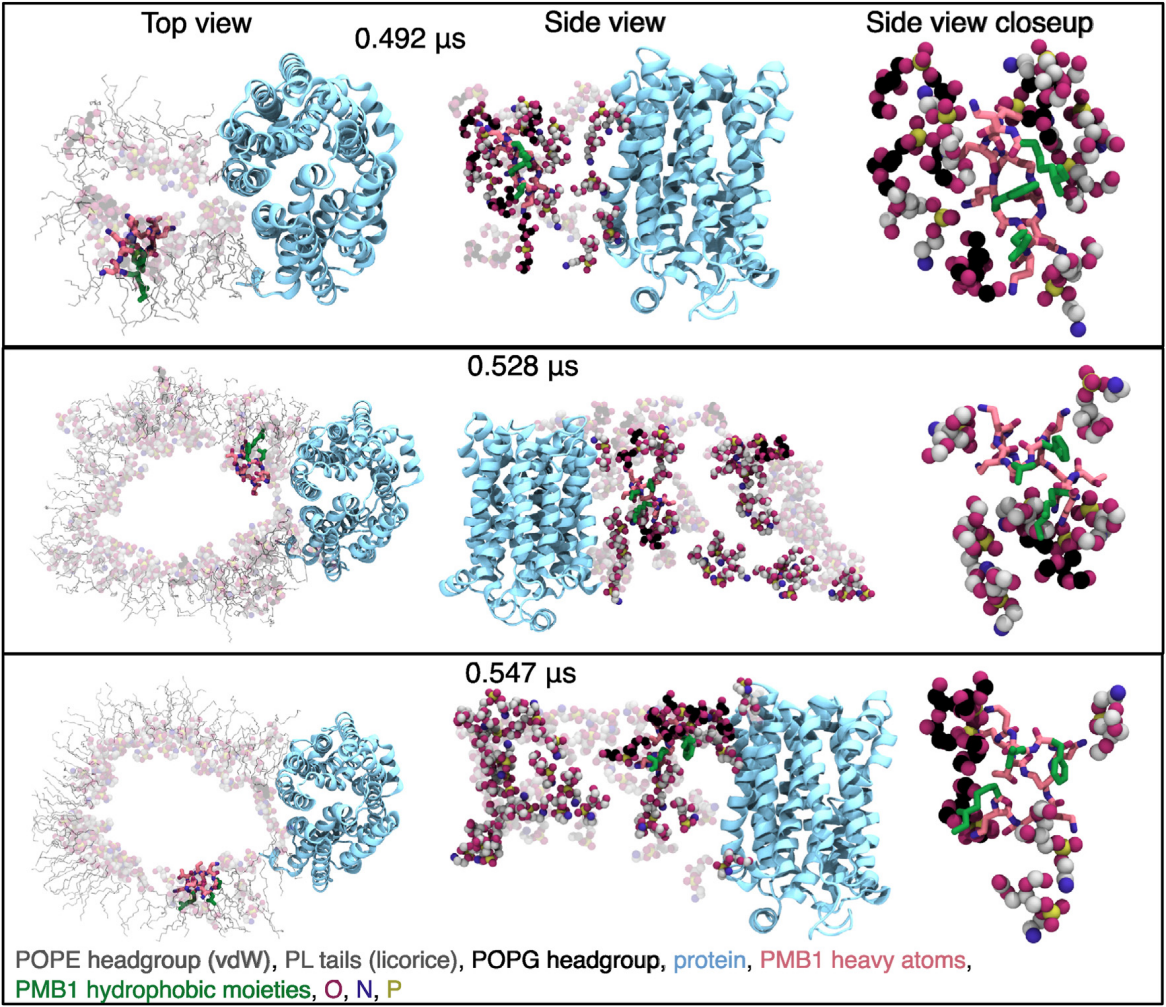
**Molecular details of CG electroporation of a double bilayer (TM charge difference = +142 e (inner compartment vs outer compartment).** Snapshots showing the passage of 3 separate PMB1 molecules through the pore formed during electroporation were backmapped from CG to AA resolution, water molecules were hidden for clarity. Top-down view (*left*), side view (*middle*), side view closeup (*right*). Molecules are represented as POPE headgroup = vdW, *CPK coloring* + *gray* (carbons); POPG headgroup = vdW, *CPK coloring* + *black* (*carbons*); POPE/POPG tails = licorice, translucent *gray*; PMB1 = licorice, CPK coloring + *pink* (carbons); PMB1 hydrophobic moieties = *green* CPK; protein = cartoon, *lightblue*. All lipid headgroups are translucent in the top-down view (*left*), and background lipid headgroups are translucent in the side view (middle).

Across simulations, the time required for electroporation did not generally correlate with the TM charge (Fig. S26), with pores remaining open for ∼20 to 200 ns in most cases, highlighting the stochastic nature of the electroporation process (Table 2). The similar behavior seen at all field strengths is likely due to similarity in the initial TM voltages (Fig. S27). In all cases, electroporation was initiated at the protein–PL interface, before growing outwards into the bulk membrane (Fig. 11, Figs. S28–S30), although sometimes, the pore was shown to migrate into the PL bulk phase over time (Fig. S28, replica 2). In some cases, these pores reached as far as the RaLPS in the membrane, although they did not grow beyond this, with RaLPS apparently acting as a barrier. Water-filled pores primarily adopted cylindrical morphologies (Figure 11, Figs. S28–S30), with the capacity for the porated leaflet to bend significantly in some cases. Typically, at least one PMB1 molecule traversed these water-filled pores, and this could occur with up to six PMB1 molecules (Table 2), with a weak correlation observed between field strength and the number of permeating PMB1 molecules. Across all simulations, pores were lined by PL headgroups, which facilitated the translocation of PMB1 by interacting with its Dab residues (Fig. 12).

By measuring the number of POPE and POPG molecules in each leaflet of the porated membrane over time (Fig. S26), we could identify lipid flip-flop events, which occurred exclusively within the porated bilayer. In all cases, POPG lipids flipped from the LPS-free leaflet to the LPS-containing leaflet, thereby partially neutralizing the excess positive charge within the inner compartment, while a similar number of POPE lipids flipped from the LPS-containing leaflet to the LPS-free leaflet, presumably to minimize any surface tension induced by POPG lipid flip-flops. It should be noted that the number of POPG flip-flops correlated with the length of time for which the pore was open (Table 2) The redistribution of charge caused by PMB1 permeation and POPG flip-flop caused the TM electric field strength to drop following pore closure (Fig. S26). There appears to be a relationship between the number of PMB1 molecules passing through the pore, the length of time the pore is open, and the number of PL flip-flops observed, although it is difficult to define a causal relationship between these factors.

It is noteworthy that electroporation never occurred at the RaLPS-PL interface, despite this interface being predicted to be a less stable part of the membrane for permeation (31, 40). Additionally, *in vitro* experimental studies of mixed LPS/PL bilayers (without membrane proteins) indicate that PMB, ReLPS, and PG lipids (to a lesser extent) collectively facilitate electroporation at a TM voltage of 50 mV (close to IM potential) (32). In our simulations, the most electroporation-susceptible part of the membrane was at the protein-PL interface, from which membrane pores grew. The lack of involvement of RaLPS in any membrane pores is in agreement with a previous computational electroporation study with LPS-containing membranes (72). Overall, this is a further indication of the 'destabilizing' role proteins may have within the IM, supporting our findings from the previous section.

## Conclusion

We present the first multiscale (CG and AA) simulation study of PMB1 interacting with IM models containing trace quantities of LPS plus membrane proteins. The serial multiscale approach we have adopted enables us to explore, at atomistic resolution, the details of the interactions between antimicrobials, lipids, and membrane proteins, after the systems have first been simulated at the CG level, to ensure that membrane proteins are correctly orientated and that any inaccuracies in orientation in the initial setup have been alleviated (73, 74, 75, 76). Our results predict that small quantities of RaLPS present in the IM are likely to exist in an aggregated state, binding to membrane proteins, although LPS aggregation may be disturbed by the presence of membrane proteins at physiological LPS concentrations. The interactions of PMB1 with RaLPS are altered in the IM, compared to the OM, with PMB1 primarily inserting at the LPS-PL interface, sampling a variety of interfacial environments and adopting defined folded states, which typically lie between the extremes of ‘U-shaped’ and ‘L-shaped’ conformations. By contrast, in our RaLPS-containing OM simulations, PMB1 binds to the RaLPS core oligosaccharide and does not readily insert into the lipid A tail region over a multi-microsecond timescale. In the IM, PMB1 cross-links lipids at the interface *via* numerous hydrogen bonds, which may inhibit its transverse motion; further work relating the hydrogen bonding network to the energy barrier previously observed for PMB1 translocation at the LPS-PL interface (40), would be of value. Adding proteins perturbs membrane lipid order (at 5–10% LPS content) and appears to slightly weaken PMB1-LPS interactions in a manner that depends on the LPS content present (1–5%). PMB1 is shown to form low specificity but long-lived interactions with the membrane proteins studied here, with LPS-protein contacts modulating PMB1-protein contacts. The subtle impact of membrane proteins and the interplay between proteins, LPS and PMB1 molecules highlights the importance of including proteins in membrane models, and of generally simulating molecular models as close in composition to microbiology experiments as is feasible (77). Finally, electroporation simulations *via* charge imbalance are used to characterize the translocation of PMB1 through the IM, and interestingly, they are not observed to pass through the RaLPS-PL interface, as suggested by Fu. *et al.* (40). Instead, large, transient membrane pores are formed at the protein-PL interface, indicating that this is the weakest part of the membrane for putative PMB1 passage. The mechanistic insights from our simulations may facilitate the rational design of new antibiotics based on the polymyxin scaffold.

## Experimental procedures

### Coarse-grained simulation system setup

#### Single bilayer systems

CG IM model bilayers (200 × 200 Å) were built using CHARMM-GUI Martini Maker (78, 79, 80, 81) with the following compositions (based on IM models used in previous simulations) (28, 29, 66, 82, 83, 84, 85, 86, 87, 88):

• Outer leaflet: 80% POPE, 10 to 19% POPG, 1 to 10% RaLPS (Fig. 1*A*).
• Inner leaflet: 80% POPE, 20% POPG.

IM systems without membrane proteins were also constructed with RaLPS molecules initially spaced in an ordered grid arrangement (referred to here as 'grid LPS systems') which differed to systems containing pseudo-random lipid placement obtained *via* CHARMM-GUI (referred to as 'random LPS systems') (Fig. 2*A*). CG LPS models, including the RaLPS model used here, have been developed by the Khalid group (44, 64), and have been used in a number of studies (34, 58, 59, 66, 89, 90).

Systems were built either without membrane proteins, with three copies of LacY (42) or with one copy of trimeric AmtB (43), using the replacement method (91) (Fig. 2*B*). For membrane protein-containing systems, protein orientations relative to the bilayer normal were obtained from the OPM server (92) and converted to CG resolution using the Martini Maker module of CHARMM-GUI. The lateral location and orientation of proteins in the bilayers were assigned at random. The secondary and tertiary structures of proteins were maintained with the ElNeDyn elastic network strategy (93). All residues were assigned default protonation states at physiological pH, except for Glu325 in LacY, which was modeled in its protonated form, as in previous work (42, 94).

A previously reported and validated CG model of PMB1 was used (Fig. 1*B*) (34, 95). Initially, 10 PMB1 molecules were added to the system at random locations, albeit positioned slightly closer to the LPS-containing leaflet, with the PMB1 COM ∼30 to 50 Å away from the COM of the upper leaflet lipid headgroup phosphate beads in the z-direction (parallel to the bilayer normal). Multiple simulation replicas (generated from different initial system configurations) were generated for each system to enhance conformational sampling. For one replica per system, 9 × PMB1 molecules were added in a regular grid-like distribution to rule out the potential biasing of molecular interactions due to the initial PMB1 placement. In an additional set of simulations, another 10 to 11 copies of PMB1 were similarly added to selected systems (Table 1), following prior 20 μs simulations with 9 to 10 × PMB1 molecules, to give a total of 20 × PMB1 molecules, to replicate the effect of gradually increasing PMB1 concentrations.

All CG system components were treated using parameters from the Martini 2.2 force field (96). All systems were solvated with a 40 Å layer of Martini non-polarizable water on either side of the membrane. RaLPS phosphate groups were neutralized with Ca^2+^ ions (placed near to LPS phosphate beads) and remaining system charges were neutralized with 0.15 M NaCl.

#### Electroporation in double bilayer systems

Double bilayer systems were built by taking final snapshots from simulations of CG single bilayer systems (5% LPS + LacY) and duplicating and rotating one bilayer by 180° in the x-axis (*i.e.* around an axis parallel to the bilayer plane), yielding two bilayers with their upper leaflets facing each other, separated by a ∼130 Å space (minimum distance) (Fig. 2). Solvent and ions (excluding Ca^2+^ ions) were removed prior to building the double bilayer; the system was subsequently resolvated with Martini polarizable water (97), and the two compartments were neutralized with 0.15 M NaCl. The polarizable water model was used in these systems to enable the accurate study of electroporation effects. The system was then simulated for 1 μs, after which PMB1 molecules were added, followed by ions to introduce various charge imbalances between the two system compartments (Fig. 2), enabling observation of electroporation, while maintaining charge neutrality of the system as a whole.

### All-atom simulation system setup

Final snapshots of systems of interest (10% LPS (+LacY) + 10 PMB1, 5% LPS (+LacY) + 20 PMB1) were backmapped to AA resolution using the CG2AT2 program (98). The CHARMM36m force field (2019 version) was used for proteins, lipids, and ions (99), while previously validated parameters from CGenFF (version 4.0) (100) were used for PMB1 (69).

### Simulation parameters and protocols

#### Coarse-grained simulation parameters

Initially, single bilayer systems were minimized and equilibrated according to the CHARMM-GUI Martini Maker protocol (78, 80) and then simulated for 5 μs (20 fs timestep). For double bilayer systems: i) minimization used ≤ 50,000 steps of steepest descent, followed by ii) NPT equilibration for 5 ns (10 fs timestep) and iii) NPT production simulations of 1 μs (20 fs timestep). Upon addition of PMB1 molecules or ions to these systems, we carried out a short minimization (≤ 5000 steps of steepest descent) and NPT equilibration (2.5–5 ns (10–20 fs timestep, 1000 kJ/mol nm^2^ position restraints applied to PMB1 beads). We then ran production simulations of 20 μs (20 fs timestep) for single bilayer systems or 1 μs for double bilayer systems with the Computational Electrophysiology protocol (101) applied for double bilayer systems to maintain charge imbalances of +138, +140, +142 e (swap direction = z-axis, swap frequency = every 100 steps).

In all CG systems temperature was maintained at 310 K with the velocity-rescale thermostat (102, 103) (τ = 1 ps), where separate temperature coupling groups included protein, solvent (*i.e.* water, ions, and PMB1), and lipids. The pressure was maintained semi-isotropically at 1 bar with either the Berendsen barostat (103) (equilibration, τ = 5 ps, compressibility = 3 × 10^-4^ bar^-1^) or Parrinello-Rahman barostat (104) (production, τ = 12 ps, compressibility = 3 × 10^-4^ bar^-1^). Electrostatics were treated using the reaction-field method for single-bilayer systems ($\varepsilon_{r}$ = 15, cutoff = 11 Å) or the particle-mesh Ewald (PME) method (105, 106) for double bilayer systems ($\varepsilon_{r}$ = 2.5, cutoff = 11 Å) and van der Waals interactions were treated with a potential shift + Verlet cutoff scheme (cutoff = 11 Å). Simulations were performed with the leapfrog integrator using GROMACS 2021.2 (107, 108, 109).

#### All-atom simulation parameters

Systems were minimized and equilibrated according to the following protocol: i) ≤20,000 steps of steepest descent minimization algorithm (CG2AT2 tool) (98); ii) equilibration with position restraints (force constant = 1000 kJ/mol nm^2^) placed on protein alpha carbons (where present) for 5 ps in the NVT ensemble using CG2AT2, 5 ns in the NVT ensemble, iii) 5 ns in the NPT ensemble; and finally iv) 0.5 μs of unrestrained production runs in the NPT ensemble.

Temperature was maintained at 310 K with the velocity rescale (CG2AT2 equilibration, τ = 0.1 ps), Berendsen (equilibration, τ = 1 ps) or Nosé-Hoover thermostat (110, 111) (production, τ = 1 ps), where separate temperature coupling groups included protein, solvent (water, ions, and PMB1), and lipids. The pressure was maintained semi-isotropically at 1 bar with either the Berendsen barostat (equilibration, τ = 5 ps, compressibility = 4.5 × 10^−5^ bar^−1^) or Parrinello-Rahman barostat (production, τ = 5 ps). Electrostatics were treated using the PME method ($\varepsilon_{r}$ = 1, cutoff = 12 Å) and van der Waals interactions were treated with force-switching applied between 10 to 12 Å. All AA simulations were performed in GROMACS 2021.2 with the leapfrog integrator using a 2 fs timestep.

### Analysis

The LiPyphilic package (112) was used to calculate the self-enrichment index ($E_{\text{AA}}$) for each lipid (cutoff = 12 Å), which was defined as:

1 $$E_{AA}=\frac{N_{AA}}{\left\langle N_{A}\right\rangle }$$

where:

• $N_{\text{AA}}$ = number of molecules of lipid species A around itself in a given frame
• $\left\langle N_{A}\right\rangle$ = time-averaged mean of species A around any species

Lipid order parameters were calculated using modified code from the LiPyphilic package (63). For a single lipid tail the CG order parameter at a given time was defined as:

2 $$S=\frac{\left\langle 3\;\cos^{2}\;\theta -1\right\rangle }{2}$$

where:

• θ = angle between z-axis and vector connecting two consecutive tail beads (Fig. S6*A*)
• ⟨x⟩ = average over all beads in acyl tail

For each lipid we calculated the weighted average of the order parameter over all *m* tails, weighting individual tail order parameters by the number of bead-bead bonds making up that tail:

3 $$S_{\text{total}}=\frac{\sum_{1}^{m}S_{m}\left(N_{m}-1\right)}{\sum_{1}^{m}\left(N_{m}-1\right)}$$

where $S_{m}$ = order parameter for tail m, $N_{m}$ = number of beads in tail m. S_total_ for each lipid was then time-averaged and projected onto the x-y plane.

MDAnalysis (113) tools were used extensively for analyses, including contact analyses, RDFs (Figs. S6*B*) and 2D densities. Per-residue occupancy was calculated as the percentage of (the last μs of) simulation time over which LPS/PMB1 formed contacts with each protein residue (6 Å cutoff). GROMACS was used for the calculation of SASA and PMB1 trajectory clustering. Trajectories for individual PMB1 molecules across all simulations in a given system were concatenated to produce a 'pseudo-trajectory', which was aligned to the first frame and clustered every '400 ps' (CG) or '10 ps' (AA) based on PMB1 backbone and tail atoms using GROMACS tool (GROMOS algorithm). The cutoff for the algorithm ranged between 2.5 to 3.0 Å and was chosen such that the most populated cluster had ∼40 to 70% of all frames (Table S2, Table S3). Molecular visualizations were created in VMD (114), and the *viridis_scales.tcl* script was used (https://github.com/smsaladi/vmd_viridis).

## Data availability

Data are available on request by contacting the corresponding authors SK (syma.khalid@bioch.ox.ac.uk) or PJB (peterjb@bii.a-star.edu.sg).

## Supporting information

This article contains supporting information.

## Conflict of interest

The authors declare that they have no conflicts of interest with the contents of this article.
