# Supplementary material for: Polymyxin B1 in the Escherichia coli inner membrane: A complex story of protein and lipopolysaccharide-mediated insertion
Source: J Biol Chem. 2024 Sep 10;300(10):107754. doi: 10.1016/j.jbc.2024.107754 (PMC11497408; doi:10.1016/j.jbc.2024.107754)
Supplement: Supporting Figures and Tables [file mmc1.docx]

# **Supplementary Information**

Dhanushka Weerakoon^1,2^, Jan K. Marzinek^2^, Conrado Pedebos^3,4^, Peter J. Bond^2,5*^ and Syma Khalid^3*^

1 School of Chemistry, University of Southampton, SO17 1BJ, UK

2 Bioinformatics Institute (BII), Agency for Science, Technology and Research (A*STAR), Singapore 138671, Singapore.

3 Department of Biochemistry, University of Oxford, OX1 3QU, UK

^4^ Programa de Pós-Graduação em Biociências (PPGBio), Universidade Federal de Ciências da Saudé de Porto Alegre - UFCSPA, Brazil

5 Department of Biological Sciences, National University of Singapore, Singapore 117543, Singapore.


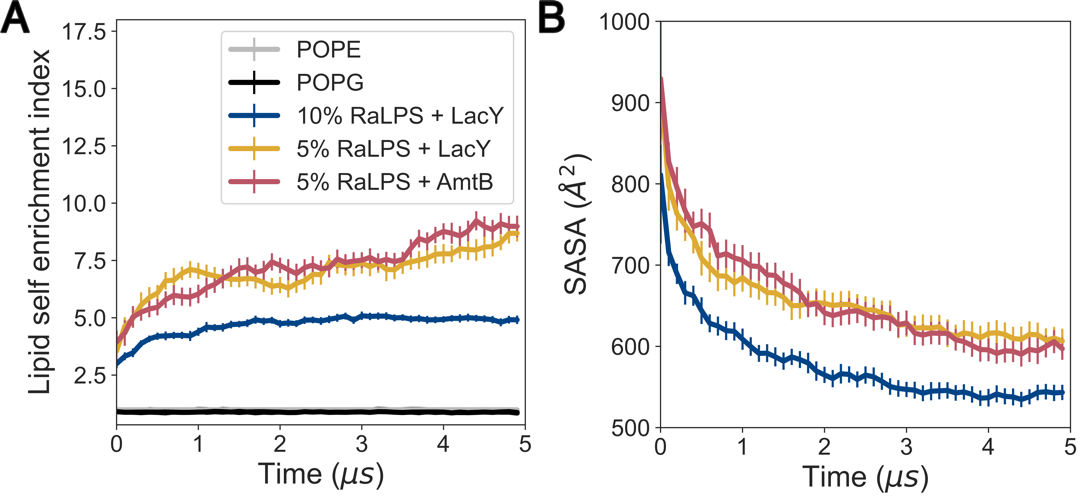


**Figure S1: LPS behaviour in CG simulations (in absence of PMB1 molecules, with membrane proteins). A)** Lipid self-enrichment index over time. **B)** SASA of RaLPS saccharide per RaLPS molecule over time. SASA was calculated relative to surface area of all non-solvent beads, probe radius = 2.6 Å. Systems studied include: RaLPS in 10% LPS + LacY (n=3, dark blue), RaLPS in 5% LPS + LacY (n=3, yellow), RaLPS in 5% LPS + AmtB (n=3, pink), POPE (all systems, n=3, gray), POPG (all systems, n=3, black). Individual trajectories were block averaged in 0.1 μs blocks and subsequently averaged over n repeats. Error bars show 95% confidence intervals.


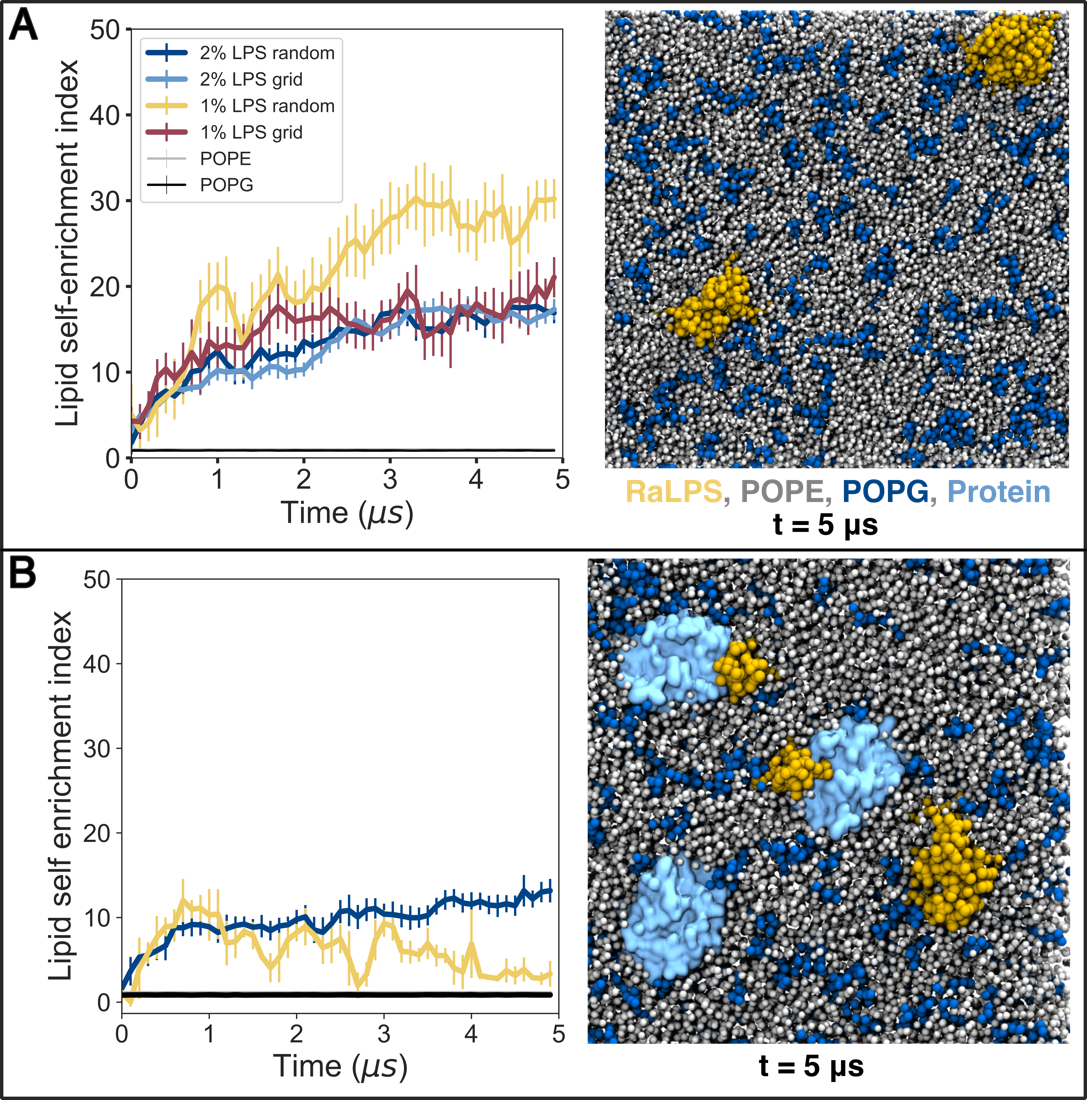


**Figure S2: Aggregation of low quantities of LPS in CG simulations (in absence of PMB1 molecules).** Left: lipid self-enrichment index over time, right: snapshot of representative system at 5 µs. **A)** In absence of membrane proteins; **B**) in presence of membrane proteins (LacY). RaLPS in: 2% random LPS (+ LacY) systems (n=3, dark blue), 2% LPS grid systems (n=3, light blue), 1% random LPS (+ LacY) systems (n=3, yellow), 1% grid LPS systems (n=3, maroon); POPE in all systems (n=3, gray), POPG in all systems (n=3, black). Individual trajectories were block averaged in 0.1 μs blocks and subsequently averaged over n repeats. Error bars show 95% confidence intervals. In snapshots, moieties are represented as: RaLPS = yellow vdW; POPE = gray vdW; POPG = dark blue vdW; protein = light blue surface.


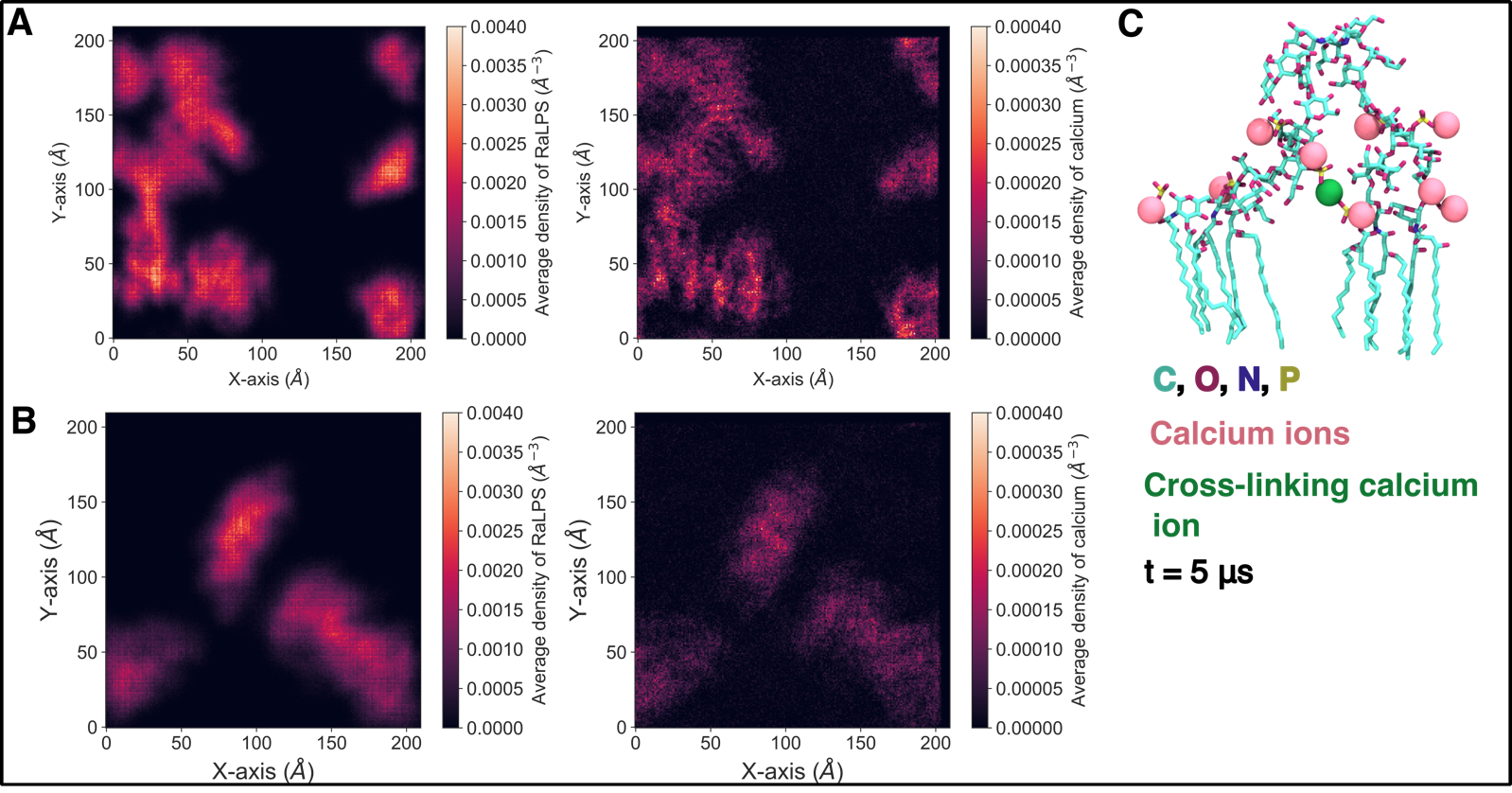


**Figure S3:** **2D plots in plane of membrane showing co-localisation of RaLPS and calcium ions in CG simulations (in absence of PMB1 molecules and membrane proteins). A)** Representative system with 10% LPS. **B)** Representative system with 5% LPS. 2D densities of **left**) RaLPS and **right**) calcium ions measured over last 0.5 μs of simulation. Histogram bin size = 1Å. The results were applicable to all systems investigated, including those with PMB1 molecules and membrane proteins. **C)** Representative backmapped snapshot of two RaLPS molecules interacting with calcium ions. Molecules are represented as RaLPS = licorice, CPK colour scheme + cyan (carbons), calcium ions = pink vdW, cross-linking calcium ions = green vdW.


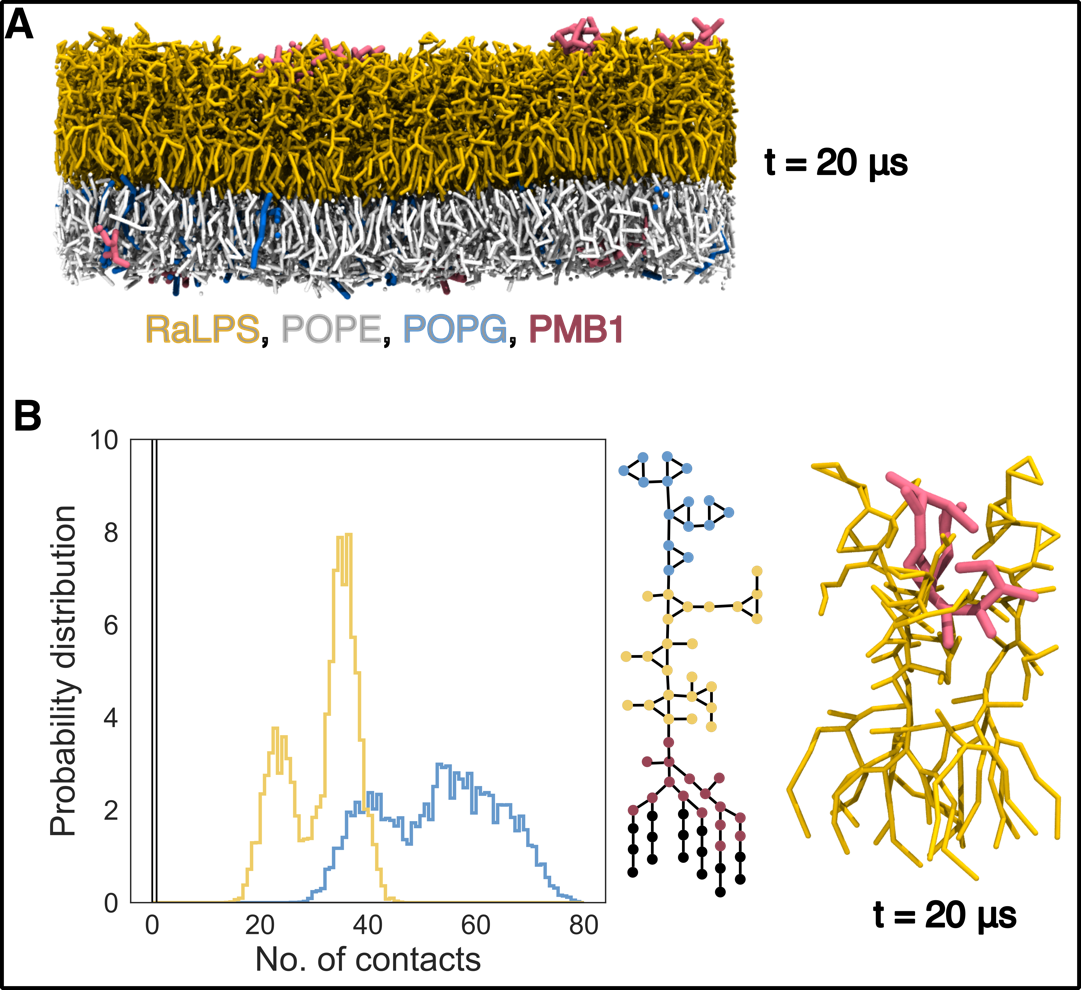


**Figure S4:** **PMB1 behaviour in CG simulations with an outer membrane model. A)** Snapshot from final frame of 20 µs simulation of an asymmetric outer membrane model (outer leaflet: 100% RaLPS, inner leaflet: 90% POPE, 5% POPG, 5% cardiolipin) + 10 PMB1. PMB1 does not penetrate beyond LPS oligosaccharide. **B)** **Left**) Distribution of PMB1-RaLPS moiety contacts in an asymmetric outer membrane model calculated over last 5 µs of simulations; contacts were distributed into 100 bins in range 0-80 and distributions are normalised to 100. Data are shown for: RaLPS outer core saccharide (n=3, blue), RaLPS inner core saccharide (n=3, yellow), RaLPS lipid A headgroup (n=3, dark red, hidden); RaLPS lipid A tails (n=3, black). **Right**) Snapshot of a PMB1 molecule interacting with the core oligosaccharides of three RaLPS molecules. In snapshots, molecules are represented as: RaLPS = yellow licorice; POPE = gray licorice; POPG = blue licorice; PMB1 = pink licorice.


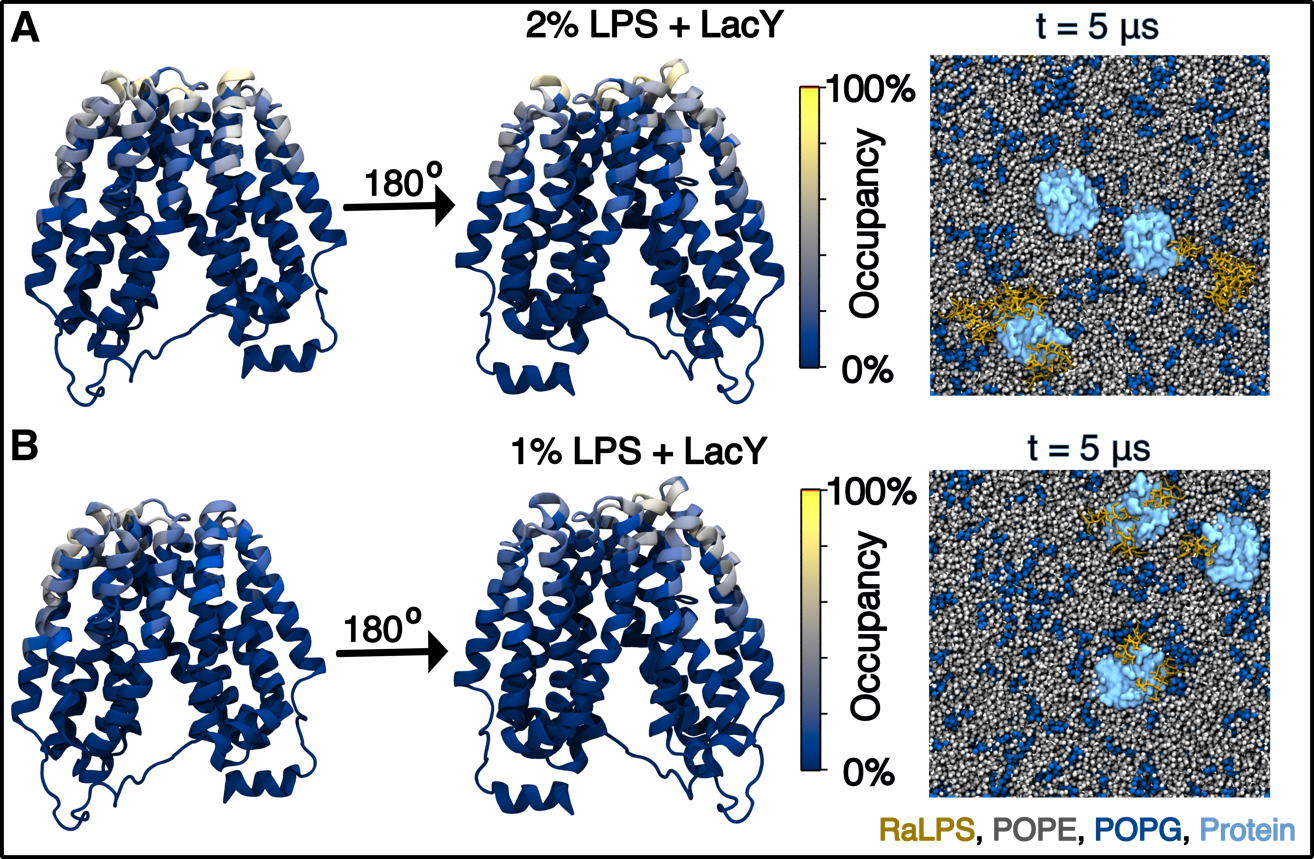


***Figure S5:*** ***RaLPS-protein contacts in CG simulations (in absence of PMB1, with membrane proteins, low LPS content). A. B****)* ***Left****) Occupancy (% over last microsecond of simulation) during which LPS-protein contacts (6 Å cutoff) were formed, calculated on a per-residue basis, mapped onto atomistic protein structures. Proteins are shown in cartoon format and front and back views of protein are shown on the left and right respectively.* ***Right****) Representative snapshots at end of PMB1-free simulations. Molecules are represented as: RaLPS = yellow licorice; POPE = gray vdW; POPG = dark blue vdW; protein = light blue surface.* ***A****) 2% LPS (3 protein copies, n=3),* ***B****) 1% LPS (3 protein copies, n=3).*


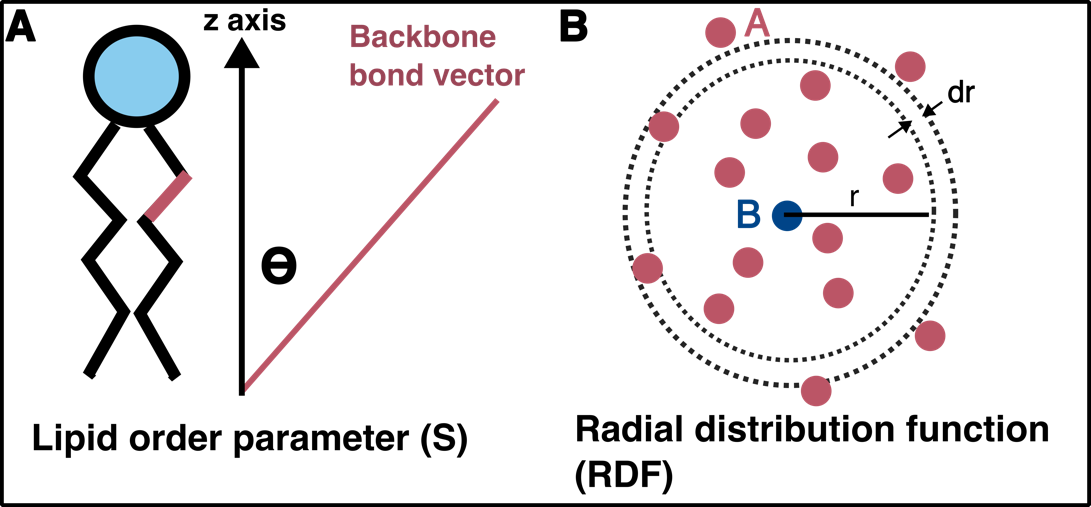


***Figure S6: Schematics representing calculation of*** *A)* ***CG lipid order parameter and*** *B)* ***radial distribution function (RDF).*** ***A****)* $\text{θ}$ *= angle between backbone bond vector and z axis (typically defined as perpendicular to the membrane plane), used to define the CG order parameter S. If S=0, tails are disordered; if S=1, tails are aligned with respect to the z axis.* ***B****) The RDF of one type of particle (A, red beads) with respect to another (B, blue beads) calculates the number of type A beads in an infinitesimally thin shell (thickness = dr) at distance r from a type B bead. The schematic above shows calculation of an RDF in 2D, in this work the calculation is done in 3D. In MD simulations, this calculation is discretised: an upper radial limit for the RDF calculation is set (r = 20 Å), and the spherical volume defined by this upper radial limit is divided into shells of fixed thickness (0.2 Å); the number of beads within each shell is then calculated and divided by the shell volume to give the RDF. RDFs are calculated over all type A and B bead pairs and are time averaged.*


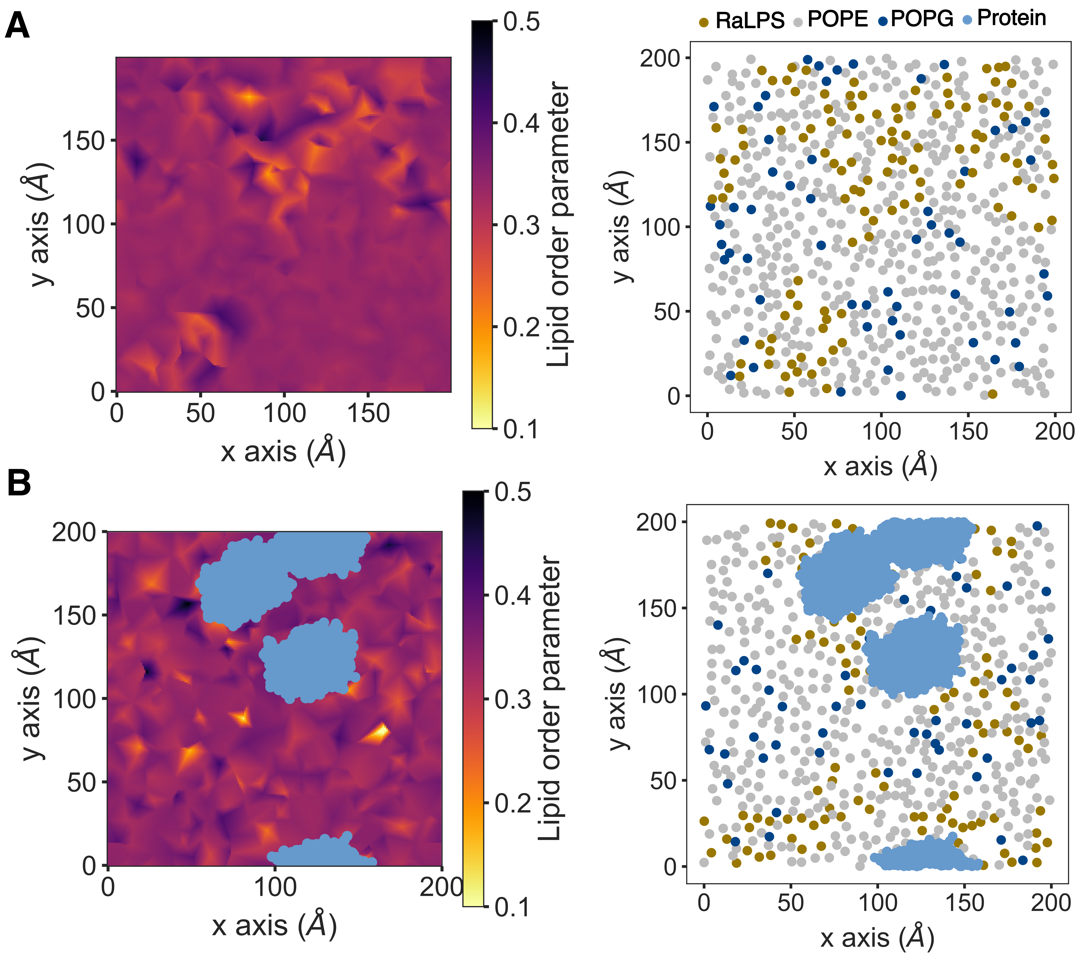


**Figure S7:** **Dynamics of lipids shown in 2D (in plane of membrane) for representative 10% LPS-containing systems (in absence of PMB1, with or without membrane proteins)**. **A)** Data for representative 10% LPS system (no membrane proteins). **B)** Data for representative 10% LPS system + LacY. **Left**) lipid order parameters measured over last 0.5 μs of simulation **–** order parameters can vary from -0.5 to 1.0, with values closer to zero indicating that tails are more disordered, **right**) coordinates of lipids (POPE = gray, POPG = dark blue, RaLPS = dark yellow) and protein (where present, light blue) at t =4.75 µs.


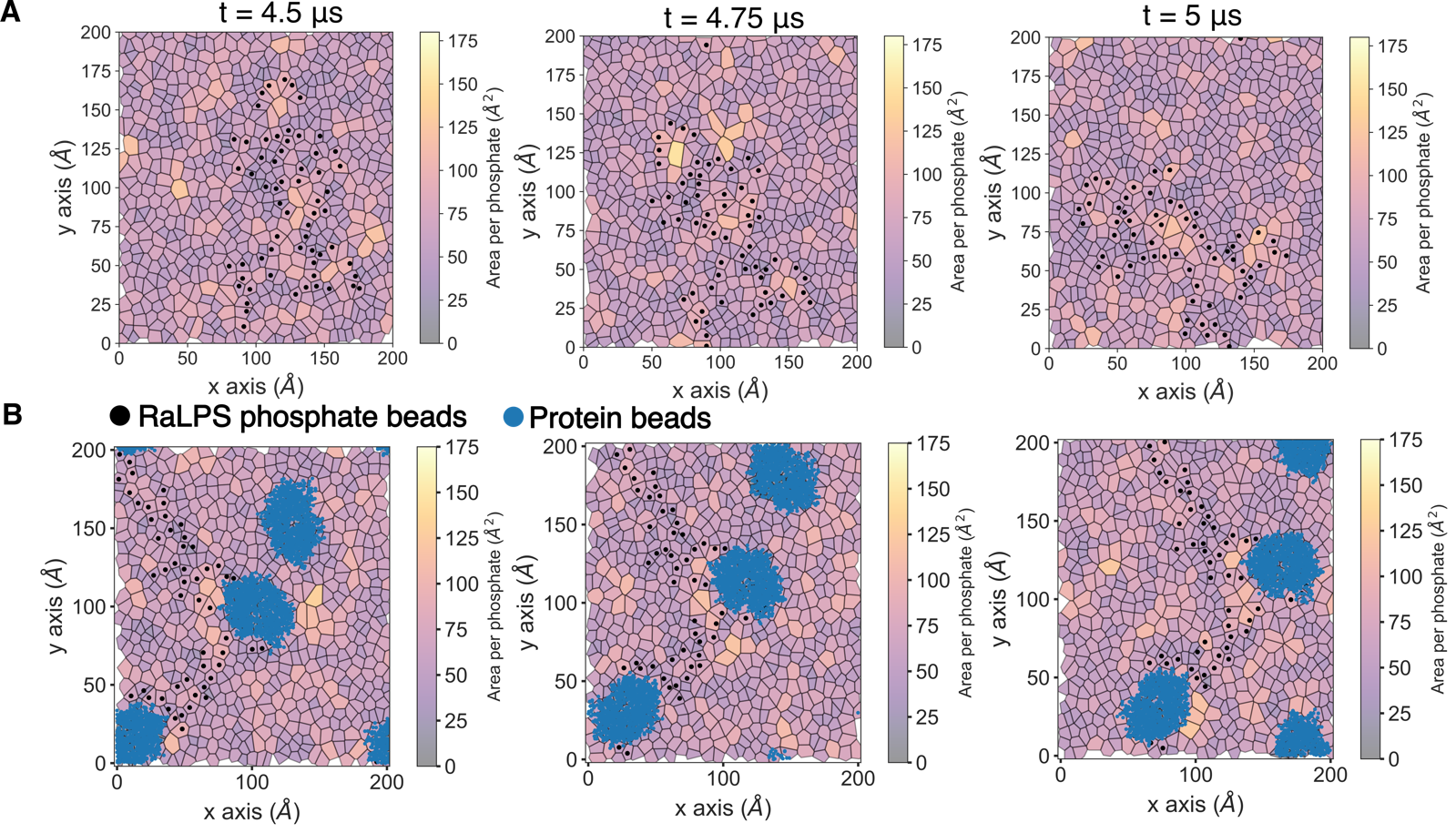


**Figure S8: Area per lipid phosphate of representative 5% LPS-containing systems (in absence of PMB1).
A)** Data for representative 5% LPS system (no membrane proteins). **B)** Data for representative 5% LPS system + LacY proteins. **Left**) t = 4.5 µs, **middle**) t = 4.75 µs, **right**) t= 5 µs. RaLPS headgroup phosphates = black dots, protein backbone beads (used in tesselation) = blue dots.

**Table S1:** **Location of PMB1 molecules at end of each (single bilayer) CG simulation as determined by considering z position of COM of each PMB1.** PMB1 molecules were classified as being inserted if any one of the hydrophobic moieties (PMB1 hydrophobic tail, PMB1 D-Phe, PMB1 Leu residues) lay below the upper leaflet phosphates, otherwise it was classified as surface bound. Numbers in brackets are the numbers of PMB1 molecules in extended simulations.

| **LPS content** | **Proteins?** | **Repeat** | **Number of PMB1 molecules** | | |
| --- | --- | --- | --- | --- | --- |
|  |  |  | **LPS-bound** | **Inserted (upper leaflet)** | **Lower leaflet bound** |
| 10% | None | 1 | 1 | 4 | 5 |
|  |  | 2 | 2 | 6 | 2 |
|  |  | 3 | 2 | 4 | 3 |
|  | LacY | 1 | 0 | 6 | 4 |
|  |  | 2 | 0 | 9 | 1 |
|  |  | 3 | 1 | 6 | 2 |
| 5% | None | 1 | 0 (1) | 7 (12) | 3 (7) |
|  |  | 2 | 1 (1) | 4 (12) | 5 (7) |
|  |  | 3 | 1 (2) | 3 (11) | 5 |
|  | LacY | 1 | 1 (2) | 7 (12) | 2 (6) |
|  |  | 2 | 0 (1) | 6 (13) | 4 (6) |
|  |  | 3 | 2 (0) | 4 (13) | 3 (7) |
|  | AmtB | 1 | 1 | 6 | 3 |
|  |  | 2 | 1 | 8 | 1 |
|  |  | 3 | 2 | 7 | 1 |
| 2% | None | 1 | 0 | 9 | 1 |
|  |  | 2 | 1 | 7 | 2 |
|  |  | 3 | 0 | 8 | 2 |
|  | LacY | 1 | 0 | 8 | 2 |
|  |  | 2 | 1 | 6 | 3 |
|  |  | 3 | 0 | 8 | 2 |
| 1% | None | 1 | 0 | 7 | 3 |
|  |  | 2 | 0 | 7 | 3 |
|  |  | 3 | 0 | 7 | 3 |
|  | LacY | 1 | 0 | 10 | 0 |
|  |  | 2 | 0 | 8 | 2 |
|  |  | 3 | 0 | 3 | 7 |


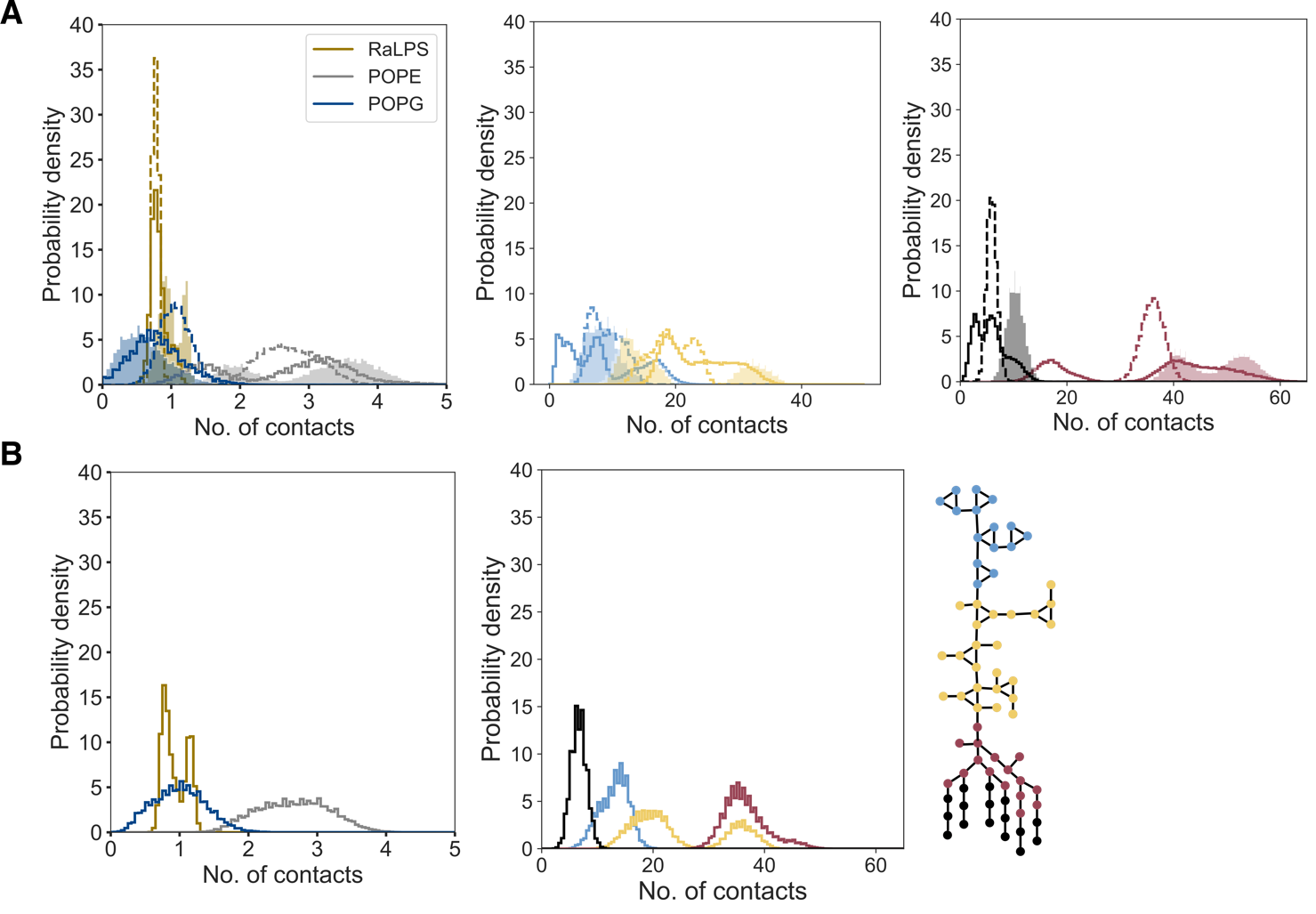


**Figure S9: PMB1-lipid contacts in CG simulations (in presence of membrane proteins).** Distributions shown for: **A**) 10% LPS + LacY + 10 PMB1 (n=3, shaded area), 5% LPS + LacY + 10 PMB1 (n=3, solid line), 5% LPS+ LacY+ 20 PMB1 (n=3, dashed line); **B**) 5% LPS + AmtB (n=3). **Left**) Distributions of PMB1-upper leaflet lipid contacts scaled by the number of upper leaflet-bound PMB1 molecules and number of beads per lipid. Contacts were distributed into 100 bins in range 0-5. Data are shown for: RaLPS (dark yellow, 71 beads), POPE (gray, 12 beads), POPG (dark blue, 12 beads). **Middle**) Distribution of PMB1-LPS core contacts scaled by number of upper leaflet-bound PMB1 molecules. Contacts were distributed into 100 bins in range 0-50. Data are shown for: RaLPS outer core oligosaccharide (light blue), RaLPS inner core oligosaccharide (light yellow). **Right**) Distribution of PMB1-lipid A contacts scaled by number of upper leaflet-bound PMB1 molecules. Contacts were distributed into 100 bins in range 0-50. Data are shown for: RaLPS lipid A headgroup + head/tail interface (maroon), RaLPS lipid A tails (black). (For AmtB, middle and right graphs are combined in one). Contacts were measured over last 5 µs of simulations for data in **A**, **B** and distributions were normalised to 100.


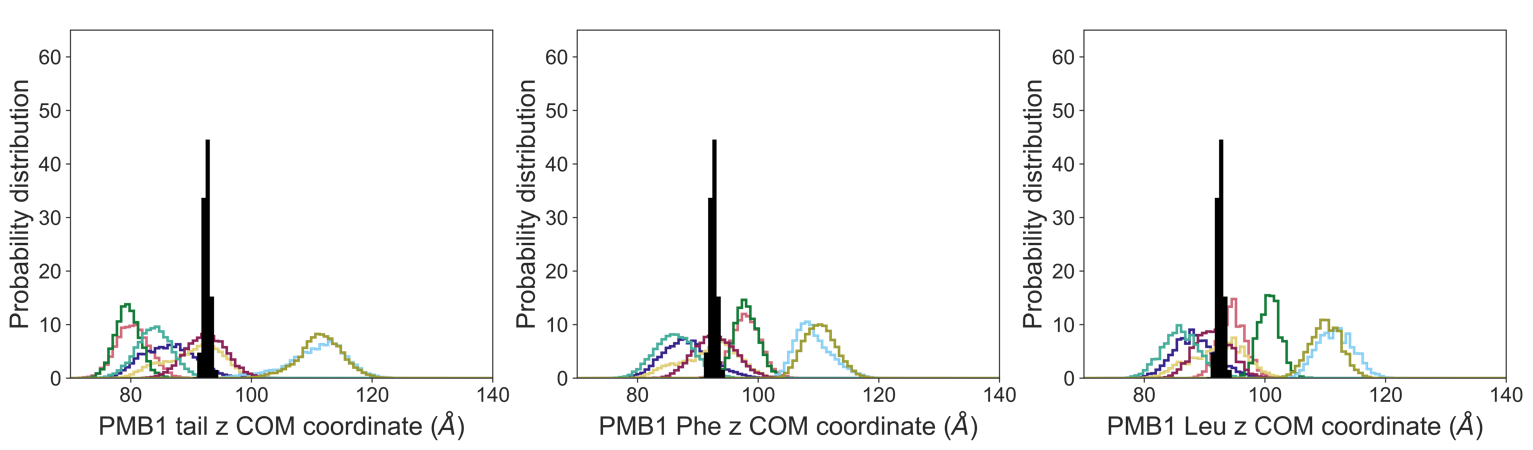


**Figure S10: Insertion of PMB1 hydrophobic moieties into the lipid tail region of the membrane.** Distribution of z-coordinate of COM of different hydrophobic moieties of individual upper-leaflet bound PMB1 molecules for a representative simulation (10% LPS + 10 PMB1): **left**) PMB1 hydrophobic tail, **middle**) Phe, and **right**) D-Leu. Contacts were calculated over last 5 µs of simulation. Data are shown for: individual PMB1 molecules = colored lines, upper leaflet phosphates = shaded black areas. z-coordinates were distributed into 100 bins in range 70-140 Å and distributions were normalised to 100.


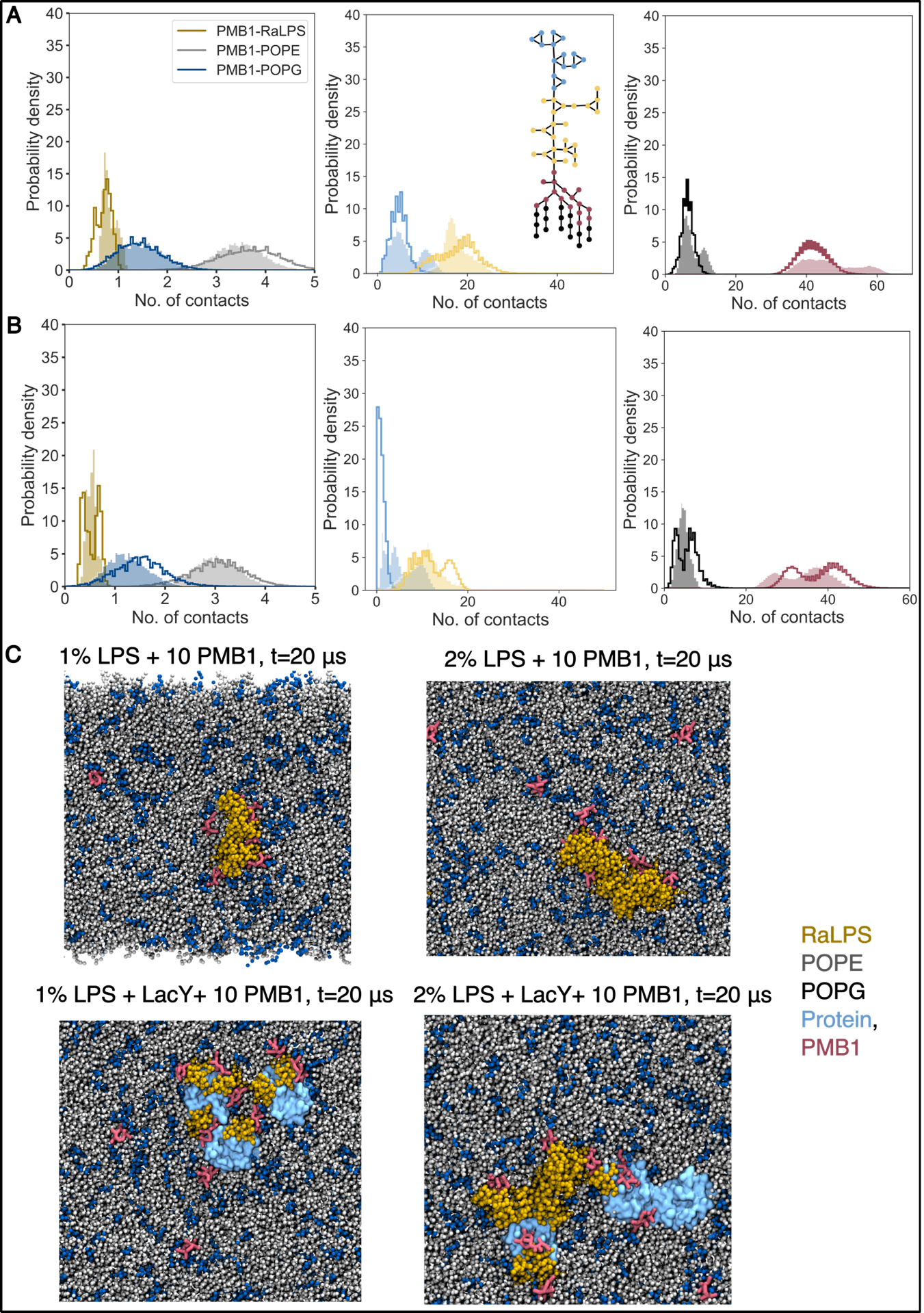


**Figure S11: PMB1-lipid contacts in CG simulations containing low LPS quantities.** Distributions shown for: **A**) 2% LPS (n=3, shaded area), 1% LPS (n=3, solid line); **B**) 2% LPS + LacY (n=3, shaded area), 1% LPS + LacY (n=3, solid line). **Left**) Distributions of PMB1-upper leaflet lipid contacts scaled by the number of upper leaflet-bound PMB1 molecules and number of beads per lipid. Contacts were distributed into 100 bins in range 0-5. Data are shown for: RaLPS (dark yellow, 71 beads), POPE (gray, 12 beads), POPG (dark blue, 12 beads). **Middle**) Distribution of PMB1-LPS core contacts scaled by number of upper leaflet-bound PMB1 molecules. Contacts were distributed into 100 bins in range 0-50. Data are shown for: RaLPS outer core oligosaccharide (light blue), RaLPS inner core oligosaccharide (light yellow). **Right**) Distribution of PMB1-Lipid A contacts scaled by number of upper leaflet-bound PMB1 molecules. Contacts were distributed into 100 bins in range 0-50. Data are shown for: RaLPS lipid A headgroup (dark red), RaLPS lipid A tails (black). Contacts were measured over last 5 µs of simulations for data in **A**, **B** and distributions were normalised to 100. **C**) Representative snapshots of low LPS content, PMB1-containing systems at 20 µs. Molecules are represented as: RaLPS = yellow vdW; POPE = gray vdW; POPG = dark blue vdW; protein = light blue surface; PMB1 = pink licorice.


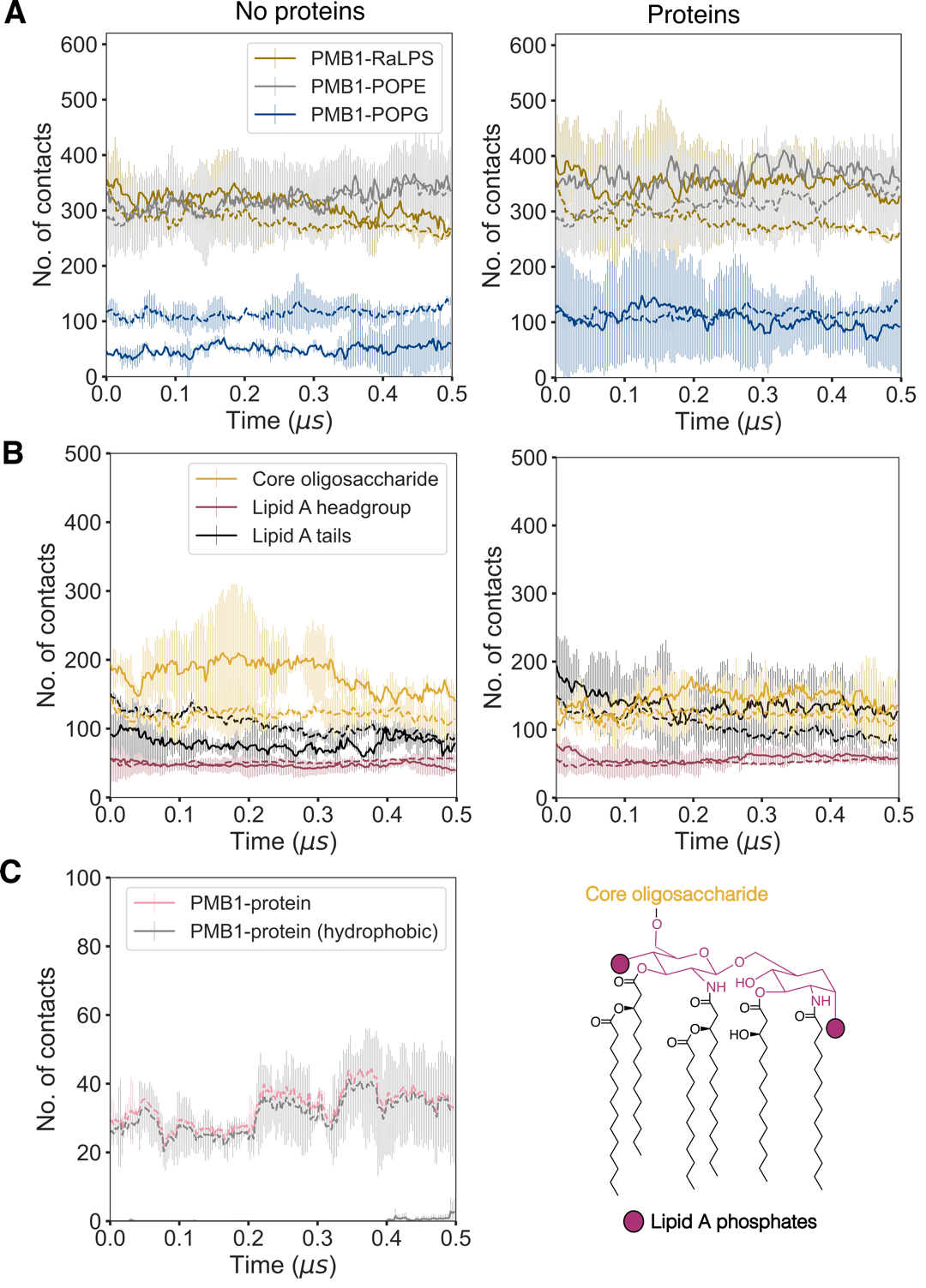


**Figure S12: PMB1-lipid and PMB1-protein contacts timeseries in all-atom simulations.** Data are shown for: 10% LPS + 10 PMB1 (n=2, left, bold lines), 5% LPS + 20 PMB1 (n=2, left, dashed lines), 10% LPS + LacY + 10 PMB1 (n=2, right, solid lines), 5% LPS + LacY + 20 PMB1 (n=2, right, dashed lines). Contacts were scaled by the number of upper leaflet-bound PMB1 molecules. **A**) PMB1-lipid contact timeseries. Data are shown for: RaLPS (dark yellow), POPE (gray), POPG (dark blue). **B**) PMB1-LPS moiety contact timeseries. Data are shown for: core oligosaccharide (yellow), lipid A headgroup (dark red), lipid A tails (black). **C**) PMB1-protein contacts. **Left**) Overall contacts, **right**) Hydrophobic PMB1 (C, H atom)-protein (C, H atom) contacts. Contacts were calculated within a 4 Å cutoff. Individual trajectories were block averaged in 2.5 ns blocks and averaged over n repeats. Error bars show 95% confidence intervals

***
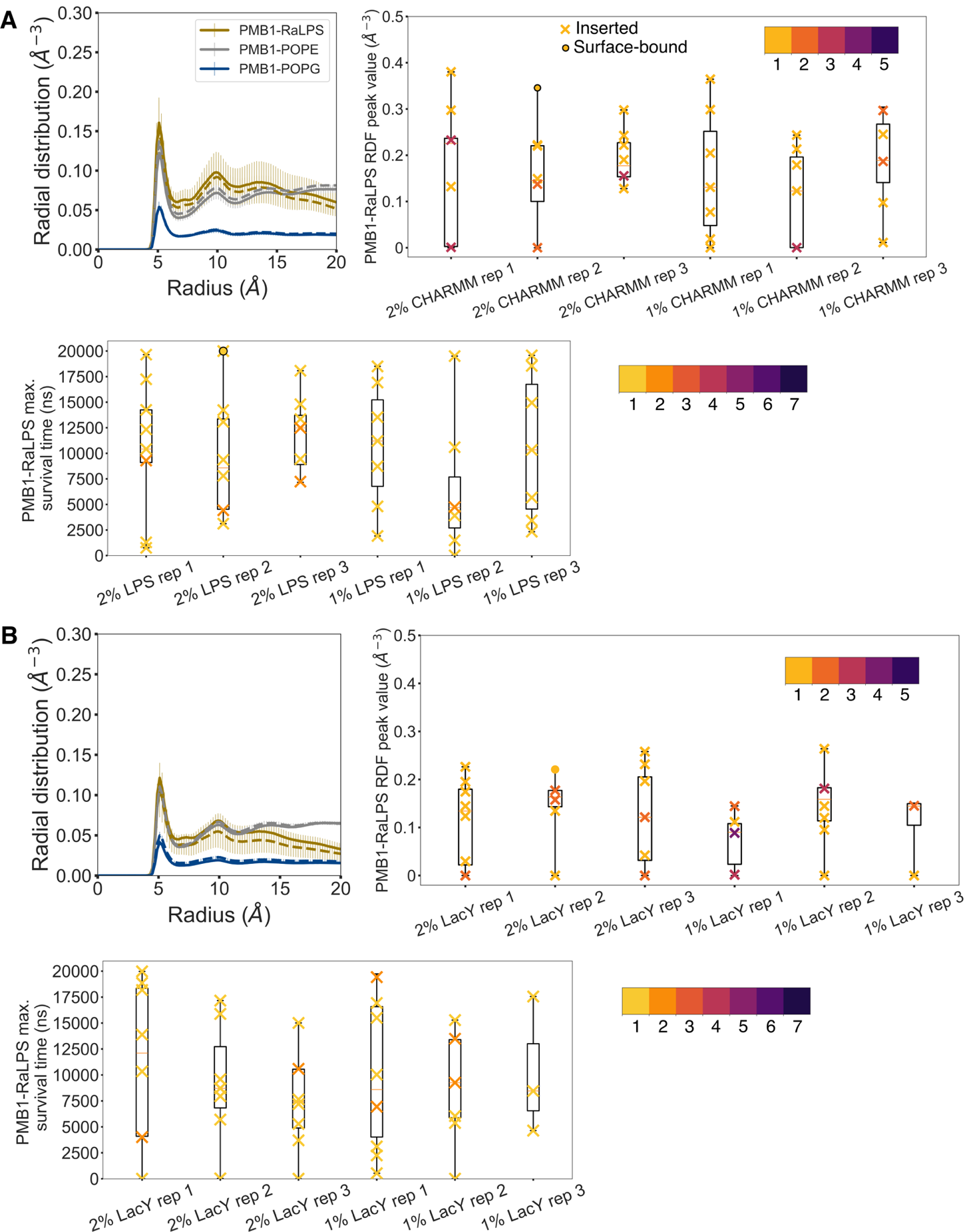
***

***Figure S13: Environment of PMB1 molecules in CG simulations with low LPS content. A)*** *In the absence of membrane proteins,* ***B****) with membrane proteins (LacY) included.* ***Left)*** *RDFs of upper leaflet inserted PMB1 molecules relative to different lipids: RaLPS (dark yellow), POPE (gray), POPG (dark blue). Distributions are shown for: 2% LPS (+LacY) + 10 PMB1 (n=3, solid), 1% LPS (+LacY) + 10 PMB1 (n=3, dashed). RDFs were calculated for radii from 0 to 20 Å (bin width = 0.2 Å) and normalized by relevant number of PMB1 molecules only. Data was averaged over n repeats, error bars show 95% confidence intervals.* ***Right****) Box and scatter plots of individual upper leaflet-bound PMB1-RaLPS RDF peak values (~6 Å) for individual simulations, calculation details similar to collective RDFs. Boxes span the interquartile range (IQR) of data, whiskers span minimum and maximum values. Data point multiplicity is indicated by color bar. Inserted PMB1 molecules are depicted as crosses and surface-bound PMB1 molecules as circles.*

*
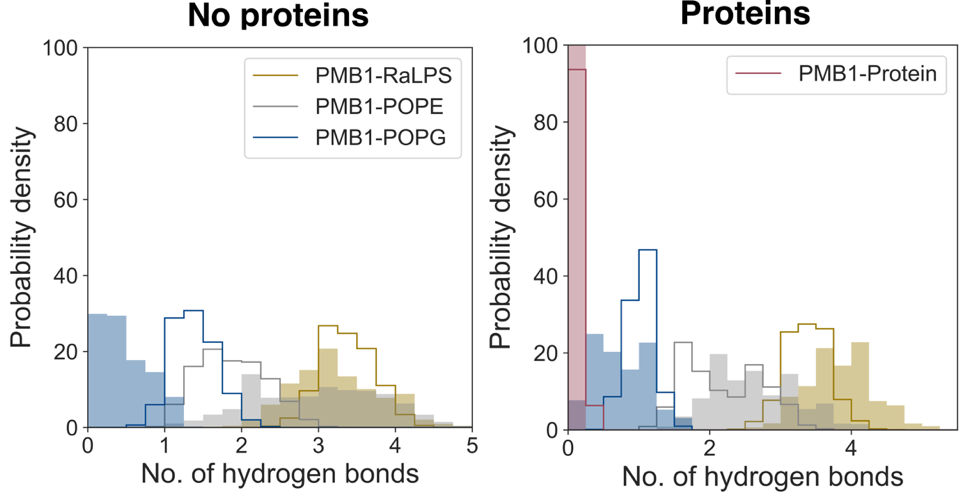
*

***Figure S14: Hydrogen bonds involving PMB1 molecules formed in all-atom simulations.*** *Distribution of number of hydrogen bonds formed between upper leaflet PMB1 molecules and lipids/proteins calculated from 0.4-0.5 μs scaled by number of upper leaflet-bound PMB1 molecules.* ***Left****) Protein-free systems (10% LPS + 10 PMB1 (n=2, shaded), 5% LPS + 20 PMB1 (n=2, line)),* ***Right****) protein-containing systems (10% LPS + LacY + 10 PMB1 (n=2, shaded), 5% LPS + LacY + 20 PMB1 (n=2, line)). Data are shown for: RaLPS = yellow, POPE = gray, POPG = blue, protein = red. In both cases, data was distributed into 40 bins in range 0-10 and each distribution was normalized to 100.*


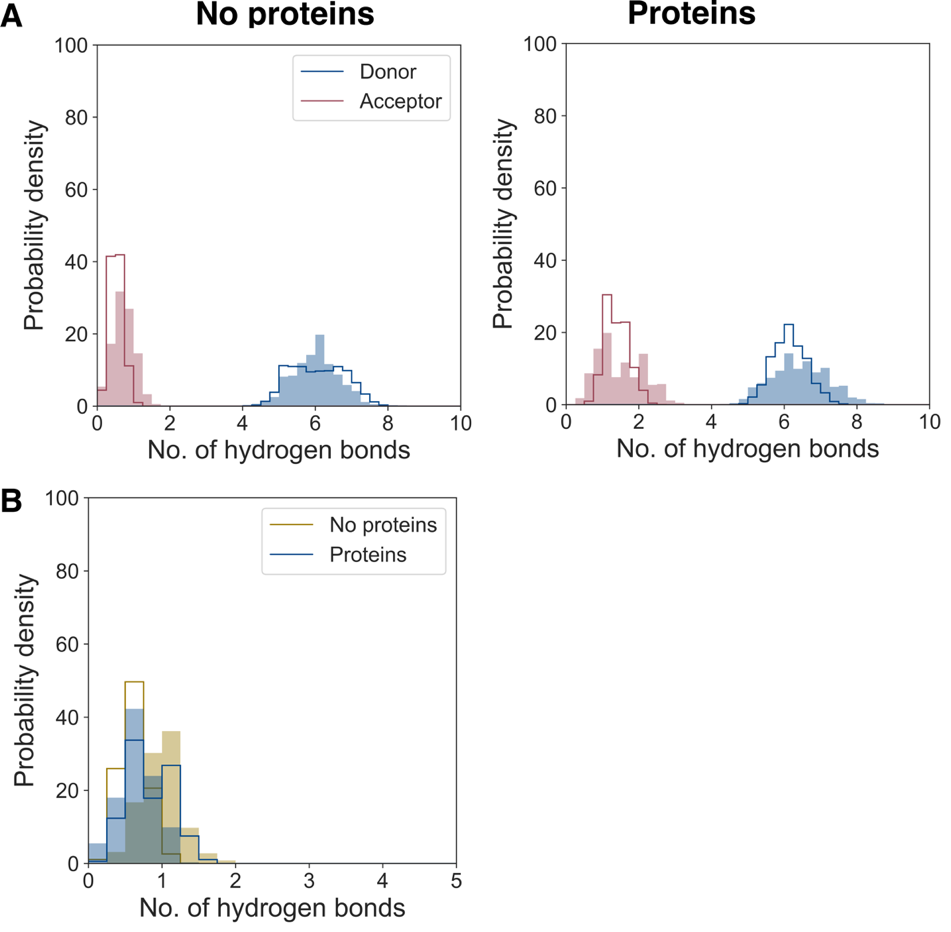


**Figure S15:** **Detailed analysis of hydrogen bonds in all-atom simulations. A)** Distribution of number of hydrogen bonds formed between upper leaflet PMB1 molecules and lipids/protein in which PMB1 acted as an acceptor (red) or as a donor (blue) calculated from 0.4-0.5 μs scaled by number of upper leaflet PMB1 bound molecules. **Left**) 10% LPS + 10 PMB1 (n=2, shaded area), 5% LPS + 20 PMB1 (n=2, dashed line); **right**) 10% LPS + 3 LacY + 10 PMB1 (n=2, shaded area), 5% LPS + 3 LacY + 20 PMB1 (dashed line). Data was distributed into 40 bins in range 0-10. **B)** Distribution of number of intramolecular PMB1 hydrogen bonds. Data are shown for: 10% LPS + 10 PMB1 (n=2, yellow, shaded area), 5% LPS + 20 PMB1 (n=2, yellow, line), 5% LPS + 3 LacY + 10 PMB1 (n=2, blue, shaded area), 5% LPS + 3 LacY + 20 PMB1 (n=2, blue, line). Data was distributed into 20 bins in range 0-5. All distributions (**A,B**) were normalised to 100.


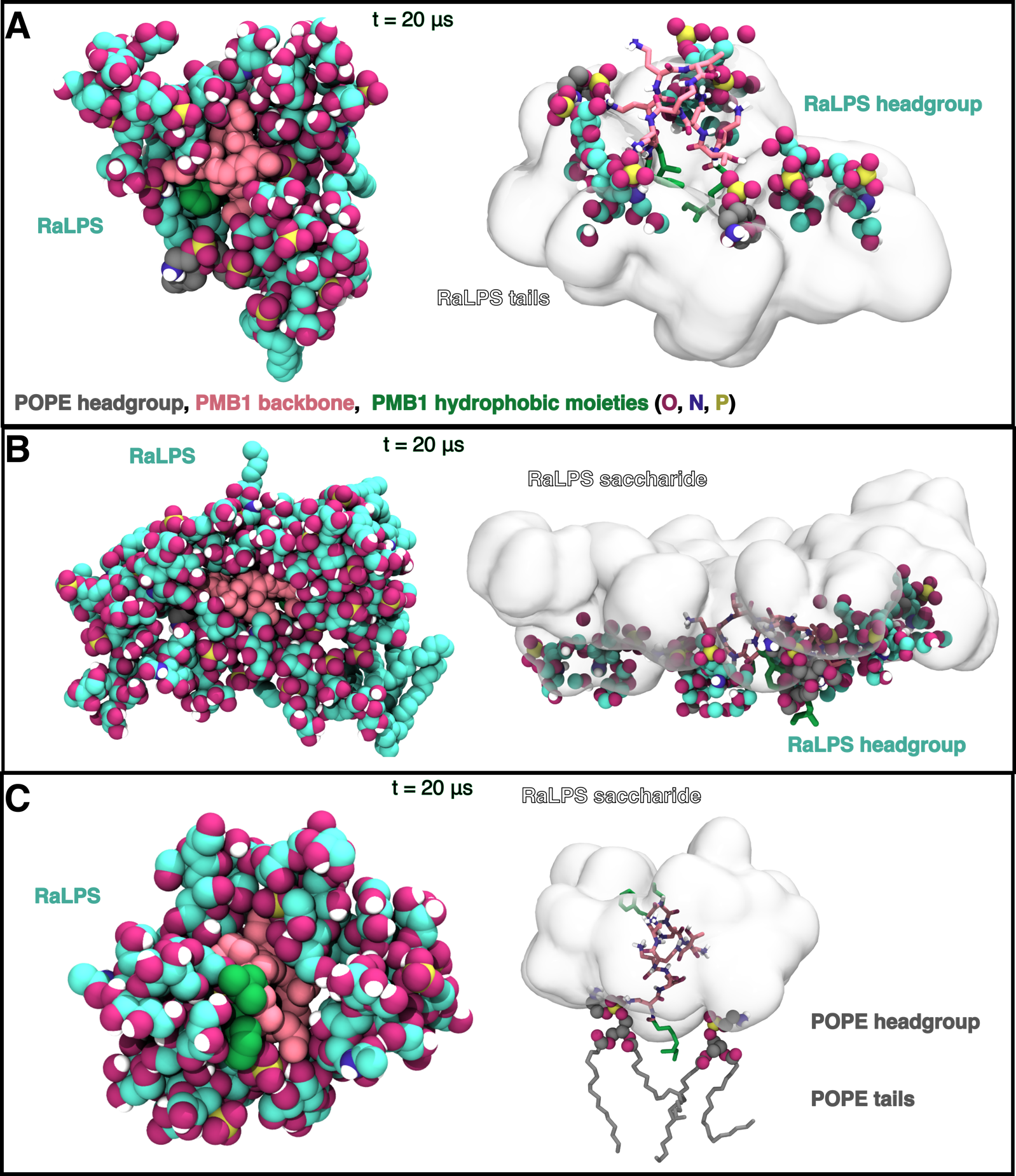


***Figure S16: Backmapped snapshots (from CG simulations, t = 20 µs) of PMB1 molecules that appear to be inserted into LPS phase. A)*** *PMB1 molecule at end of simulation of 5% LPS + 10 PMB1.* ***B)*** *PMB1 molecule at end of simulation of 5% LPS + 20 PMB1.* ***C)*** *PMB1 molecule at end of 5% LPS + AmtB + 10 PMB1 simulation* ***In each panel: left****) top-down view. Molecules are represented as: RaLPS = vdW, CPK color scheme + light blue (carbons); PMB1 = pink vdW; PMB1 hydrophobic moieties = green vdW.* ***Right****) side view. Moieties are represented as: RaLPS saccharide = transparent surface (B, C); RaLPS headgroups = vdW, CPK colour scheme + light blue (carbons) (A, B); POPE, POPG headgroups = vdW, CPK color scheme + gray (carbons); RaLPS tails = transparent surface (A); POPE, POPG tails = licorice, CPK color scheme + gray (carbons) (C); PMB1 = licorice, CPK color scheme + pink (carbons); PMB1 hydrophobic moieties = green licorice.*


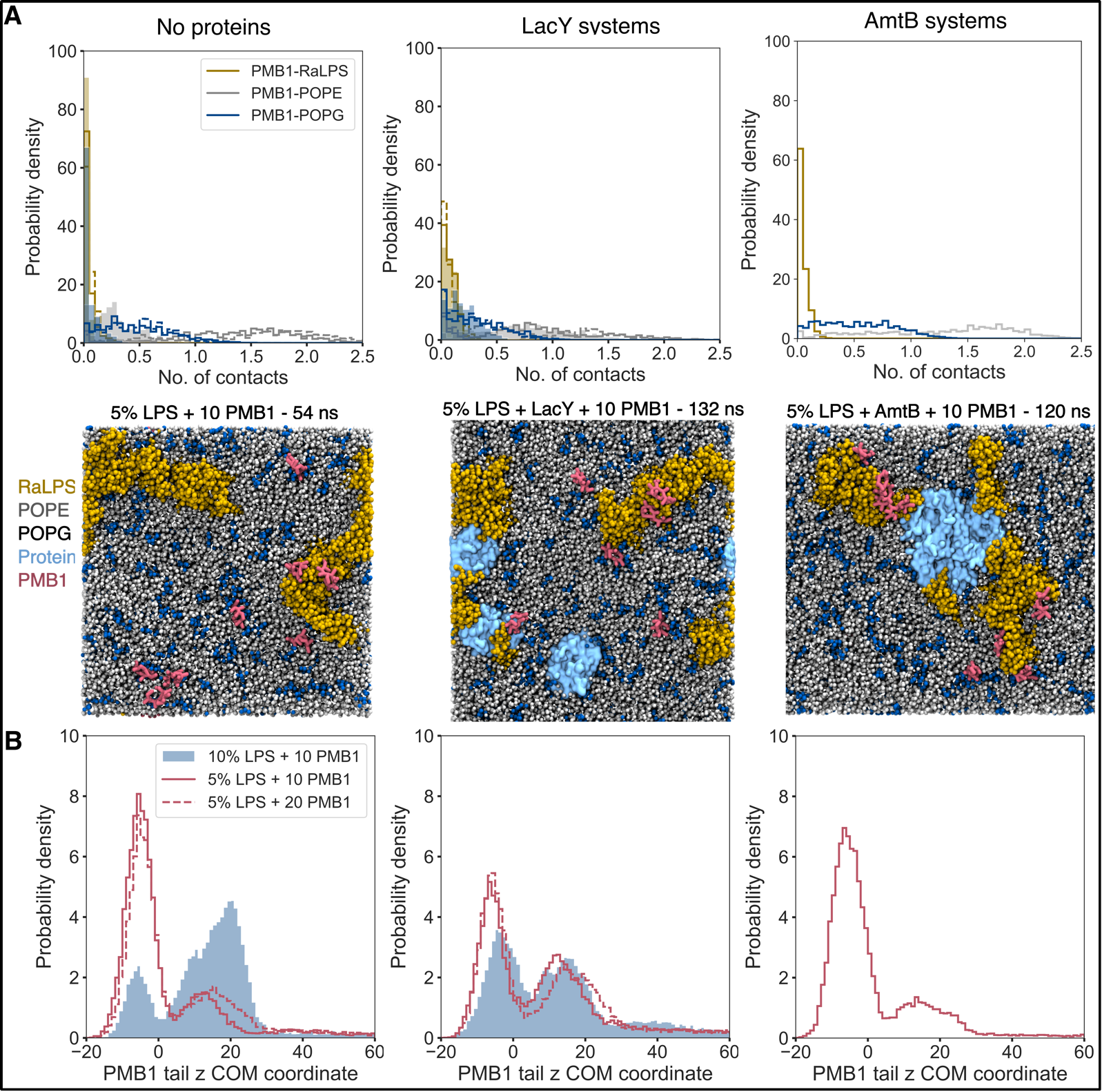


**Figure S17: Behaviour of PMB1 molecules (which are inserted within 20 µs) in the first 0.5 μs of simulations. A) Top:** Distribution of (newly) inserted PMB1-lipid tail contacts scaled by number of inserted leaflet-bound PMB1 molecules and number of lipid tail beads per lipid. Contacts were distributed into 100 bins in range 0-5. Data are shown for: RaLPS (yellow, 16 beads), POPE (gray, 8 beads), POPG (blue, 8 beads). **Bottom**: Representative snapshots at time at which all PMB1 molecules are membrane-bound. Molecules are represented as: RaLPS = yellow vdW; POPE = gray vdW; POPG = dark blue vdW; protein = light blue surface. **B)** Distribution of [z-coordinate of COM of PMB1 hydrophobic tail - z coordinate of upper leaflet phosphates]. Coordinates were distributed into 150 bins in the range -20-130 Å (-20-60 Å shown). Coordinates < 0 Å indicate PMB1 insertion. **Left**) Protein-free systems, **centre**) LacY systems, **right**) AmtB systems. For both **A** and **B**, data are shown for: 10% LPS + 10 PMB1 (n=3, shaded area, left, blue (B)), 10% LPS + LacY + 10 PMB1 (n=3, shaded area, middle, blue (B)), 5% LPS + 10 PMB1 (n=3, solid line, left, red (B)), 5% LPS + LacY + 10 PMB1 (n=3, solid line, middle, red (B)), 5% LPS + 20 PMB1 (n=3, dashed line, left, red (B)), 5% LPS + LacY + 20 PMB1 (n=3, dashed line, middle, red (B)), 5% LPS + AmtB + 10 PMB1 (n=3, right, red (B)). All distributions in **A**, **B** were normalized to 100.


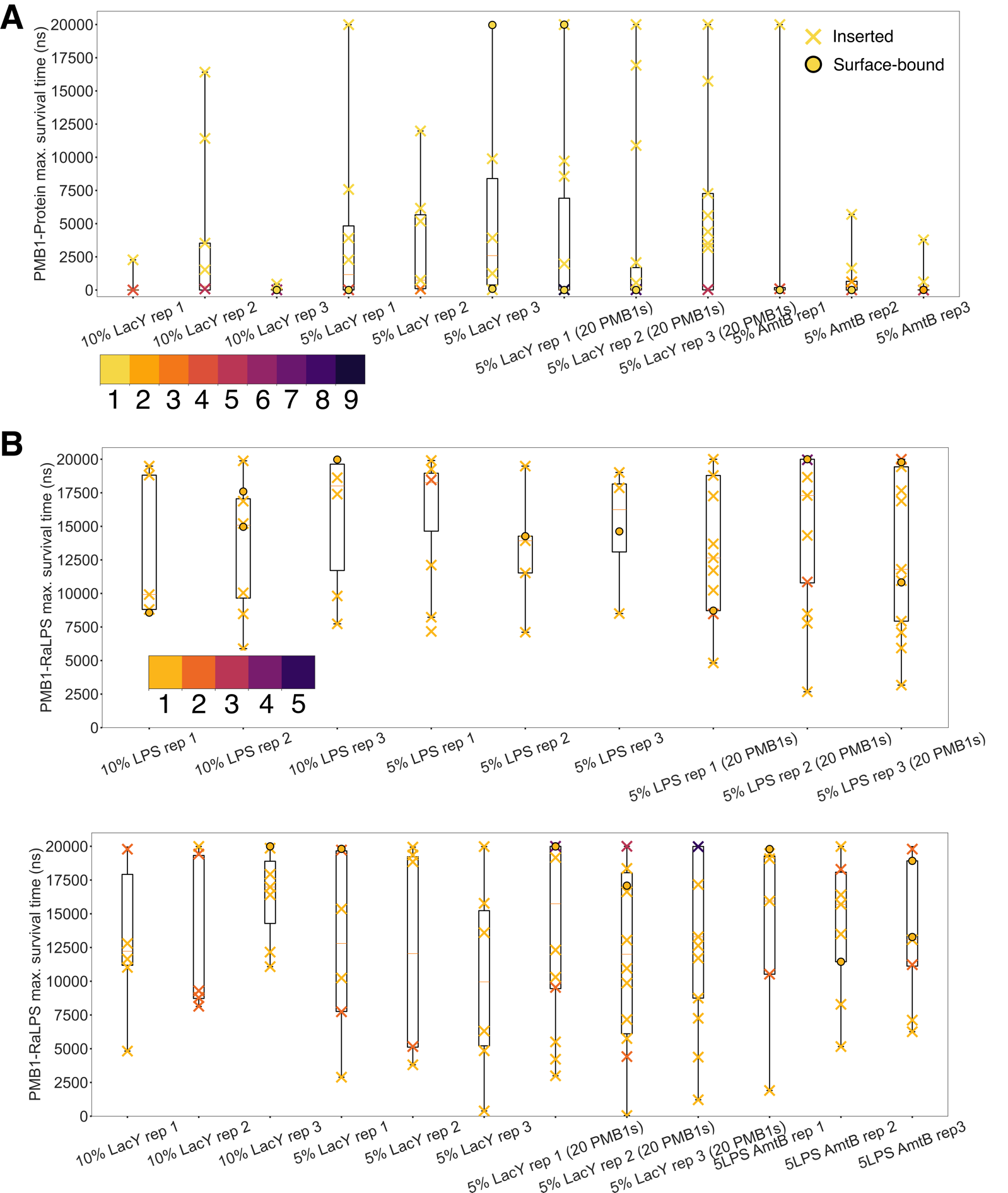


***Figure S18: PMB1-protein and PMB1-RaLPS lifetimes in CG simulations (in absence and presence of membrane proteins).*** *Box and scatter plots of* ***A****) maximum protein residue interaction,* ***B****) maximum RaLPS interaction times (****top****) in absence and (****bottom****) in presence of membrane proteins) for individual upper leaflet bound PMB1 molecules measured over entire trajectory. The boxes span the interquartile range (IQR) of the data, while the whiskers extend to the minimum and maximum values. Data points are colored by multiplicity according to the color bars (for* ***A*** *and* ***B*** *respectively). Inserted PMB1 molecules are depicted as crosses and surface-bound PMB1 molecules as circles.*


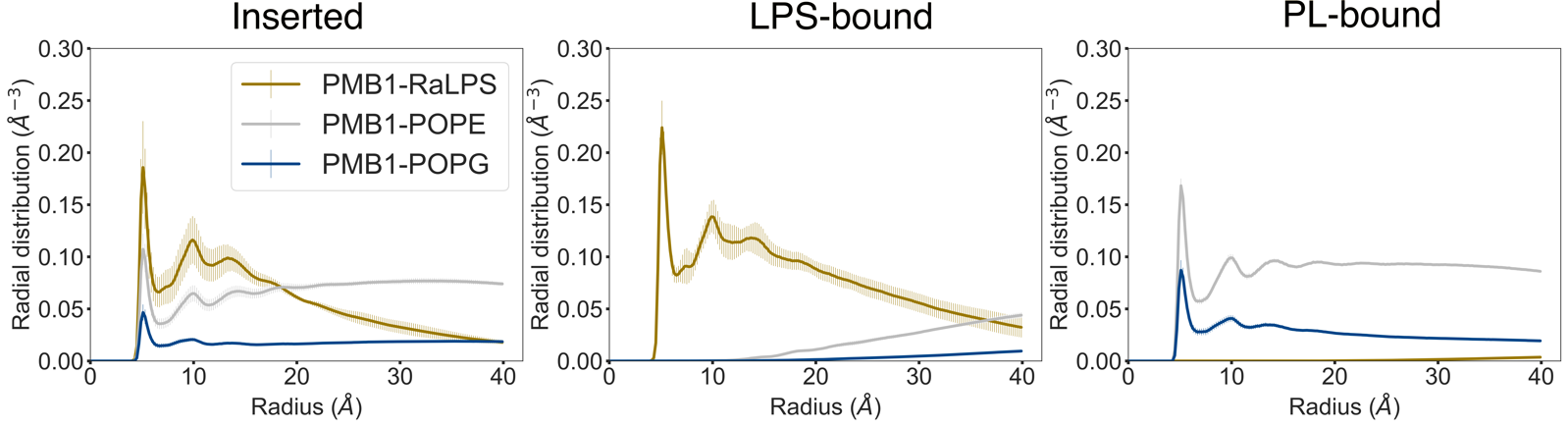


**Figure S19:** **Environment of PMB1 molecules in CG simulations (in presence of AmtB).** Environments of PMB1 molecules within the membrane: **left**) upper leaflet-inserted PMB1, **middle**) LPS-bound PMB1, **right**) lower leaflet-inserted PMB1 measured over last microsecond of simulations. RDFs were calculated for radii from 0 to 20 Å (bin width = 0.2 Å) and normalized by relevant number of PMB1 molecules only. Data are shown for: RaLPS (yellow), POPE (gray), POPG (blue).


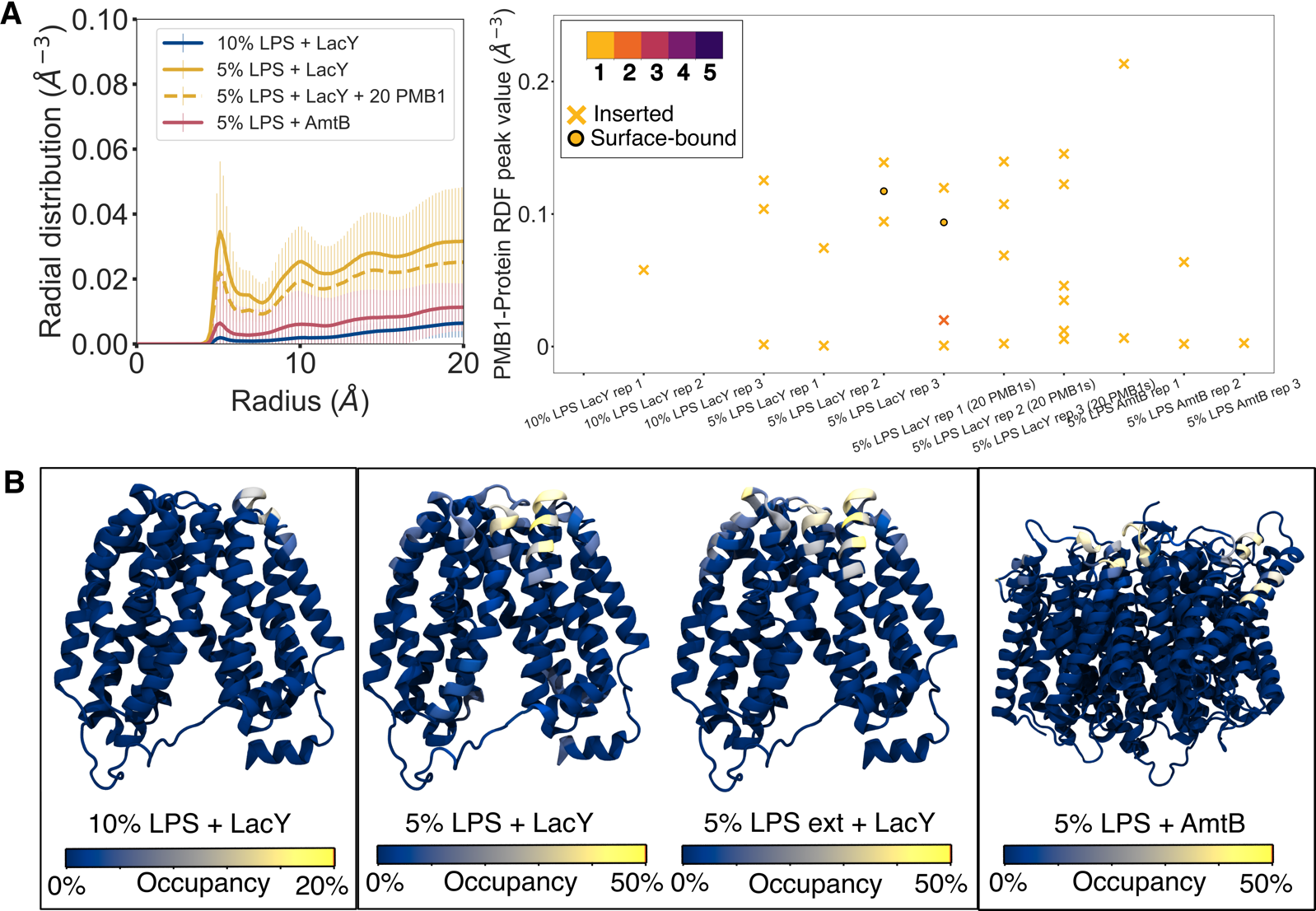


**Figure S20**: **Details of PMB1-protein interactions in CG simulations.** **A) left**) collective RDFs of inserted PMB1 molecules relative to proteins measured over last microsecond of simulations. RDFs were calculated for radii from 0 to 20 Å (bin width = 0.2 Å) and normalized by relevant number of PMB1 molecules only. Data are shown for: 10% LPS + 3 LacY + 10 PMB1 (n=3, solid line, blue), 5% LPS + 3 LacY + 10 PMB1 (n=3, solid line, yellow), 5% LPS + 3 LacY + 20 PMB1 (n=3, dashed line, yellow) , 5% LPS + AmtB + 10 PMB1 (n=3, dashed line, red). Data was averaged over n repeats, error bars = 95% confidence intervals. **Right**) scatter plots of individual upper leaflet-bound PMB1-protein RDF peak values (~ 6 Å), calculation details similar to collective RDFs. Values equal to zero were filtered out, and data point multiplicity is indicated by the color bar. **B)** Occupancy percentage of trajectory over which upper leaflet-bound PMB1-protein (6 Å cutoff) contacts were formed, calculated on a per-residue basis and mapped onto atomistic protein structures. Proteins are shown in cartoon format. 10% LPS + LacY (averaged over 3 protein copies, n=3), 5% LPS + LacY (3 protein copies, n=3), 5% LPS ext + LacY (3 protein copies, n=3), 5% LPS + AmtB (n=3).

***
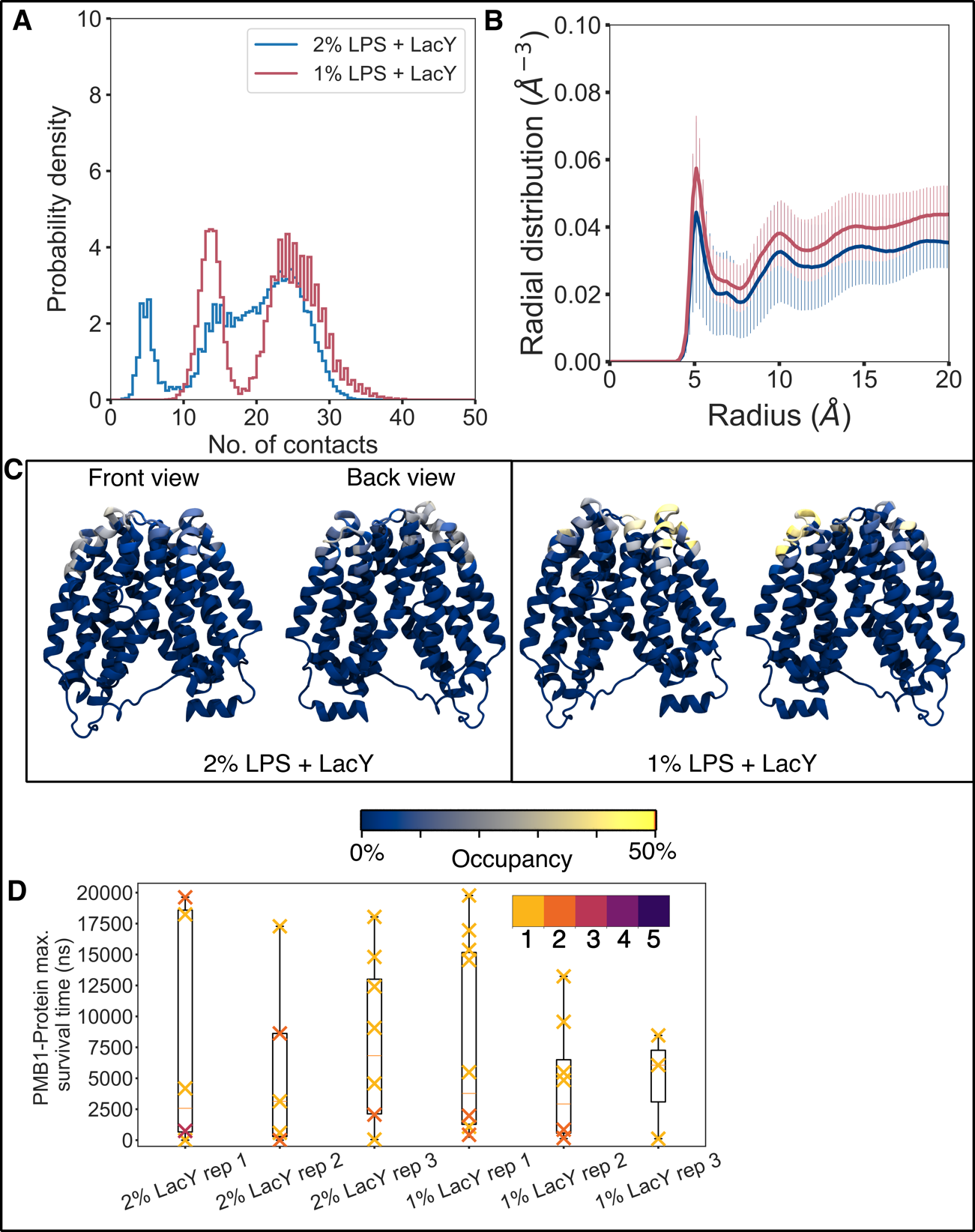
+***

***Figure S21: PMB1-protein interactions in CG simulations with low LPS content. A)*** *Distribution of upper leaflet bound PMB1-protein contacts (measured over last 5 µs of simulations) scaled by the number of upper leaflet-bound PMB1 molecules. Contacts were distributed into 100 bins in range 0-5 and distributions were normalised to 100.* ***B)*** *RDFs of upper leaflet inserted PMB1 molecules relative to protein. RDFs were calculated for radii from 0 to 20 Å (bin width = 0.2 Å), and normalized by relevant number of PMB1 molecules only. For* ***A*** *and* ***B****, distributions are shown for: 2% LPS + LacY + 10 PMB1 (n=3, blue) and 1% LPS + LacY + 10 PMB1 (n=3, maroon). Data was averaged over n repeats, error bars show 95% confidence intervals.* ***C)*** *Occupancy percentage of trajectory over which upper leaflet-bound PMB1-protein (6 Å cutoff) contacts are formed, calculated on a per-residue basis and mapped onto atomistic protein structures. Proteins are shown in cartoon format and front and back views of protein are shown on the left and right respectively. 2% LPS + LacY (averaged over 3 protein copies, n=3), 1% LPS + LacY (averaged over 3 protein copies, n=3).* ***D****) Maximum protein interaction times for individual upper leaflet-bound PMB1 molecules measured over entire trajectory. The boxes span the interquartile range (IQR) of the data, while the whiskers extend to the minimum and maximum values. Data points are colored by multiplicity according to the color bars (for* ***A*** *and* ***B*** *respectively). Inserted PMB1 molecules are depicted as crosses and surface-bound PMB1 molecules as circles.*


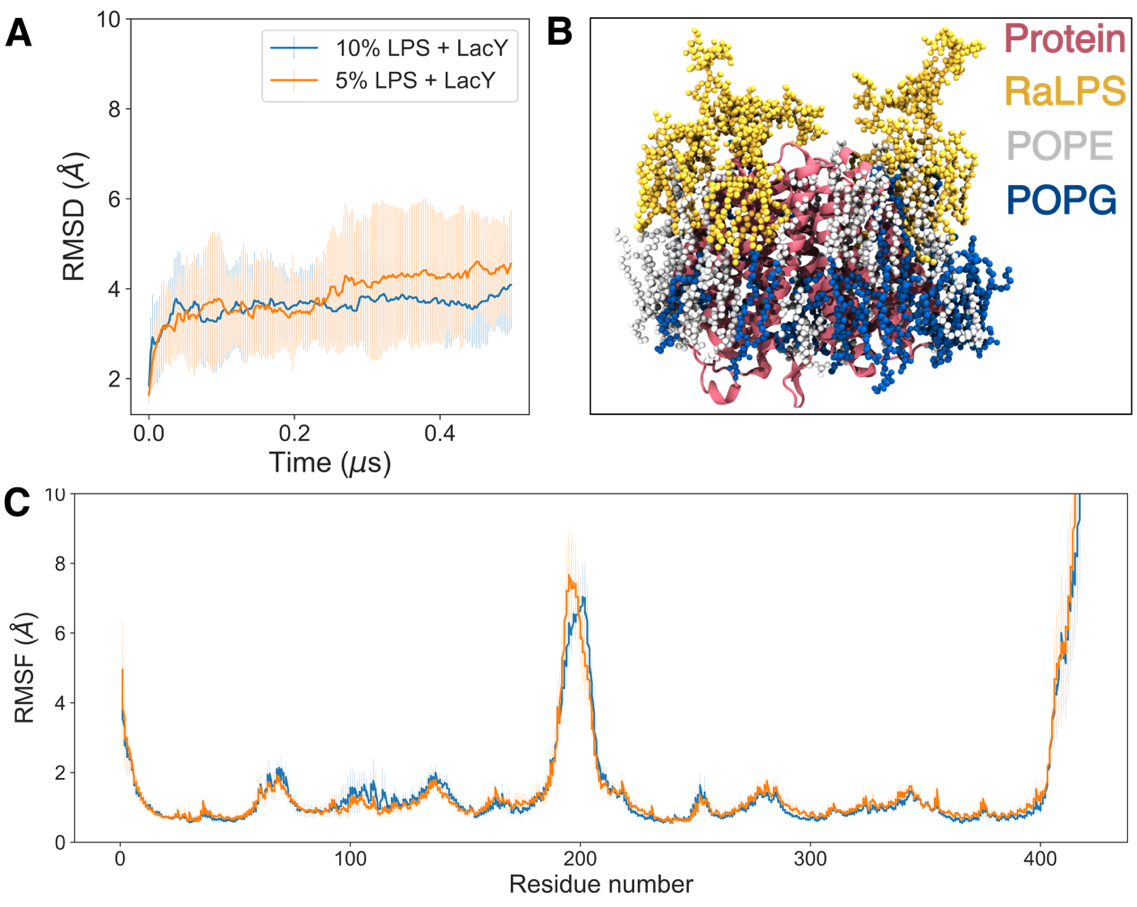


**Figure S22:** **Properties of proteins in all-atom simulations.** **A)** Backbone RMSD of individual LacY proteins with respect to conformation at t = 0 μs (10 LPS + 3 LacY + 10 PMB1 (n=2, blue), 5 LPS + 3 LacY + 20 PMB1 systems (n=2, orange)). Individual trajectories were block averaged in 2.5 ns blocks and averaged over n repeats. Error bars show 95% confidence intervals. **B)** Snapshot of a LacY protein at the end of 0.5 μs simulation. System components are represented as: LacY = pink cartoon; RaLPS = yellow vdW; POPE = gray vdW and POPG = blue vdW. **C)** Per-residue RMSF of LacY over last 0.1 μs after fitting to an average structure calculated over this time period. RMSFs were calculated for all 3 LacY proteins and averaged, error bars show 95% confidence intervals. Colour scheme is equivalent to that used in **A**.

***Table S2: PMB1 Clustering information for CG systems.*** *Clusters representing 95% of frames are shown here, along with cutoffs used and number of PMB1 molecules in each pseudo trajectory.*

| System | Inserted | | | | | | Surface-bound | | | |
| --- | --- | --- | --- | --- | --- | --- | --- | --- | --- | --- |
|  | No, of PMB1 | Cutoff (Å) | % cluster 1 | % cluster 2 | % cluster 3 | % cluster 4 | No, of PMB1 | Cutoff (Å) | % cluster 1 | % cluster 2 |
| 10% LPS CHARMM | 14 | 3.5 | 55 | 34 | 6 | 3 | 5 | 3.0 | 46 | 22 |
| 5% LPS CHARMM | 14 | 3.0 | 62 | 30 | 4 | - | 2 | 3.0 | 64 | 14 |
| 5% LPS CHARMM ext | 24 | 3.0 | 51 | 38 | 5 | 2 | 4 | 3.0 | 52 | 24 |
| 10% LPS + LacY | 21 | 3.0 | 55 | 38 | 5 | - | 1 | 2.5 | 56 | 18 |
| 5% LPS + LacY | 17 | 3.0 | 61 | 25 | 7 | 3 | 3 | 3.0 | 55 | 33 |
| 5% LPS + LacY ext | 38 | 3.0 | 54 | 31 | 7 | 3 | 3 | 3.0 | 72 | 14 |
| 5% LPS + AmtB | 21 | 3.0 | 50 | 37 | 6 | 3 | 4 | 3.0 | 69 | 20 |


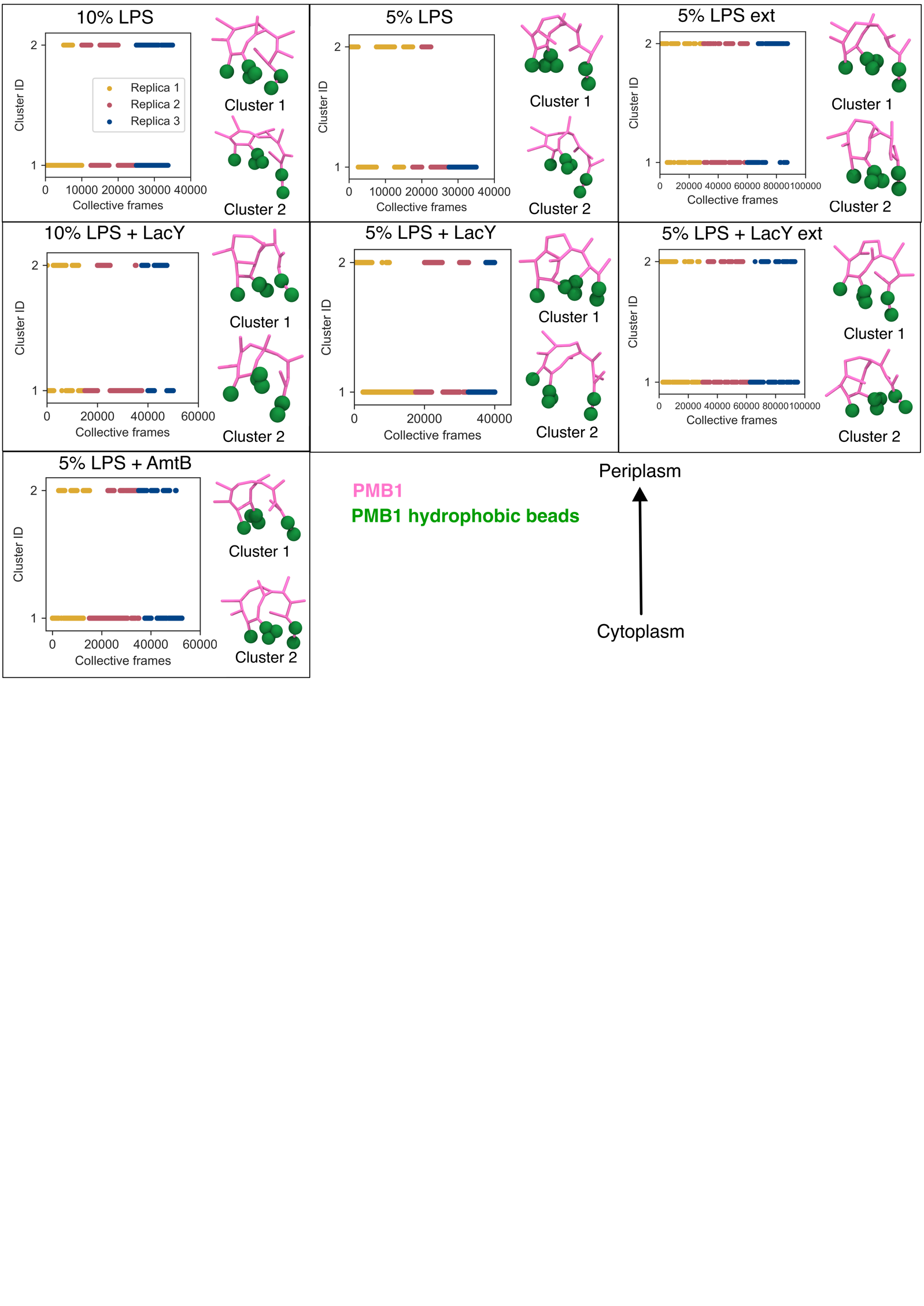


***Figure S23: Inserted PMB1 equilibrium conformations in CG systems (19-20 µs).*** *For each system type, frames for every inserted PMB1 molecule across all repeats were concatenated and clustering was done (GROMOS method, cutoffs in Table S2). In each panel we show:* ***Left****) cluster center IDs over concatenated frames, different colors represent different repeats;* ***right****) PMB1 cluster centers containing > 10% of frames, oriented with the vertical direction matching the membrane normal (periplasm at top, cytoplasm at bottom). PMB1 = pink licorice; PMB1 hydrophobic moieties = green vdW.*


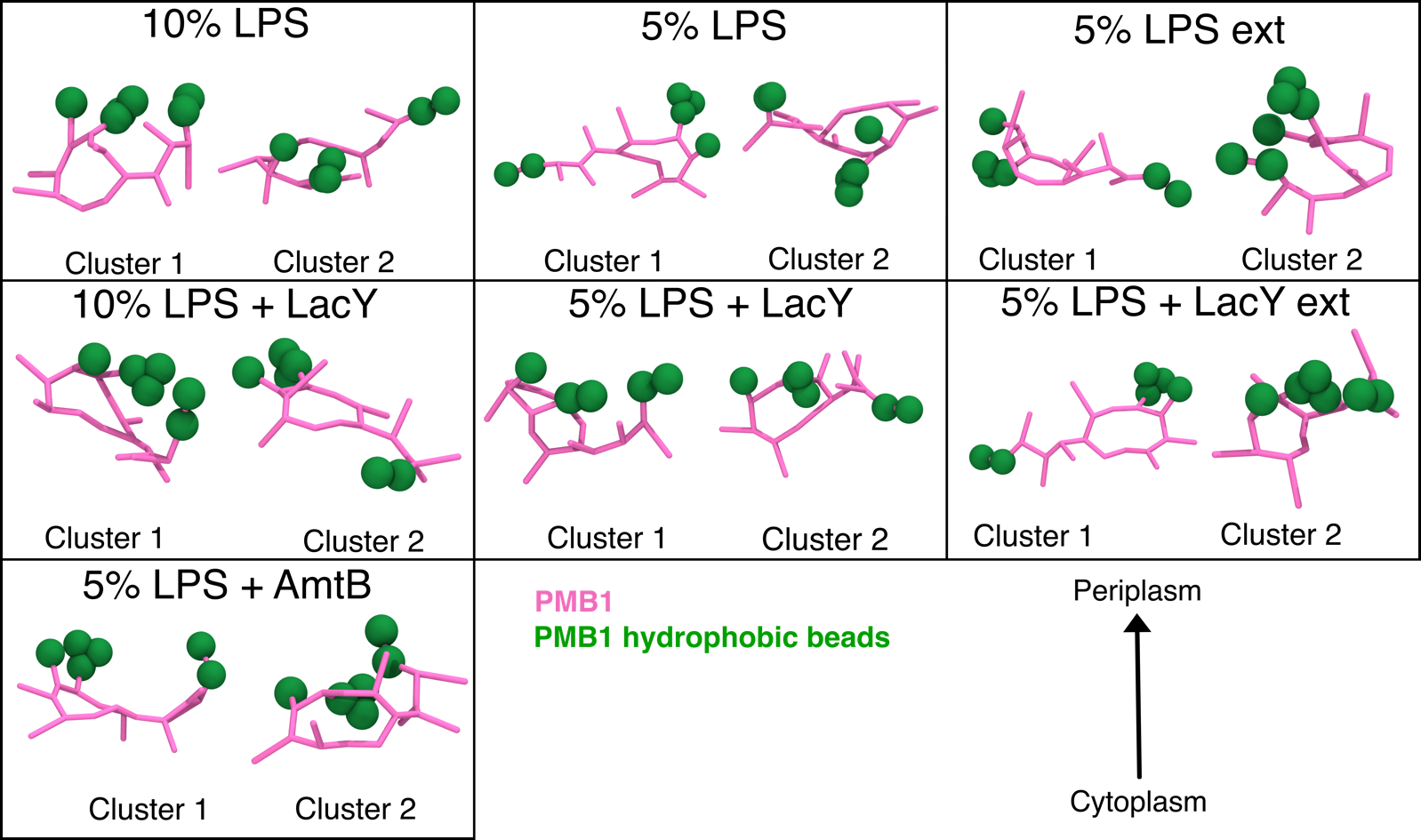


***Figure S24: LPS surface-bound PMB1 equilibrium conformations in CG systems (19-20 µs).*** *For each system type, frames for every inserted PMB1 molecule across all repeats were concatenated and clustering was done (GROMOS method, cutoffs in Table S2). In each panel we show the two most occupied PMB1 cluster centers, oriented with the vertical direction matching the membrane normal (periplasm at top, cytoplasm at bottom). PMB1= pink licorice; PMB1 hydrophobic moieties = green vdW. A) Protein-free systems, B) LacY-containing systems, C) AmtB-containing systems.*

***Table S3: PMB1 Clustering information for AA systems.*** *Populations of the first 4 clusters are shown here, along with cutoffs used and number of PMB1 molecules in each pseudo trajectory.*

| System | Inserted | | | | | |
| --- | --- | --- | --- | --- | --- | --- |
|  | No, of PMB1 | Cutoff (Å) | % cluster 1 | % cluster 2 | % cluster 3 | % cluster 4 |
| 10% LPS | 11 | 3.0 | 47 | 22 | 18 | 6 |
| 5% LPS ext | 24 | 3.0 | 44 | 23 | 15 | 6 |
| 10% LPS + LacY | 15 | 3.0 | 51 | 20 | 13 | 9 |
| 5% LPS + LacY ext | 27 | 3.0 | 38 | 25 | 9 | 9 |


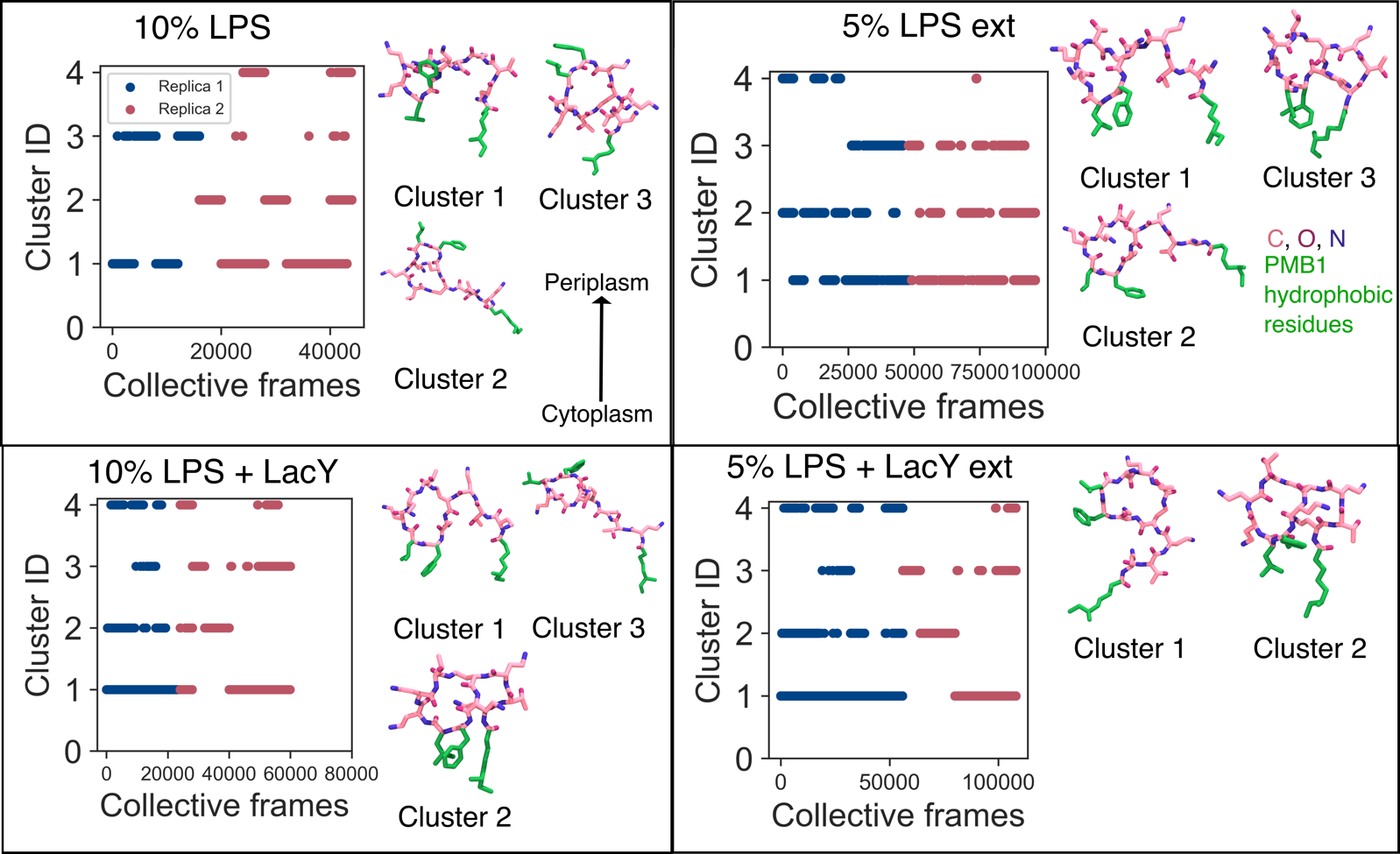


***Figure S25: Inserted PMB1 equilibrium conformations in AA systems (0.1-0.5 μs, skipped every 5 frames for tractability).*** *For each system type, frames for every inserted PMB1 molecule across all repeats were concatenated and clustering was done (GROMOS method, cutoffs in Table S3). In each panel we show:* ***Left****) cluster center IDs over concatenated frames;* ***right****) PMB1 cluster centers containing > 10% of frames, oriented with the vertical direction matching the membrane normal (periplasm at top, cytoplasm at bottom). PMB1 = licorice, CPK color scheme + pink (carbons); PMB1 hydrophobic moieties = green licorice.*

***
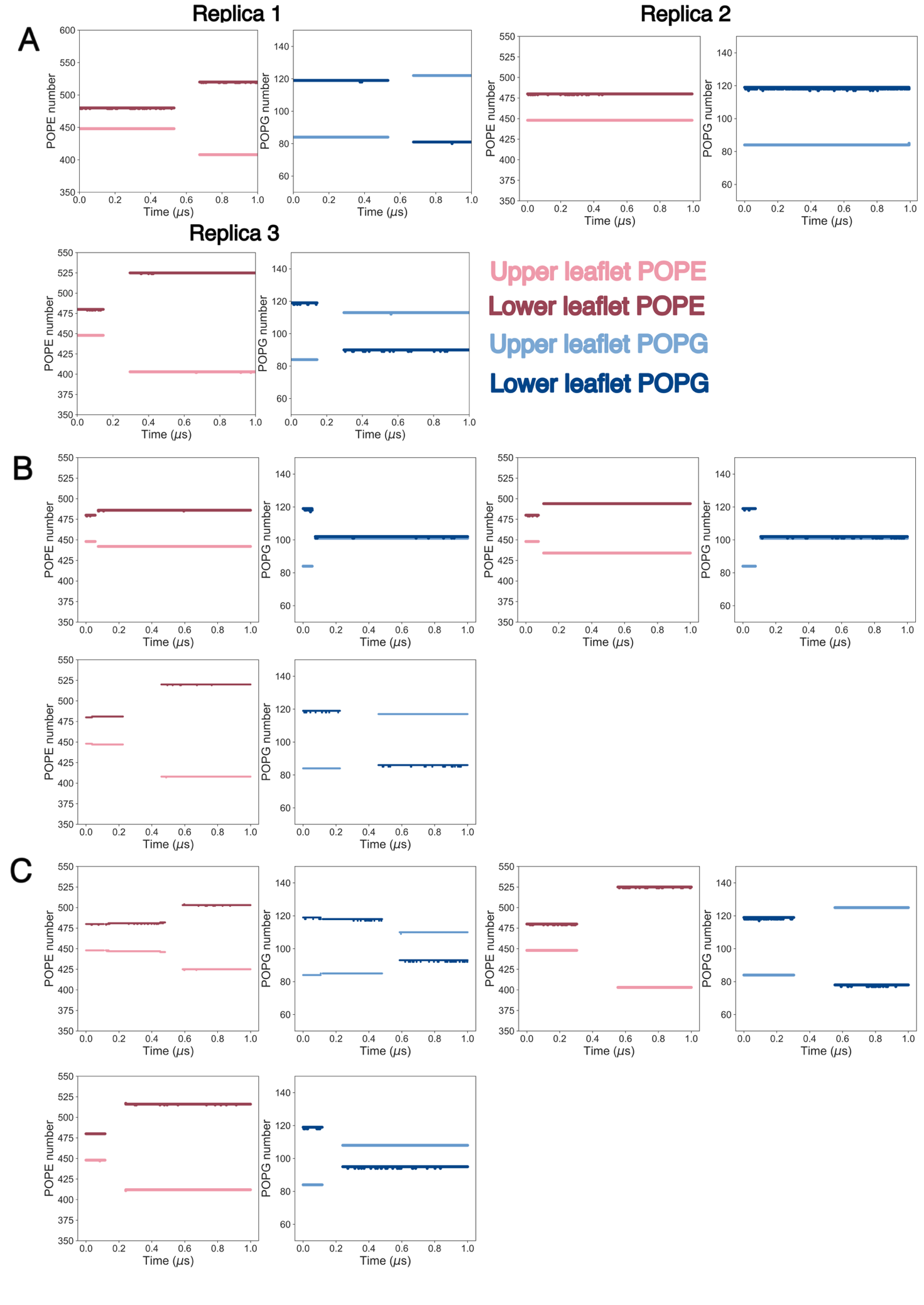
***

**Figure S26:** **Movement of PLs across the electroporated bilayer at different field strengths.** Number of PLs in each leaflet of the electroporated bilayer. **A)** TM charge = +138 e (n=3). **B)** TM charge = +140 e (n=3). **C)** TM charge = +142 e (n=3). Data are shown for: **left**) POPE (LPS-free leaflet (dark red), LPS-containing leaflet (light red)); **right**) POPG (LPS-free leaflet (dark blue), LPS-containing leaflet (light blue)). Where counts could not be determined, it was likely due to pore formation.


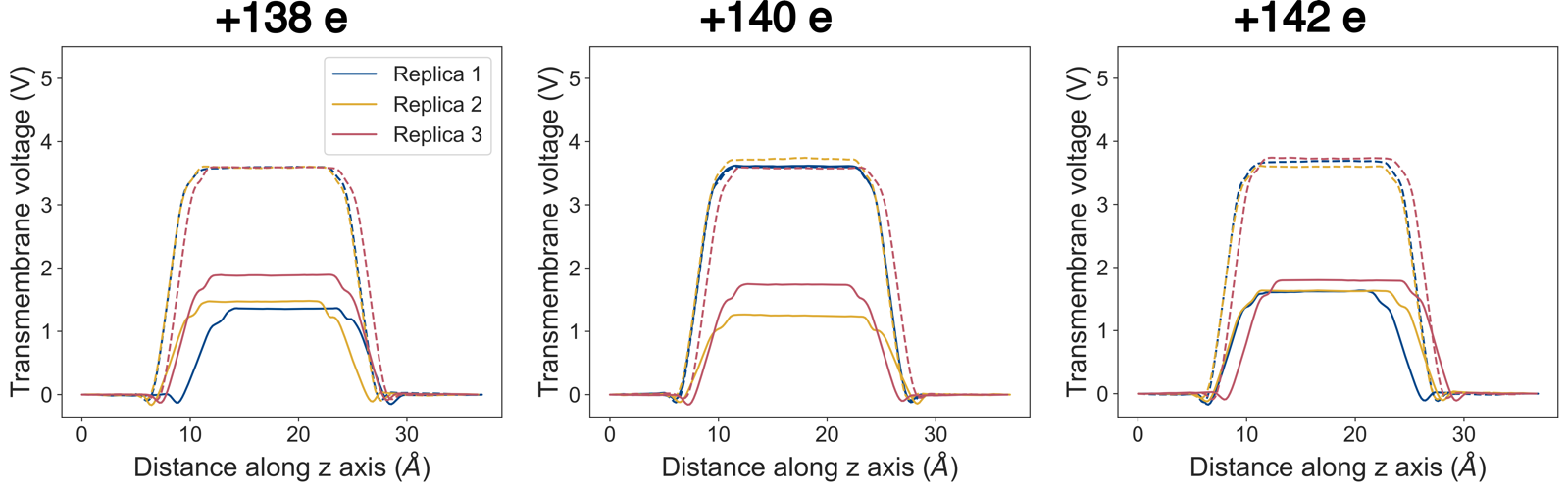


**Figure S27: TM voltage of systems before and after electroporation at different field strengths.** TM voltages were measured in 0.1 μs intervals before formation of the pore (dashed line) and after pore closure (solid line); different colors represent different repeats. **Left**) TM charge = +136 e (n=3). **Middle**) TM charge = +138 e (n=3). **Right**) TM charge = +140 e (n=3).

*
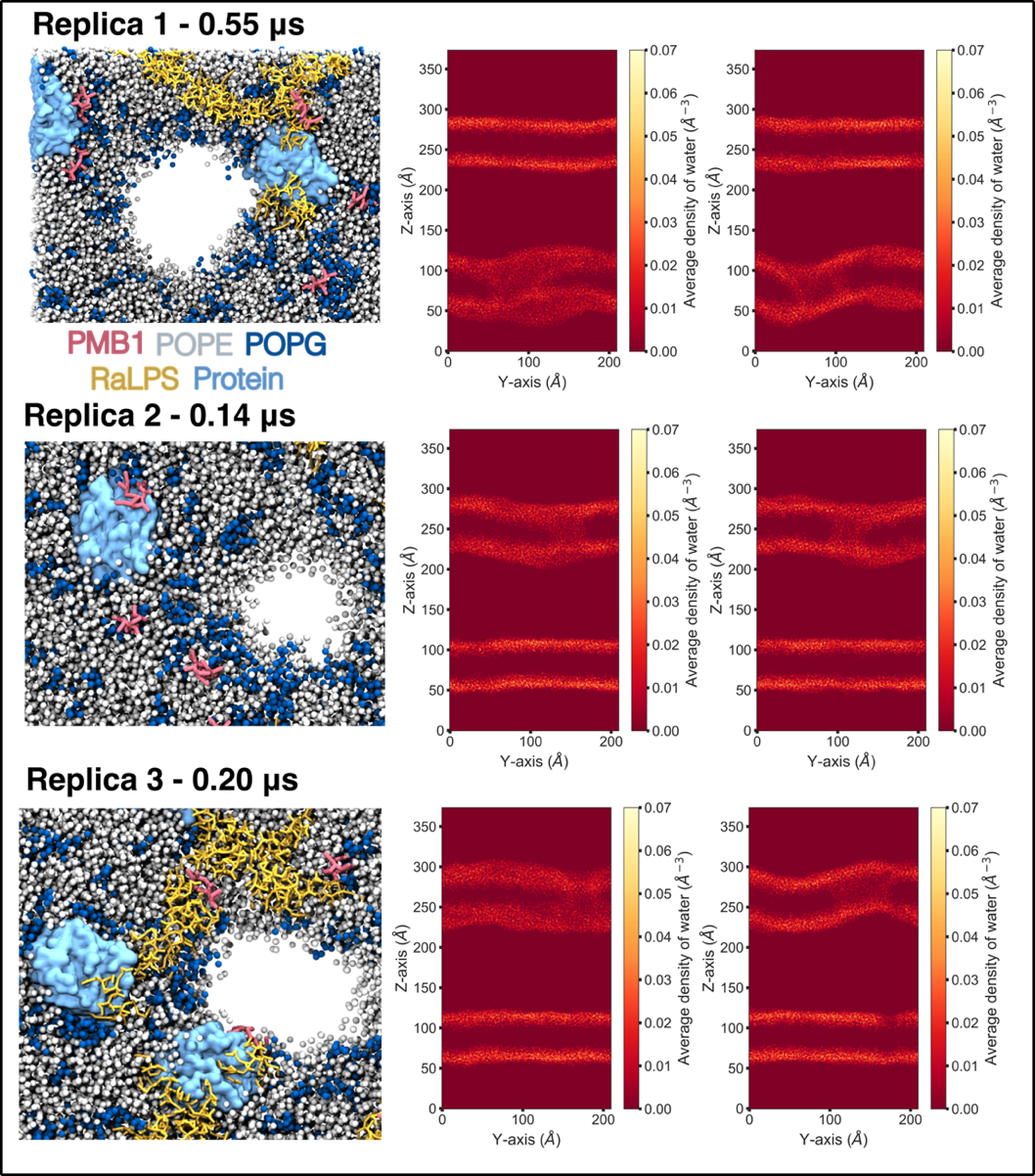
*

**Figure S28:** **Pore morphology with TM charge of +138 e (n=3). Left**) snapshot of electroporated bilayer in top-down view at time indicated. System components are represented as: RaLPS = yellow licorice; POPE = gray vdW; POPG = dark blue vdW; PMB1 = pink licorice; protein = light blue surface. Cross sections of water density within 10 Å of lipid phosphates are shown in xz (**centre**) and yz (**right**) planes at times indicated.


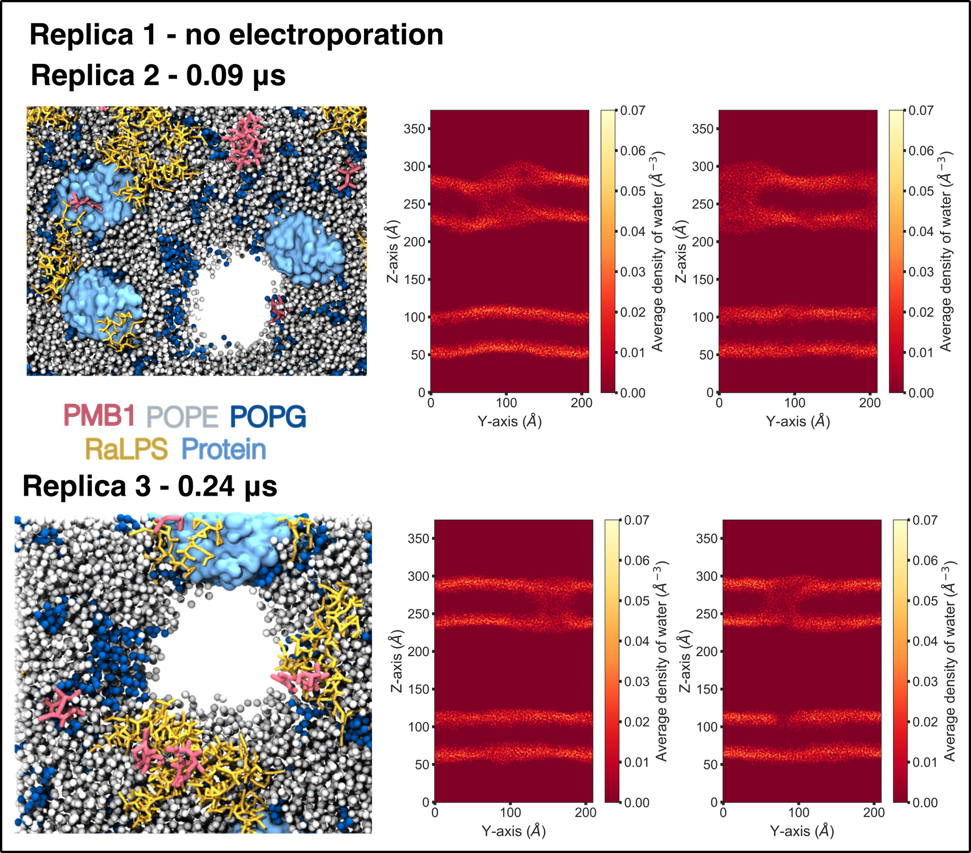


**Figure S29:** **Pore morphology with TM charge of +140 e (n=3). Left**) snapshot of electroporated bilayer in top-down view at time indicated. System components are represented as: RaLPS = yellow licorice; POPE = gray vdW; POPG = dark blue vdW; PMB1 = pinki licorice; protein = light blue surface. Cross sections of water density within 10 Å of lipid phosphates are shown in xz (**centre**) and yz (**right**) planes at times indicated.


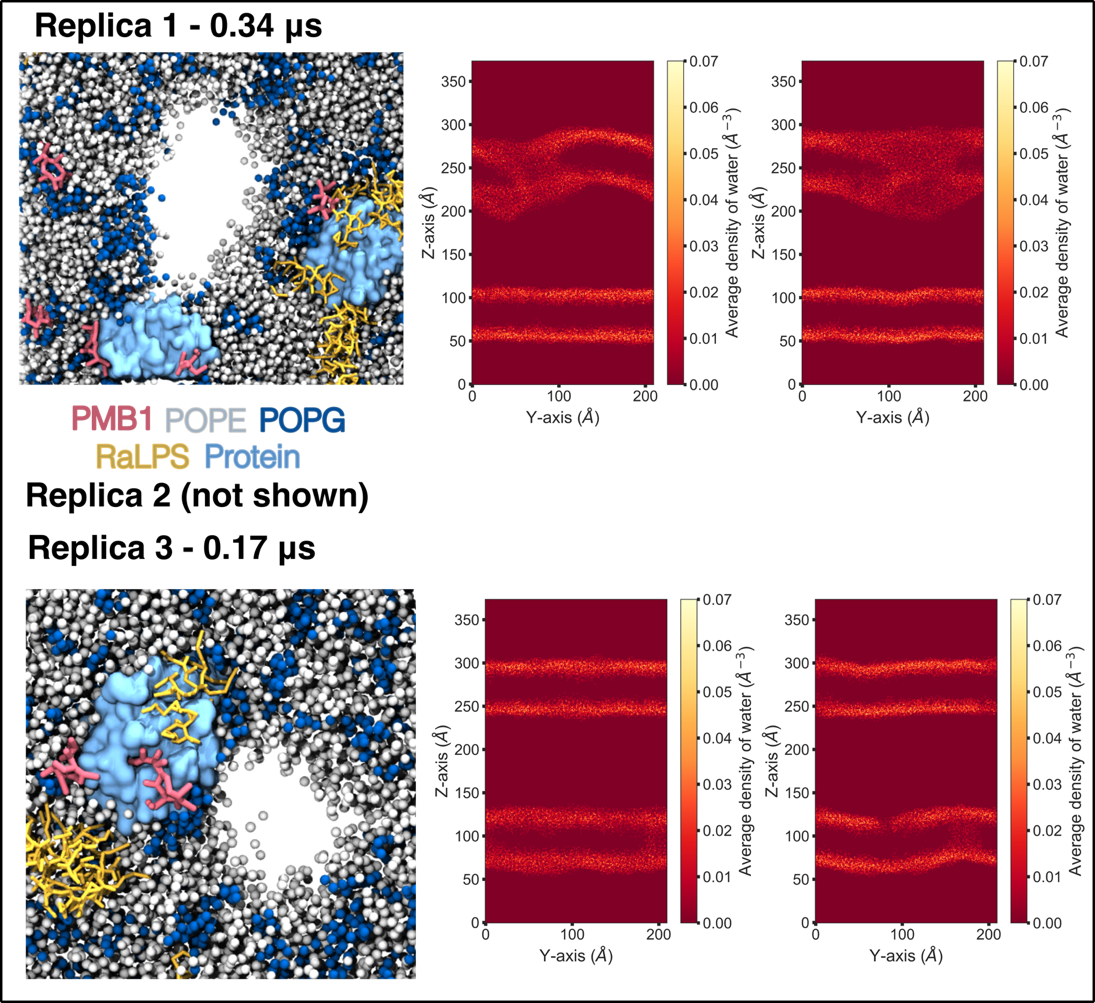


**Figure S30:** **Pore morphology with TM charge of +142 e (n=3). Left**) snapshot of electroporated bilayer in top-down view at time indicated. System components are represented as: RaLPS = yellow licorice; POPE = gray vdW; POPG = dark blue vdW; PMB1 = pink licorice; protein = light blue surface. Cross sections of water density within 10 Å of lipid phosphates are shown in xz (**centre**) and yz (**right**) planes at times indicated.
